# Incidence and Risk Factors of Platinum-Based Chemotherapy-Induced Nausea and Vomiting: A Systematic Review and Meta-Analysis

**DOI:** 10.3390/curroncol32060325

**Published:** 2025-05-31

**Authors:** Kaili Jin, Xianlei Lin, Xiaoting Xia, Huiling Ye, Dan Yang, Ying Fan, Qiuhua Sun, Rongyun Wang

**Affiliations:** 1 School of Nursing, Zhejiang Chinese Medical University, Hangzhou 310053, China; 202212210605015@zcmu.edu.cn (K.J.); xiaxiaoting66231@163.com (X.X.); 202411114111052@zcmu.edu.cn (D.Y.); 15172570346@163.com (Y.F.); sunqiuhua@zcmu.edu.cn (Q.S.); 2 Department of Oncology, Dingqiao Branch of GuangXing Hospital Affiliated to Zhejiang Chinese Medical University, Hangzhou 310053, China; linxianlei_00@163.com; 3 Department of Oncology, The First Affiliated Hospital of Zhejiang Chinese Medical University (Zhejiang Provincial Hospital of Chinese Medicine), Hangzhou 310006, China; lingxiajiudu2007@163.com

**Keywords:** platinum-based chemotherapy, chemotherapy-induced nausea and vomiting, risk factors, meta-analysis, systematic review

## Abstract

Background: Platinum-based chemotherapy significantly increases the risk of nausea and vomiting, which can impair the treatment’s efficacy and the patient’s quality of life. This meta-analysis examines the incidence and risk factors of platinum-based chemotherapy-induced nausea and vomiting (PINV) in patients treated with this chemotherapy. Methods: This systematic review and meta-analysis were conducted in accordance with the PRISMA 2020 guidelines. We conducted a literature search in the databases PubMed, Embase, Web of Science, WanFang, China Science and Technology Journal Database (VIP), China National Knowledge Infrastructure (CNKI), and Chinese Medical Association Journal Database (CMAJD) through to 20 January 2025. Studies that reported the incidence and identified risk factors of nausea and vomiting specifically in patients receiving platinum-based chemotherapy were included in the review. The data were extracted independently by two reviewers. The odds ratios (ORs) for each risk factor were calculated from the included studies. Sensitivity analyses and additional subgroup analyses were performed to ensure the robustness of our findings. Results: This meta-analysis included 32 studies involving 14,207 participants. Female sex (OR = 2.363, 95% CI = 1.363–4.096, *p* = 0.002), anxiety (OR = 1.689, 95% CI = 1.057–2.700, *p* = 0.028), fatigue (OR = 1.413, 95% CI = 1.145–1.744, *p* = 0.001), motion sickness (OR = 1.816, 95% CI = 1.266–2.605, *p* = 0.001), and a history of vomiting during chemotherapy (OR = 2.728, 95% CI = 1.468–5.069, *p* = 0.002) were significantly associated with an increased risk of PINV. Conclusion: Female sex, anxiety, fatigue, motion sickness, and a history of vomiting during chemotherapy increase the risk of PINV during platinum-based treatments.

## 1. Introduction

Chemotherapy, one of the most commonly used treatments for cancer [1,2], is a systemic treatment. It inhibits the growth of cancer cells, but it also damages normal cells and thereby causes adverse reactions and side effects, such as nausea and vomiting [3]. Platinum-based chemotherapeutic agents (cisplatin, carboplatin, and oxaliplatin) have shown significant antitumor efficacy [4,5]. However, platinum-based agents are regularly prescribed with caution due to their severe adverse effects, including platinum-induced nausea and vomiting (PINV) [6]. Cisplatin is the most emetogenic chemotherapy drug in common use, while the nausea and vomiting associated with carboplatin are moderately severe [7]. In a recent study [8], cisplatin was found to have an acute vomiting incidence rate of over 90% and a delayed nausea and vomiting incidence rate of over 50%, significantly reducing patient quality of life.

Nausea and vomiting generally occur together during chemotherapy. Nausea is a subjective feeling of discomfort, usually associated with the upper abdomen, which may lead to vomiting. Because of the subjective nature of responses to nausea, the feeling, location, duration, and intensity of nausea reported by patients tend to vary [1]. Vomiting is the discharge of stomach contents through the mouth [9]. Clinically, CINV is usually divided into three categories: acute onset (mainly related to serotonin), which occurs within 24 h after the first chemotherapy treatment; delayed onset (partly related to substance P), which occurs 24 h to several days after the initial treatment; and anticipatory onset, seen in patients who vomit in response to taste, smell, sight, thoughts, or anxiety, a history of which is secondary to a poor response to antiemetic drugs or inadequate prophylactic antiemetic measures during the previous round of chemotherapy [10,11,12].

PINV not only significantly reduces patients’ quality of life, but also adversely affects their food intake, thereby affecting their nutritional status and overall health [8,13,14]. Relevant guidelines note that uncontrolled PINV (CINV) can lead to a deterioration in nutritional status and interruptions in chemotherapy [15]. These side effects may lead to reduced compliance with chemotherapy [16], resulting in a reduction in the therapeutic dose or the complete cessation of treatment [9]. This seriously affects the efficacy of chemotherapy and leads to an increased risk of treatment failure or tumor recurrence. Therefore, proactive prevention and treatment are required for the management of PINV to avoid the clinical, quality-of-life, and economic problems that arise when PINV is poorly controlled [16]. Several studies have investigated and identified the factors that influence the occurrence of PINV. These risk factors include patient-related risk factors, such as female sex, anxiety, motion sickness, and a history of vomiting during pregnancy, as well as treatment-related risk factors, such as a history of nausea and vomiting prior to chemotherapy and specific chemotherapy regimens [17,18,19].

In this study, we performed a meta-analysis of the risk factors for PINV. The aim of this study was to systematically evaluate and synthesize the existing research data to accurately clarify the incidence and risk factors associated with PINV. These results will help to more accurately identify high-risk patient groups and provide them with targeted preventive measures, thereby improving the quality of treatment, patient well-being, and patient outcomes.

## 2. Materials and Methods

This review protocol was registered in PROSPERO (CRD42024599277), and the systematic review and meta-analysis were conducted and reported in accordance with the Preferred Reporting Items for Systematic Reviews and Meta-Analyses (PRISMA) 2020 guidelines. A completed PRISMA 2020 checklist and a flow diagram are provided in the Supplementary Materials.

### 2.1. Literature Search Strategy

To comprehensively assess the prevalence of and risk factors for nausea and vomiting in patients receiving platinum-based chemotherapy, we conducted a systematic literature search up to January 20, 2025. We searched the following databases: PubMed, Embase, Web of Science, Wanfang, China National Knowledge Infrastructure (CNKI), and China Science and Technology Journal Database (VIP). We used the following search terms: ((risk factors) OR (factors) OR (prevalence) OR (incidence) OR (relative risk) OR (predict)) AND ((platinum-based chemotherapy-induced nausea) OR (platinum-based chemotherapy-induced vomiting) OR (PINV)). In addition to the literature search in these international databases, corresponding Chinese terms were used to search Chinese databases to ensure comprehensive coverage of the literature. The references in the retrieved articles were also reviewed to identify any additional relevant studies. The detailed search strategy is shown in the Supplementary Materials.

### 2.2. Study Inclusion and Exclusion Criteria

The inclusion criteria for the studies were as follows: (1) study design: observational studies, including cross-sectional studies and prospective/retrospective cohort studies; (2) study subjects: age ≥18 years, histologically confirmed solid malignant tumors, and at least one cycle of platinum-based chemotherapy; (3) exposure factors: nausea and vomiting, with the definition and diagnostic criteria for CINV clearly stated in the literature (for example, reference to the Union for International Cancer Control [20] or the National Cancer Institute [21], which classify CINV by the severity and duration of symptoms); and (4) a calculated odds ratio (OR) value and 95% confidence interval (CI), or the availability of original data to calculate the OR and CI.

The exclusion criteria were as follows: (1) patients with severe mental illness (e.g., schizophrenia, cognitive impairment); (2) studies for which the full text was not available; (3) studies from which raw data could not be extracted or converted; (4) duplicate publications; and (5) studies published in a language other than Chinese or English.

### 2.3. Data Extraction

The data were extracted from the selected studies by two independent reviewers. A standardized data collection form was used to systematically record information on the first author, year of publication, study design, chemotherapy regimen, study area, tumor type, and any associated risk factors. Differences between reviewers were resolved through discussion until a consensus was reached. This rigorous approach ensured the accuracy and reliability of the data collected for subsequent analysis.

### 2.4. Quality Assessment

The quality of the included studies was rigorously assessed by two independent reviewers. The assessment tools were the Joanna Briggs Institute Critical Appraisal tool [22] for cross-sectional studies and the Newcastle–Ottawa Quality Scale [23] for cohort studies.

The Joanna Briggs tool includes eight criteria to evaluate the overall quality of an analytical cross-sectional study, focusing on the measurement and data analysis of the subjects, the study conditions, influencing factors, and potential confounders. Each criterion was rated as “yes”, “no”, “unclear”, or “not applicable”. Studies achieving all criteria were assigned a quality grade of A, while those meeting some criteria received a grade of B if 1–3 criteria were rated as “no”.

The Newcastle–Ottawa Scale was applied for cohort studies. This scale consists of eight items, with each item scoring 1 point except for the “inter-group comparability” item, which scores up to 2 points, culminating in a maximum score of 9 points. Studies with scores of 0–4 were considered low-quality, and studies with scores of 5–9 were considered high-quality.

This structured assessment ensured that only studies of sufficient quality were included in our analysis, enhancing the reliability of our findings regarding PINV.

### 2.5. Statistical Analysis

Statistical analyses were conducted using Stata software, version 17.0. In the quantitative analysis of effects, ORs were selected as the primary statistical measure, accompanied by 95% CIs. Heterogeneity among the studies was assessed using the Chi-squared test (test level α = 0.1) in combination with the *I*^2^ test. If *I*^2^ < 50% or *p* > 0.1, suggesting low heterogeneity, a fixed-effect model was applied. Significant heterogeneity was indicated by *I*^2^ > 50% or *p* < 0.1, prompting the use of a random-effects model. To explore the potential sources of heterogeneity, subgroup analyses were conducted on the basis of the study design, geographic region, age, and population source. A sensitivity analysis was performed by excluding each study in turn to assess the stability of the results. Publication bias was evaluated using funnel plots and the Egger test. To account for potential bias introduced by incomplete AE reporting, we conducted a complementary Bayesian meta-analysis using the Shiny-MAGEC application (Zhou et al., 2024), which adjusts for left-censoring in AE data. This one-stage model estimates the AE incidence probability using Bayesian methods that incorporate studies with partial or unreported events. The model implementation followed the approach described by Qi et al. (2024) and allowed for direct comparisons between bias-corrected and conventional estimates [24,25].

## 3. Results

### 3.1. Literature Search and Study Selection

**Figure 1:**
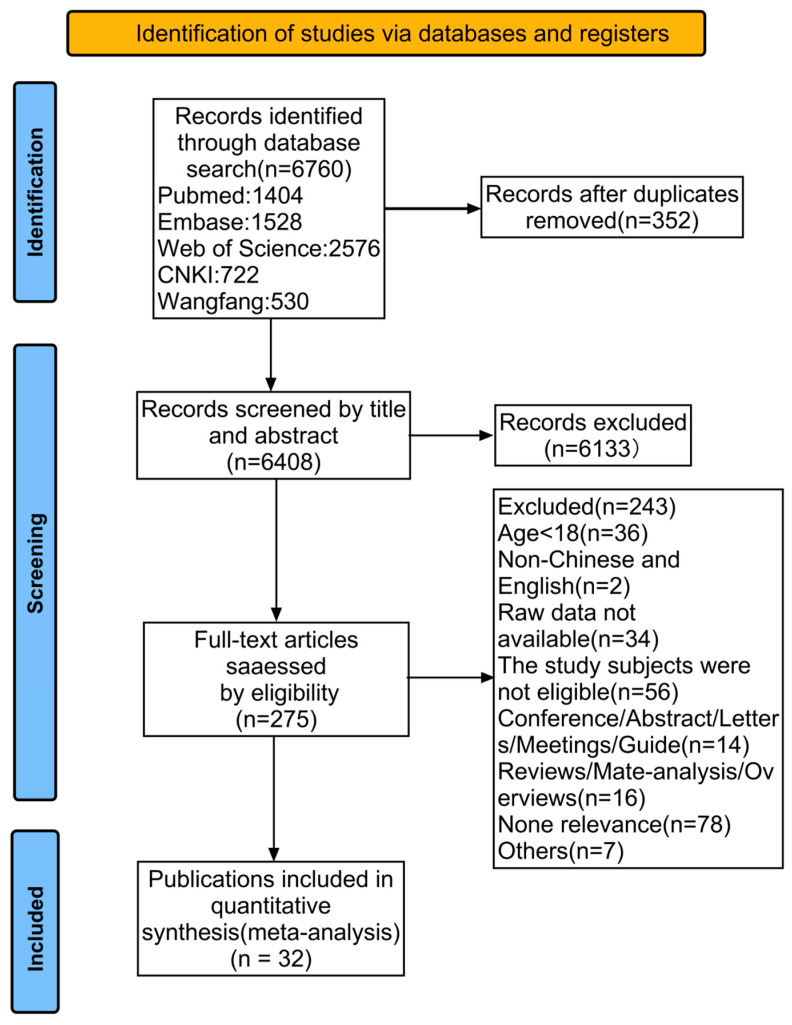
PRlSMA flowchart of the meta-analysis.

A search of databases, including PubMed, Embase, Web of Science, CNKI, and Wangfang, yielded 6760 articles for subsequent filtering. Of these, 352 duplicate studies were excluded. After a review of the title and abstract of each article and the exclusion of inconsistent document types and irrelevant documents, 275 studies were identified. After the full-text review, 36 studies were excluded because of an age mismatch, 2 were excluded because they were not in Chinese or English, 34 were excluded due to the unavailability of the full text or data, 56 were excluded due to a mismatch of study subjects, 16 were excluded due to a mismatch of study type, 78 were excluded due to irrelevance, and 7 were excluded for other reasons. Finally, 32 studies were included in the meta-analysis. The literature selection process is shown in Figure 1.

### 3.2. Characteristics of the Included Studies

The 32 included studies [18,26,27,28,29,30,31,32,33,34,35,36,37,38,39,40,41,42,43,44,45,46,47,48,49,50,51,52,53,54,55,56], which were published between 2011 and 2024, involved a total of 14,773 participants (Table 1). Among the 32 studies, 6 were cross-sectional studies [26,27,30,32,36,52] and 26 were cohort studies [18,28,29,31,33,34,35,37,38,39,40,41,42,43,44,45,46,47,48,49,50,51,53,54,55,56], with participant ages ranging from 18 to 80 years old. The studies encompassed a wide range of cancer types, which were categorized by their anatomical and physiological characteristics. The respiratory system cancers primarily included various forms of lung cancer and nasopharyngeal carcinoma, while the gastrointestinal cancers covered a range of cancers, from gastric to colorectal and pancreatic cancers. The gynecological cancers included breast and ovarian cancers. Rare cancers, such as malignant pleural mesothelioma, and other less common cancer types, such as bone and soft tissue cancers, were also considered. The characteristics of the included studies are presented in Table 1.

### 3.3. Result of Quality Assessment

The quality of the included studies was assessed using the Joanna Briggs Institute Critical Appraisal tool and the Newcastle–Ottawa Scale Quality Assessment Tool. Among the six cross-sectional studies [26,27,30,32,36,52], five studies were rated as Grade A and one study was Grade B. All 26 [18,28,29,31,33,34,35,37,38,39,40,41,42,43,44,45,46,47,48,49,50,51,53,54,55,56] cohort studies were classified as high-quality research. The details are provided in the Supplementary Materials.

### 3.4. Meta-Analysis Results

#### 3.4.1. Total Prevalence of PINV

**Figure 2:**
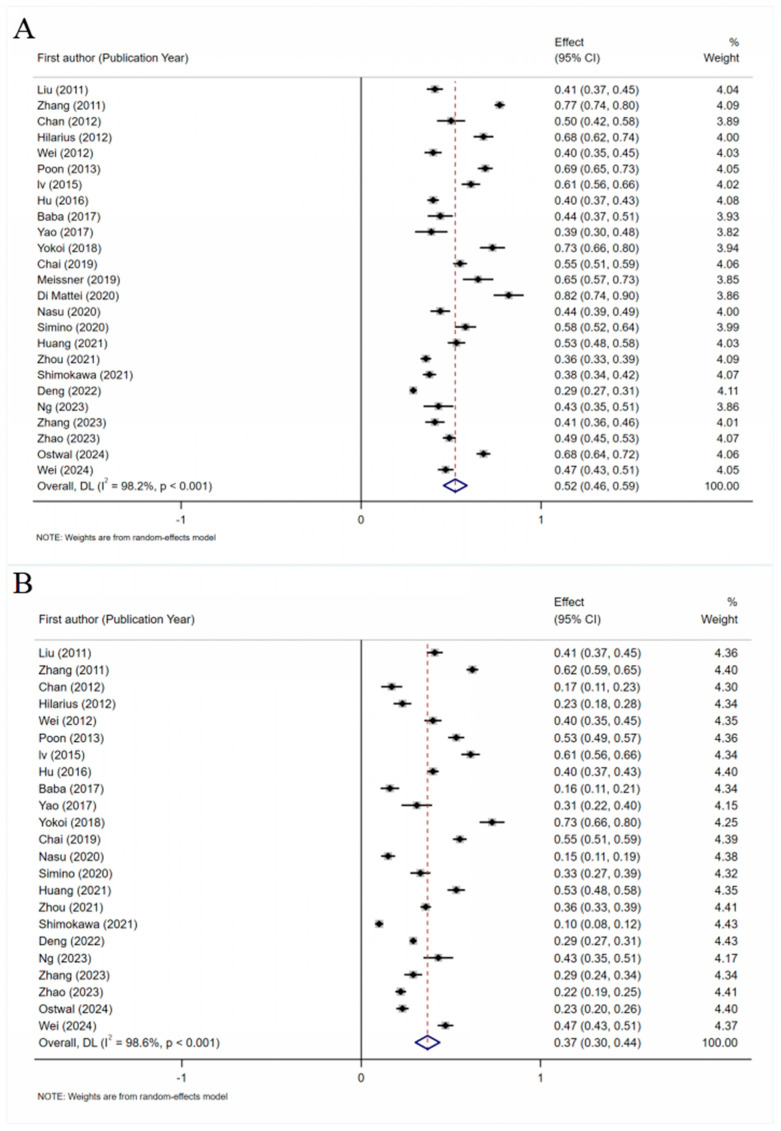
(**A**) Forest plot of the incidence of nausea in platinum-based chemotherapy patients [18,26,27,28,29,30,31,32,33,35,36,37,38,41,42,43,46,47,48,49,52,53,54,55,56]; (**B**) forest plot of the incidence of nausea in platinum-based chemotherapy patients [18,26,27,28,29,30,31,32,33,35,36,37,42,43,46,47,48,49,52,53,54,55,56].

Twenty-five articles [18,26,27,28,29,30,31,32,33,35,36,37,38,41,42,43,46,47,48,49,52,53,54,55,56] reported on the incidence of nausea. The pooled analysis showed that the incidence of nausea in platinum-based chemotherapy patients was 52% (95% CI 46–59%) (Figure 2). Twenty-three articles [18,26,27,28,29,30,31,32,33,35,36,37,42,43,46,47,48,49,52,53,54,55,56] reported on the incidence of vomiting. The pooled analysis yielded an incidence rate of 37% (95% CI 30–44%). A heterogeneity analysis revealed significant heterogeneity for both nausea (*I*^2^ = 98.2%, *p* < 0.001) and vomiting (I^2^ = 98.6%, *p* < 0.001). Both outcomes were analyzed using a random-effects model and subgroup analyses to explore the sources of this heterogeneity. The sensitivity analysis results were stable, and the Egger test (*p* > 0.05), along with symmetric funnel plots, indicated no publication bias. To account for potential underreporting of adverse events, we conducted a Bayesian meta-analysis using the MAGEC method. The pooled incidence of nausea was 52.6% (95% CI: 46.2–59.0%), and that of vomiting was 35.3% (95% CI: 27.7–43.6%). The between-study heterogeneity was moderate (SD = 0.63 for nausea; SD = 0.82 for vomiting). The 95% predictive intervals indicated considerable variability across future studies (23.4–80.5% for nausea; 8.9–75.8% for vomiting). Sensitivity analyses excluding censored data yielded similar results, supporting the robustness of the estimates. The detailed model outputs and Bayesian forest plots are provided in Supplementary Figures S118 and S119.

Subgroup analyses were conducted to explore the sources of heterogeneity. Detailed descriptions of the results are provided in the Supplementary Materials. The analysis results stratified by geographical region showed that the incidence of nausea among Asians was reported at 51% (95% CI 44–58%), with an incidence of vomiting of 37% (95% CI 30–44%). For Europeans, the incidence of nausea was considerably higher, at 82% (95% CI 74–90%). South Americans showed an incidence of nausea of 33% (95% CI 52–64%) and an incidence of vomiting of 33% (95% CI 27–39%). The results of a subgroup analysis based on chemotherapy regimens showed that the incidence of nausea was 51% (95% CI: 45–58%) in patients receiving cisplatin-based regimens, 64% (95% CI: 43–86%) in those receiving carboplatin-based regimens, 41% (95% CI: 37–46%) in those receiving oxaliplatin-based regimens, and 54% (95% CI: 41–66%) in the group receiving other regimens. The pooled incidence of vomiting was 45% (95% CI: 34–56%) in patients receiving cisplatin-based regimens, 19% (95% CI: 11–27%) in those receiving carboplatin-based regimens, 21% (95% CI: 10–32%) in those receiving oxaliplatin-based regimens, and 41% (95% CI: 31–50%) in the group receiving other regimens. The results of subgroup analyses by cancer type revealed that the incidence of nausea in lung cancer patients was 53% (95% CI 26–79%), while the incidence of nausea in breast cancer and gastrointestinal tumor patients was 52% (95% CI: 44–59%) and 43% (95% CI: 32–55%), respectively. The incidence of vomiting was 48% (95% CI: 32–63%) in patients with lung cancer, 48% (95% CI: 43–54%) in patients with breast cancer, and 13% (95% CI: 6–20%) in patients with gastrointestinal tumors.

#### 3.4.2. Factors Influencing PINV

##### General Factors

**Figure 3:**
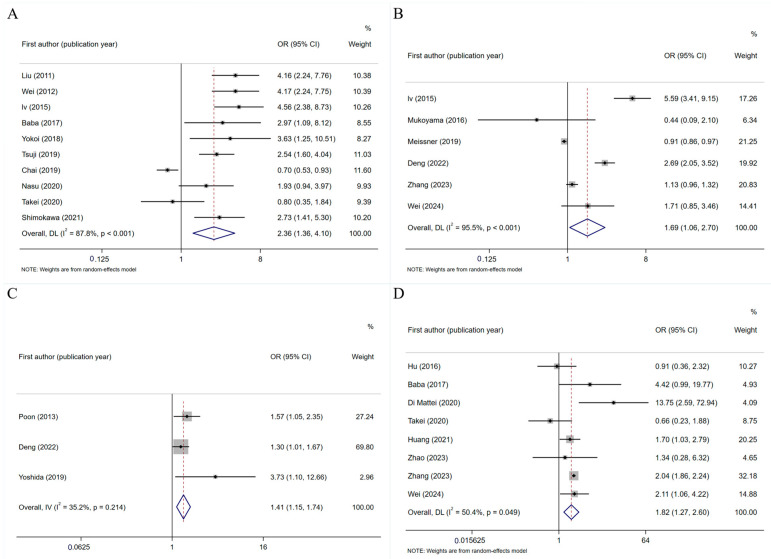
(**A**) Forest plot of the association between female gender and PINV [18,26,30,32,35,37,39,42,44,48]; (**B**) forest plot of the association between anxiety and PINV [32,34,38,49,53,56]; (**C**) forest plot of the association between fatigue and CINV [31,40,49]; and (**D**) forest plot of the association between motion sickness and PINV [33,35,41,44,46,53,54,56].

Ten studies [18,26,30,32,35,37,39,42,44,48] reported that female sex is a risk factor for PINV. The meta-analysis results showed that women are more likely to experience nausea and vomiting during chemotherapy (OR = 2.363, 95% CI: 1.363–4.096, *p* = 0.002, Figure 3A). Six studies [32,34,38,49,53,56] reported that anxiety is a risk factor for PINV, and the meta-analysis results showed that patients with anxiety during chemotherapy had a significantly increased risk of nausea and vomiting (OR = 1.689, 95% CI: 1.057–2.700, *p* = 0.028, Figure 3B). Three studies [31,40,49] documented fatigue as a risk factor for PINV, and the results of the meta-analysis showed that fatigue increased the risk of nausea and vomiting in patients receiving platinum-based chemotherapy (OR = 1.413, 95% CI: 1.145–1.744, *p* < 0.001, Figure 3C).

The meta-analysis results showed that the following factors were not associated with an increased risk of PINV: age [26,27,30,32,33,35,37,38,39,41,42,43,44,47,49,52,53,54] (OR = 1.048, 95% CI: 0.978–1.123, *p* = 0.186, Supplementary Figure S17), male sex [27,43,53,54] (OR = 0.688, 95% CI: 0.460–1.028, *p* = 0.068, Supplementary Figure S26), body mass index [38,39,42,44,52] (OR = 1.235, 95% CI: 0.702–2.170, *p* = 0.464, Supplementary Figure S45), alcohol consumption [26,27,30,33,35,37,42,43,44,49,53,54] (OR = 0.846, 95% CI: 0.677–1.058, *p* = 0.143, Supplementary Figure S41), smoking status [42,43,44] (OR = 1.220, 95% CI: 0.828–1.795, *p* = 0.314, Supplementary Figure S50), and performance status [36,39,44] (OR = 1.338, 95% CI: 0.581–3.078, *p* = 0.494, Supplementary Figure S73). The details are shown in the Supplementary Files.

##### Disease-Related Factors

**Figure 4:**
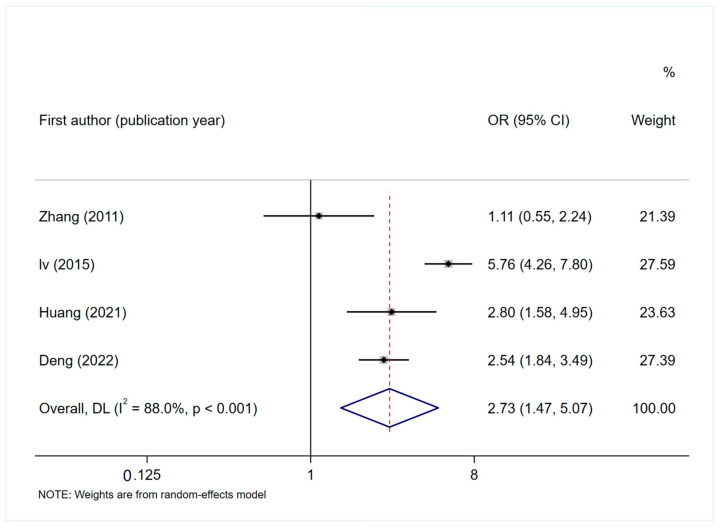
Forest plot of the association between history of vomiting during chemotherapy and PINV [27,32,46,49].

Eight studies [35,41,44,46,53,54,56] reported motion sickness as a risk factor for PINV. The analysis results showed that motion sickness significantly increased the risk of nausea and vomiting (OR = 1.816, 95% CI: 1.266–2.605, *p* = 0.001, Figure 3D). Four studies [27,32,46,49] reported that a history of vomiting during chemotherapy is a risk factor for PINV. The results of the meta-analysis showed that patients with a history of vomiting during chemotherapy had higher risk factors for vomiting and nausea (OR = 2.728, 95% CI: 1.468–5.069, *p* = 0.002, Figure 4).

The meta-analysis results also showed that the following factors were not associated with an increased risk of PINV: the course of chemotherapy [32,45,46,47] (OR = 1.677, 95% CI: 0.443–6.349, *p* = 0.761, Supplementary Figure S78) and the number of chemotherapy sessions [26,30,53] (OR = 0.963, 95% CI: 0.749–1.238, *p* = 0.767, Supplementary Figure S85). The details are shown in the Supplementary Files.

##### Treatment-Related Factors

The meta-analysis results showed that the following factors were not associated with an increased risk of PINV: chemotherapy regimen [27,43,45,47,49,54,56] (OR = 1.290, 95% CI: 0.839–1.985, *p* = 0.246, Supplementary Figure S96), prechemotherapy nausea [38,52,53,56] (OR = 1.708, 95% CI: 0.887–3.290, *p* = 0.110, Supplementary Figure S101), expectation of nausea [38,41,49] (OR = 1.859, 95% CI: 0.793–4.358, *p* = 0.154, Supplementary Figure S108), and antiemetic regimen [33,38,42,48,53] (OR = 1.106, 95% CI: 0.490–2.496, *p* = 0.808, Supplementary Figure S113). The details are shown in the Supplementary Files.

#### 3.4.3. Heterogeneity Analysis

In this meta-analysis, heterogeneity testing was meticulously performed to evaluate the variability in study outcomes for various risk factors associated with PINV. The I^2^ statistics revealed that certain factors demonstrated little to no heterogeneity, suggesting consistent findings across the studies for factors such as smoking, fatigue, motion sickness, and alcohol consumption.

Significant heterogeneity was observed in certain factors, including BMI, performance status, and number of chemotherapy sessions, with age groups identified as a primary source of this heterogeneity. The corresponding figures are provided in the Supplementary Materials. This suggests that the impacts of these factors on nausea and vomiting may vary significantly across different age groups. Additionally, risk factors such as male sex showed significant heterogeneity by geographic region. This suggests that regional differences in medical practice, patient demographics, or environmental factors may influence the impact of these factors on nausea and vomiting outcomes. The corresponding figures are provided in the Supplementary Materials. The chemotherapy regimen risk factor exhibited significant heterogeneity across different study designs. The effect of an expectation of nausea varied with the chemotherapeutic agent, while the impact of the antiemetic regimen differed across tumor types.

Subgroup and regression analyses were performed on a combination of age, female sex, anxiety, chemotherapy course, history of vomiting during chemotherapy, and prechemotherapy nausea. However, no sources of heterogeneity in these factors were identified. The lack of detectable sources suggests that the variability in the effects of these factors may be influenced by more subtle or unmeasured variables not captured in the curves. We refer the reader to the Supplementary Materials for detailed information.

#### 3.4.4. Publication Bias and Sensitivity Analysis

Sensitivity analyses, funnel plots, and Egger tests were conducted on influencing factors that were represented in three or more studies to assess publication bias. The symmetry observed in the funnel plots suggested an absence of publication bias, corroborated by the Egger test results (*p* > 0.05). These findings indicate no significant publication bias in the studies included in our meta-analysis (refer to the Supplementary Materials). This robust assessment enhances the credibility of our results, providing confidence in the generalizability and reliability of the identified risk factors for CINV. The corresponding figures are provided in the Supplementary Materials.

## 4. Discussion

CINV is among the most distressing side effects of cancer treatment, severely impacting patients’ quality of life and affecting their chemotherapy compliance [16]. Despite the high prevalence of CINV, its treatment remains difficult [57], partly because platinum-based drugs are currently the most widely used and most emetogenic anticancer drugs [7]. Therefore, a better understanding of the mechanisms underlying PINV is critical for the development of more effective prevention and treatment strategies. This study included 32 original studies with 14,773 subjects. The pooled analysis showed that the incidence of nausea in patients receiving platinum-based chemotherapy was 52%, and the incidence of vomiting was 37%. In the real world, only 68% of patients receiving platinum-based chemotherapy use the triple antiemetic regimen as recommended by the guidelines, and, among them, 31% still report moderate to severe nausea or vomiting [58]. Therefore, the management of PINV needs to be improved; it is particularly important to optimize treatment regimens and strengthen the monitoring of high-risk patients.

Our subgroup analysis results showed that, compared with patients in Asia, patients in Europe have a higher probability of PINV. This may be because of the presence of genetic polymorphisms and differences in the genes of inducible enzymes and neurotransmitter receptors in different populations, which may affect the induction of chemotherapeutic drugs and the tendency toward adverse reactions to chemotherapy. For example, some studies reported that certain genotypes (such as 5-HT3 receptor polymorphisms) [59] in European populations may make these patients more sensitive to chemotherapy drug-induced reactions. Additionally, differences in diet may affect how chemotherapy drugs are metabolized in the body. European diets are typically high in fat and dairy products, while Asian diets are generally plant-based and lower in fat. Diets high in fat may lead to drug metabolism disorders [60], exacerbating adverse reactions in the digestive system to chemotherapy drugs, which can lead to more pronounced symptoms of nausea. The results suggest that for patients in high-risk areas, we should strengthen the pre-identification and systematic screening of high-risk groups for CINV, and that we should incorporate “region” as a factor in the construction of the CINV risk prediction model in order to improve the adaptability and predictive efficacy of the model in the actual clinical setting. Clinical management strategies should reflect region-specific responsiveness; physiological differences between different races and regions, as well as the accessibility of medical resources, should be fully considered in the development of regionalized or population-specific CINV prevention and treatment strategies or guidelines [61].

Our analysis showed that the incidence of PINV varied considerably across different platinum-based chemotherapeutic regimens. Similarly, our study found significant differences in the incidence of nausea and vomiting by tumor type. The incidence was higher (≥48%) in patients with lung cancer, breast cancer, and gastrointestinal cancers. This may also be due to the fact that chemotherapeutic regimens differ for different tumors; for example, lung cancer is more often treated with cisplatin-based combination regimens [62], which are associated with an increased risk of CINV. Patients receiving chemotherapy with cisplatin should receive a combination of aprepitant on days 2 and 3, and dexamethasone on days 2 to 4 [63]. In contrast, carboplatin, with its relatively lower risk, may warrant a more individualized approach depending on patient-specific risk factors. These results underscore the importance of tailoring antiemetic strategies to the specific platinum compound used, rather than applying a uniform regimen across all platinum agents [1,62,64].

In our study, the results showed that female sex, a previous history of vomiting, and motion sickness were all significant risk factors for PINV. This gender difference may be related to the higher levels of estrogen in women and their greater sensitivity to the serotonin pathway, thereby increasing their susceptibility to emetic stimuli [65]. Although female gender has been associated with poorer results in a large number of antiemetic trials, the combined results from two phase III randomized trials of aprepitant suggested that this gender difference disappeared when aprepitant was added to a 5-HT3 receptor antagonist and dexamethasone, i.e., males and females fared equally well in the study arm receiving aprepitant [66]. Previous experiences of nausea and vomiting (e.g., pregnancy vomiting, a history of motion sickness, chemotherapy-related vomiting) may sensitize the neural pathways in the central nervous system that regulate the vomiting reflex, lowering the patient’s activation threshold for nausea and vomiting [67].

Anxiety has also been suggested as a risk factor for PINV. Anxiety can exacerbate nausea and vomiting by affecting the neuroendocrine system, inducing anticipatory vomiting or even creating a state of persistent neurologic overreaction [68,69]. One case report described persistent nausea that provoked anxiety, leading to conditioned anticipatory nausea and vomiting that, in turn, aggravated the patient’s CINV [70]. In patients with tumors associated with anxiety, an antiemetic regimen based solely on the emetogenic grade of the chemotherapeutic agent may not be sufficient to achieve effective prevention. Aybar et al. [71] showed that relaxation and breathing exercises can reduce the frequency of nausea, vomiting, and dry heaving after chemotherapy. Molassiotis et al. [72] noted that psychological interventions such as Cognitive Behavioral Therapy (CBT), yoga, and Guided Imagery can all improve nausea and vomiting in patients with anxiety. It is suggested that clinics should routinely conduct an anxiety assessment prior to chemotherapy, and that the application of antiemetic drugs could be combined with psychological interventions such as CBT, positive thinking training, or relaxation techniques [73,74]. We suggest that techniques such as meditation, relaxation, and biofeedback be offered as complementary therapy for PINV [75].

Our study found that fatigue is a risk factor for PINV. Chemotherapy-related fatigue is often accompanied by the activation of the immune system and the release of inflammatory factors (e.g., IL-6, TNF-α); these inflammatory factors may further stimulate the vomiting center, exacerbating the nausea and vomiting response [76]. In addition, fatigue may disrupt the neurotransmitter balance and induce sleep disorders and circadian rhythm disorders, thus reducing the patient’s tolerance to the side effects of chemotherapy [77,78]. Parisa et al. [79] conducted a meta-analysis of the use of Cognitive Behavioral Therapy to improve fatigue and showed that such therapy significantly improved fatigue in chemotherapy patients. Studies have shown that moderate aerobic exercise (e.g., walking, yoga) and strength training (e.g., light weight lifting) for patients with high levels of fatigue can enhance patients’ physical fitness and their ability to adapt to the side effects of chemotherapy [77,78]. Therefore, it is recommended to clinically consider fatigue as an intervenable risk factor for PINV by assessing patients’ fatigue levels before the start of chemotherapy and dynamically during chemotherapy.

Motion sickness was found to be a significant factor for PINV. Patients with motion sickness are highly sensitive to vestibular system stimulation, and their inner ear vestibular system is prone to generating confusing signals when sensing changes in body position, thus activating the medullary vomiting center and triggering nausea and vomiting [80]. Chemotherapy causes excitation of the chemoreceptor area through the activation of 5-HT_3_ receptors in the gastrointestinal tract and central nervous system which, in turn, initiates the vomiting reflex [81,82]. In patients with a predisposition to motion sickness, a triple prophylactic regimen may be considered, with the addition of adjunctive medications such as olanzapine, depending on the response [83]. In addition, patients are advised to avoid stimuli that may elicit vestibular responses, such as rapid head rotation and bright flashing lights during treatment, to reduce vestibular loading [68].

This study had some significant advantages in terms of its design. The study focused on the incidence and risk factors of nausea and vomiting in patients receiving platinum-based chemotherapy, providing important baseline data in this field. This was also the world’s first systematic meta-analysis of the incidence and risk factors of nausea and vomiting in patients receiving platinum-based chemotherapy. The quality of the included studies and the reliability of the results were ensured by the detailed search of multiple databases and strict selection of the included literature. Additionally, the included studies were strictly limited to cohSort studies and cross-sectional studies to improve the scientific nature and stability of the data; this ensures a high degree of reliability and provides a valuable reference for the clinical prevention and management of PINV.

Many of the PINV risk factors identified in this study are also reflected in existing CINV prediction tools. For example, the Hesketh model emphasizes the emetogenic potential of chemotherapeutic regimens [84]. The Multinational Association of Supportive Care in Cancer (MASCC) scoring system incorporates multiple clinical variables such as gender, previous history of CINV, and previous history of motion sickness [85]. Our results not only validate some of the known risk factors in these classical models (e.g., gender, anxiety, history of prior nausea and vomiting), but also identify further risk factors, such as fatigue, that have not been widely incorporated into traditional models. This result provides an important addition to the existing modeling base. In the future, clinics may consider integrating the key risk factors identified in this study into existing prediction tools to improve the identification of high-risk patients and optimize individualized prevention and treatment strategies.

However, this study has several limitations. First, data from the Oceania population were not included, which may affect the global applicability and representativeness of the results. Second, this study only included cross-sectional studies and cohort studies, which ensured the controllability of the data; however, some potentially relevant studies may have been missed. Although randomized controlled trials (RCTs) generally provide high-level evidence, our systematic search did not identify any RCTs that met the predefined inclusion criteria. As a result, only cohort and case–control studies were included in this analysis. This limitation may restrict the strength of causal inferences, and we recommend that future reviews incorporate eligible RCT data when available, to enhance the robustness of evidence. Additionally, while the source of heterogeneity could be identified in most studies through meta-analysis, it could not be identified in some studies. To improve the universality and comprehensiveness of the current results, future studies should include data from a wider range of geographical regions and studies with diverse research designs.

## 5. Conclusions

Our study found a higher incidence of nausea and vomiting in patients receiving platinum-based chemotherapy. The results revealed that specific demographic characteristics (e.g., female sex, anxiety, fatigue) and previous histories (e.g., history of vomiting during chemotherapy, history of motion sickness) are associated with an increased risk of nausea and vomiting in patients undergoing platinum-based chemotherapy.

## Figures and Tables

**Table 1:** Characteristics of the studies included in this review.

First Author	Publication Year	Study Region	Study Design	Sample (Total-Male-Female)	Incidence of Nausea (%)	Incidence of Vomiting (%)	Average Age, y	Tumor Type	Chemotherapeutic Regimen	Influencing Factor
Liu [26]	2011	China	Cross-sectional study	459-286-173	40.52%	40.52%	62.2	Lung cancer	GP (Gemcitabine + Cisplatin), DP (Docetaxel + Cisplatin), TP (Paclitaxel + Cisplatin), NP (Vincristine + Cisplatin), or EP (Etoposide + Cisplatin)	Gender, age, alcohol consumption, platinum dosage
Zhang [27]	2011	China	Cross-sectional study	831-338-493	77.10%	62.30%	53	Small-cell lung cancer	Cisplatin(≥50 mg/m^2^), Carboplatin, Oxaliplatin(≥50 mg/m^2^)	Chemotherapy regimen, history of vomiting during pregnancy, anxiety, combined use of other antiemetic drugs, digestive system tumors
Chan [28]	2012	Singapore	Cohort study	156-88-68	50.00%	16.70%	60	Lower gastrointestinal malignancies	XELOX (Capecitabine + Oxaliplatin)	Oxaliplatin (<mg/m^2^), fewer than three risk factors
Hilarius [29]	2012	Japan	Cohort study	275-85-790	68.00%	23.00%	56	Breast cancer, lung cancer, colorectal cancer	Carboplatin-based chemotherapy regimens	Use of antiemetic treatment
Wei [30]	2012	China	Cross-sectional study	400-235-165	39.80%	39.80%	61.5 ± 2.5	Lung cancer	NP, TP, DP (Docetaxel + Cisplatin), GP or EN (Etoposide + Cisplatin)	Age, female sex, alcohol consumption, dosage of platinum/cisplatin
Poon [31]	2013	Singapore	Cohort study	473-197-276	69.10%	52.70%	55	Gastrointestinal cancer, breast cancer, head and neck cancer	XELOX, Cisplatin-based regimens	Fatigue
lv [32]	2015	China	Cross-sectional study	345-178-167	60.57%	60.57%	54 ± 4.5	Colorectal cancer, pancreatic cancer, gastric cancer, esophageal cancer, breast cancer, bladder cancer, liver cancer, colon cancer	Platin-based chemotherapy regimens	Age, female, history of CINV, anxiety, pain, chemotherapy cycles
Hu [33]	2016	China	Cohort study	881-472-390	40.00%	40.00%	56	Lung cancer, breast cancer, head and neck squamous cell carcinoma, colon cancer, gynecologic cancer, stomach cancer, and others	Carboplatin, Cisplatin	Femal sex, age, alcohol consumption, history of motion sickness, BSA, emetogenicity of chemotherapy, antiemetic regimens
Mukoyama [34]	2016	Japan	Cohort study	55-33-22	NR	NR	61.8 ± 8.6	Ovarian cancer, cervical cancer, endometrial cancer, small-cell lung cancer, non-small-cell lung cancer, malignant pleural mesothelioma	Carboplatin + Paclitaxel, Carboplatin + Docetaxel, Cisplatin + 5-Fluorouracil, Cisplatin + Pemetrexed, Cisplatin + Gemcitabine, Cisplatin + Etoposide, Cisplatin + Vinorelbine, Cisplatin + Tegafur, Gimeracil, and Oteracil, Carboplatin + Pemetrexed, Carboplatin + Gemcitabine, Carboplatin + Etoposide, Carboplatin + Paclitaxel + Bevacizumab	Female, use of non-steroidal anti-inflammatory drugs, susceptibility to motion sickness, anxiety
Baba [35]	2017	Japan	Cohort study	192-164-28	43.80%	15.80%	66	Esophageal cancer	Cisplatin + 5-Fluorouracil), Cisplatin + 5-Fluorouracil + Docetaxel, Cisplatin + 5-Fluorouracil + Adriamycin, Docetaxel + nedaplatin + 5-Fluorouracil, Docetaxel + nedaplatin+ S-1 (Tegafur, gimeracil, and oteracil), Nedaplatin + 5-Fluorouracil, Nedaplatin	Motion sickness, age, use of other antiemetics
Yao [36]	2017	China	Cross-sectional study	112-71-41	39.05%	31.10%	62.8	Gastric cancer, colon cancer, rectal cancer	Oxaliplatin + Fluorouracil	Pylori infection
Yokoi [18]	2018	Japan	Cohort study	156-98-58	73.00%	73.00%	63	NR (Not reported)	Cisplatin-based regimens	ERCC1 8092AA and female sex
Chai [37]	2019	China	Cohort study	643-345-298	55.20%	55.20%	56 ± 10	Respiratory system tumors, digestive system tumors, breast tumors	Cisplatin + Adriamycin + Cyclophosphamide, Oxaliplatin	Age, female sex, alcohol consumption
Meissner [38]	2019	Germany	Cohort study	121-0-121	65.00%	NR	53	Breast cancer	Platinum-based chemotherapy regimens	Age, cancer type, history of nausea, state and trait anxiety, lower quality of life
Tsuji [39]	2019	Japan	Cohort study	825-615-210	NR	NR	64	Lung cancer, esophageal cancer, stomach cancer, head and neck cancer	Cisplatin	Sex, age, cisplatin dose, granisetron use
Yoshida [40]	2019	Japan	Cohort study	312-148-164	NR	NR	NR	Digestive organ cancers, lung cancers, gynecological cancers	Oxaliplatin, Carboplatin	Elevation of serum creatinine, fatigue, performance status, elevation in alanine aminotransferase
Di Mattei [41]	2020	Italy	Cohort study	81-0-81	82.10%	NR	58	Ovarian cancer, uterine cancer, vulvar tumor	TP (Paclitaxel + Carboplatin)	Age, anticipatory nausea, patient medium-high expectations of chemotherapy-induced nausea and parity emerged
Nasu [42]	2020	Japan	Cohort study	314-141-173	43.70%	15.00%	67	NR	Carboplatin	Age and total dexamethasone dose
Simino [43]	2020	Brasil	Cohort study	269-80-188	58.00%	32.70%	55.2	Breast cancer, colorectal cancer, cervical cancer, head and neck cancer, lung cancer, esophagus cancer, gastric cancer, ovarian cancer, lymphoma, and others	Cisplatin, CDDP-P (Cisplatin + Paclitaxel), Carboplatin + Paclitaxel, Cisplatin + Fluouracil, EP	Age, tobacco use, high emetogenic chemotherapy
Takei [44]	2020	Japan	Cohort study	179-113-66	NR	NR	68	Colorectal cancer	FOLFOX (5-Fluorouracil/Leucovorin + Oxaliplatin), XELOX	BMI, female sex
Zou [45]	2020	China	Cohort study	200-116-84	NR	NR	NR	Lung cancer, intestinal cancer, breast cancer, lymphoma, cervical cancer, osteocarcinoma	GP, FOLFOX	Abdominal distension, cycles of chemotherapy, chemotherapy regimens, tumor category, physical condition, tumor metastasis
Huang [46]	2021	China	Cohort study	400-0-400	53.30%	53.30%	52.65	Breast cancer	Cisplatin	Pain/insomnia, history of CINV, chemotherapy regimens, history of motion sickness, history of CINV, chemotherapy cycles, incidence of acute CINV
Zhou [47]	2021	China	Cohort study	1000-642-468	36.00%	36.00%	56.67 ± 10.95	Lung cancer, breast cancer, aasopharyngeal carcinoma, esophageal cancer, gastric cancer, colorectal cancer, cervical cancer, ovarian cancer, lymphoma, gallbladder cancer, pancreatic cancer, soft tissue sarcoma	Oxaliplatin	Antiemetic drugs, chemotherapy cycles, age, combined radiotherapy
									Cisplatin, Carboplatin	
Shimokawa [48]	2021	Japan	Cohort study	661-391-270	37.72%	10.13%	64	Colorectal cancer	Oxaliplatin	Female sex, antiemetic drugs
Deng [49]	2022	China	Cohort study	2215-1297-918	28.80%	28.80%	55.79 ± 12.08	Lung cancer, gastrointestinal tumors, gynecological tumors, breast tumors, and others	Platinum-based chemotherapy regimens	Chemotherapy regimens, chemotherapy regimens including AC (Anthracycline + Cyclophosphamide) drugs, 1 day before chemotherapy, night sleep duration <7 h, prodromal CINV symptoms, moderate to severe anxiety before chemotherapy, pain, age, alcohol consumption, fatigue, previous history of CINV, chemotherapy psychological expectations
Sun [50]	2022	China	Cohort study	871-448-423	NR	NR	63.03 ± 10.33	Lung cancer, liver cancer, melanoma, colorectal cancer, breast cancer, gastroesophageal cancer	Platinum-based chemotherapy regimens	Men with larger body surface areas, opioids, and first chemotherapy; women with anxiety and tension
Yin [51]	2022	China	Cohort study	110--59-51	100.00%	100.00%	52.5	Gastric cancer	Oxaliplatin	Psychological state, chemotherapy cycles, NRS score and dietary quality
Ng [52]	2023	Indonesia	Cross-sectional study	137-0-137	43.10%	43.10%	52.1	Breast cancer	Cisplatin, Carboplatin	BMI
Zhang [53]	2023	China	Cohort study	320-NR-NR	40.94%	29.38%	NR	Breast cancer, lung cancer, stomach cancer, colorectal cancer, esophageal cancer, lymphoma, ovarian cancer, and others	Oxaliplatin	Age, gender, history of alcohol consumption, PSQI score, previous history of CINV, history of pregnancy and vomiting, number of chemotherapy sessions, types of antiemetic drugs, participation in social work, motion sickness, use of non-steroidal anti-inflammatory drugs, psychological expectation of CINV, anxiety
Zhao [54]	2023	China	Cohort study	720-524-182	48.70%	22.10%	NR	Lung cancer, urogenital cancer, gastrointestinal cancer, and others	Cisplatin-based chemotherapy regimens	Female cancer patients without a history of alcohol consumption, with larger BSA and received high-dose cisplatin
Ostwal [55]	2024	China	Cohort study	560-259-301	68.00%	23.00%	56	Colorectal cancer, gastric cancer, gastroesophageal cancer, non-small-cell lung cancer, carcinoma, biliary tract carcinoma, urinary bladder cancer, and others	Oxaliplatin, Carboplatin	Use of olanzapine
Wei [56]	2024	China	Cohort study	500-0-500	47.20%	47.20%	NR	Breast cancer	Platinum-based chemotherapy regimens	Anxiety, electrolyte imbalance, motion sickness history, gastrointestinal disease history, nausea and vomiting situation at 24 h before chemotherapy, chemotherapy regimens containing anthracycline or platinum, multidrug combination

**Abbreviation:** GP (Gemcitabine + Cisplatin), DP (Docetaxel + Cisplatin), TP (Paclitaxel + Cisplatin/Carboplatin), NP (Vincristine + Cisplatin), EP (Etoposide + Cisplatin), XELOX (Capecitabine + Oxaliplatin), EN (Etoposide + Cisplatin), S-1 (Tegafur + Gimeracil + Oteracil), CDDP-P (Cisplatin + Paclitaxel), FOLFOX (5-Fluorouracil/Leucovorin + Oxaliplatin), and NR (Not reported).

## Data Availability

The original contributions presented in the study are included in the article/Supplementary Materials; further inquiries can be directed to the corresponding authors.

## Supplementary Materials

The following supporting information can be downloaded at: https://www.mdpi.com/article/10.3390/curroncol32060325/s1.

## Abbreviations

The following abbreviations are used in this manuscript:

GP	Gemcitabine + Cisplatin
PINV	Platinum-based chemotherapy-induced nausea and vomiting
CINV	Chemotherapy-induced nausea and vomiting
CNKI	China National Knowledge Infrastructure
CMAJD	Chinese Medical Association Journal Database
VIP	China Science and Technology Journal Database
DP	Docetaxel + Cisplatin
TP	Paclitaxel + Cisplatin/carboplatin
NP	Vincristine + Cisplatin
EP	Etoposide + Cisplatin
XELOX	Capecitabine + Oxaliplatin
EN	Etoposide + Cisplatin
S-1	Tegafur + Gimeracil + Oteracil
CDDP-P	Cisplatin + Paclitaxel
FOLFOX	5-Fluorouracil/leucovorin + Oxaliplatin
NR	Not reported
MASCC	Multinational Association of Supportive Care in Cancer
RCT	randomized controlled trial
