# Supplementary material for: Incidence and Risk Factors of Platinum-Based Chemotherapy-Induced Nausea and Vomiting: A Systematic Review and Meta-Analysis
Source: Curr Oncol. 2025 May 31;32(6):325. doi: 10.3390/curroncol32060325 (PMC12191659; doi:10.3390/curroncol32060325)
Supplement: Supplementary file 1 [file curroncol-32-00325-s001.zip › curroncol-3636150-supplementary.pdf]

Incidence Rate and Risk Factors platinum-based chemotherapy induced Nausea and Vomiting Induced: A Systematic Review and Meta-analysis

Table S1 Search strategy

Database	Search Results	After Screening	Search Query	Search Date
Wanfang	434	37	(危险因素 [Risk factors] OR 影响因素 [influencing factors] OR 相关因素 [related factors] OR 预测因素 [predictive factors] OR 患病 [illness] OR 发病 [onset] OR 发生 [occurrence] OR 高危 [high-risk]) AND (化疗恶心呕吐 [chemotherapy-induced nausea and vomiting]).	January 20, 2025
CNKI	638	24	(危险因素 [Risk factors] OR 影响因素 [influencing factors] OR 相关因素 [related factors] OR 预测因素 [predictive factors] OR 患病 [illness] OR 发病 [onset] OR 发生 [occurrence] OR 高危 [high-risk]) AND (化疗恶心呕吐 [chemotherapy-induced nausea and vomiting]).	January 20, 2025
PubMed	1292	79	(risk factors OR factors OR prevalence OR incidence OR relative risk OR predict) AND (chemotherapy-induced nausea OR chemotherapy-induced vomiting OR PINV)	January 20, 2025

Embase	1528	37	(risk factors OR factors OR prevalence OR incidence OR relative risk OR predict) AND (chemotherapy-induced nausea OR chemotherapy-induced vomiting OR PINV)	January 20, 2025
Web of Science	2576	82	(risk factors OR factors OR prevalence OR incidence OR relative risk OR predict) AND (chemotherapy-induced nausea OR chemotherapy-induced vomiting OR PINV)	January 20, 2025

Table S2 Quality assessment

Table S2.1 Risk of bias of the included studies was assessed using the Joanna Briggs Institute Critical Appraisal tool for cross-sectional studies.

First author(year)	1.Were the criteria for inclusion in the sample clearly defined?	2.Were the study subjects and the setting described in detail?	3.Was the exposure measured in a valid and reliable way?	4.Were objective, standard criteria used for measurement of the condition?	5.Were confounding factors identified?	6.Were strategies to deal with confounding factors stated?	7.Were the outcomes measured in a valid and reliable way?	8.Was appropriate statistical analysis used?	Overall appraisal
Liu(2011)	yes	yes	yes	yes	yes	yes	yes	yes	A
Zhang(2011)	yes	yes	yes	yes	yes	yes	yes	yes	A
Wei(2012)	yes	yes	yes	yes	yes	yes	yes	yes	A
lv(2015)	yes	yes	yes	yes	yes	yes	yes	yes	A
Yao(2017)	yes	yes	yes	yes	yes	no	no	yes	A
Ng(2023)	yes	yes	yes	yes	no	no	yes	yes	B

Each of item can be assessed as “yes”, “no”, “unclear”, and “not applicable” . The quality level of the research that is satisfactory for all items is A, and the quality level of the research that is satisfactory for some items is B (items 1-3 are "not")

Table S2.2 Quality assessment of the cohort studies was performed using the Newcastle-Ottawa Scale (NOS) Quality Assessment Tool.

	Study population selection				Comparability between groups	Measurement of exposure factors			
First author(year)	Representativeness of the Exposed Cohort(1 point)	Selection of the Non-Exposed Cohort(1 point)	Ascertainment of Exposure(1 point)	Demonstration That Outcome of Interest Was Not Present at Start of Study(1 point)	Comparability of Cohorts on the Basis of the Design or Analysis(2 points)	Assessment of Outcome(1 point)	Was Follow-Up Long Enough for Outcomes to Occur(1 point)	Adequacy of Follow Up of Cohorts(1 point)	Overall appraisal
Chan(2012)	1	1	1	1	2	1	0	0	7
Hilarius(2012)	1	1	1	1	2	1	1	1	9
Poon(2013)	1	1	1	1	2	1	1	1	9
Hu(2016)	1	1	1	0	2	1	1	0	7
Mukoyama(2016)	1	1	1	1	2	1	1	1	9
Baba(2017)	1	1	1	1	1	1	1	0	7
Yokoi(2018)	1	0	1	1	1	1	1	0	6
Meissner(2019)	1	1	1	1	2	1	1	0	8
Tsuji(2019)	1	1	1	1	2	1	1	0	8
Yoshida(2019)	1	1	1	1	2	1	1	1	9

**Chai(2019)**	**1**	**1**	**1**	**1**	**2**	**1**	**0**	**0**	**7**
**Di Mattei(2020)**	**1**	**1**	**1**	**1**	**2**	**1**	**1**	**0**	**8**
**Nasu(2020)**	**1**	**1**	**1**	**1**	**1**	**1**	**1**	**0**	**7**
**Simino(2020)**	**1**	**1**	**1**	**1**	**2**	**1**	**1**	**1**	**9**
**Takei(2020)**	**1**	**1**	**1**	**1**	**2**	**1**	**1**	**0**	**8**
**Chu(2020)**	**1**	**1**	**1**	**1**	**1**	**1**	**0**	**0**	**6**
**Huang(2021)**	**1**	**1**	**1**	**1**	**2**	**1**	**1**	**0**	**8**
**Zhou(2021)**	**1**	**1**	**1**	**1**	**2**	**1**	**1**	**1**	**9**
**Shimokawa(2021)**	**1**	**1**	**1**	**0**	**1**	**1**	**1**	**1**	**7**
**Den(2022)**	**1**	**1**	**1**	**1**	**2**	**1**	**1**	**1**	**9**
**Sun(2022)**	**1**	**1**	**1**	**1**	**2**	**1**	**1**	**1**	**9**
**Yin(2022)**	**1**	**1**	**1**	**1**	**1**	**1**	**0**	**0**	**6**
**Zhao(2023)**	**1**	**0**	**1**	**1**	**2**	**1**	**1**	**1**	**8**
**Zhang(2023)**	**1**	**0**	**1**	**1**	**2**	**1**	**0**	**1**	**8**
**Ostwal(2024)**	**1**	**1**	**1**	**1**	**2**	**1**	**1**	**1**	**9**
**Wei(2024)**	**1**	**1**	**1**	**1**	**1**	**1**	**0**	**1**	**7**

A perfect score is 9 points; 0 to 4 indicates a low-quality study, and 5 to 9 indicates a high-quality study.

Table S3 Meta-Analysis of Risk Factors Associated with PINV

Risk Factors	Number of Studies	Heterogeneity		EffectModel	The Results of the Meta-Analysis		
		I^2	P		OR (95% CI)	Z	p
Age	18	88.30%	0	Random	1.048(0.978-1.123)	1.324	0.186
Male	4	66.20%	0.031	Random	0.688(0.460-1.028)	-1.824	0.068
Female	10	87.80%	0	Random	2.363(1.363-4.096)	3.065	0.002
BMI	5	63.10%	0.028	Random	1.235(0.702-2.170)	0.733	0.464
Alcohol consumption	12	52.40%	0.017	Random	0.846(0.677-1.058)	-1.466	0.143
Smoking	3	0.00%	0.903	Fixed	1.220(0.828-1.795)	1.006	0.314
Anxiety	6	95.50%	0	Random	1.689(1.057-2.700)	2.192	0.028
Fatigue	3	35.20%	0.214	Fixed	1.413(1.145-1.744)	3.226	0.001
Performance status	3	67.10%	0.048	Random	1.338(0.581-3.078)	0.684	0.494
Motion sickness	8	50.40%	0.049	Random	1.816(1.266-2.605)	3.241	0.001
Courses of chemotherapy	4	94.40%	0	Random	1.677(0.443-6.349)	0.761	0.446

Number of chemotherapy sessions	3	92.50%	0	Random	0.963(0.749-1.238)	-0.297	0.767
History of vomiting during chemotherapy	4	88.00%	0	Random	2.728(1.468-5.069)	3.174	0.002
Chemotherapy regimen	7	85.50%	0	Random	1.29(0.839-1.985)	1.159	0.246
Prechemo therapy nausea	4	75.00%	0.007	Random	1.708(0.887-3.290)	1.600	0.110
Expectation of nausea	3	73.20%	0.024	Random	1.859(0.793-4.358)	1.472	0.154
Antiemetic regimen	5	90.20%	0	Random	1.106(0.490-2.496)	0.243	0.808

Meta-analysis results Supplementary material

Incidence of nausea

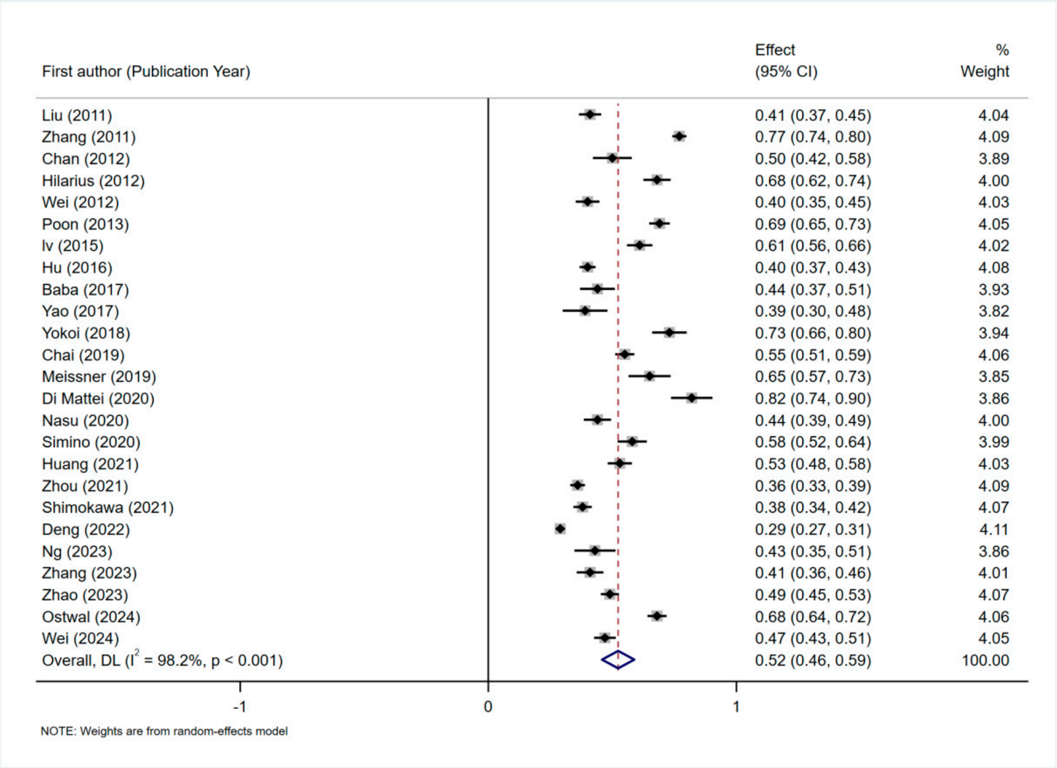

FigureS 1Forest Plot of the Incidence of Nausea in platinum-based Chemotherapy Patients

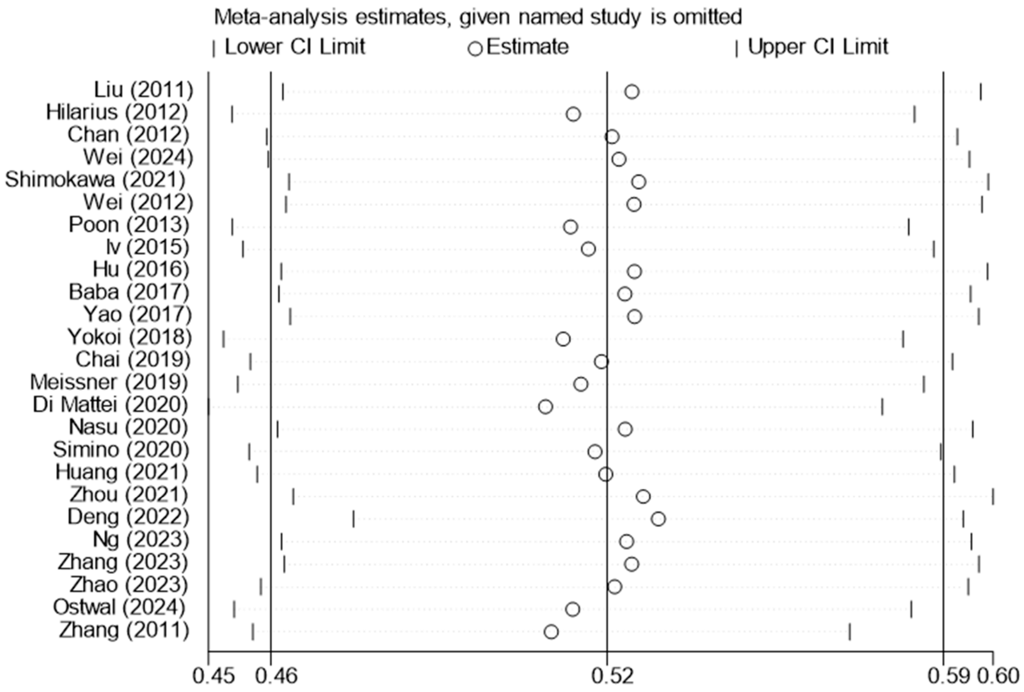

FigureS 2Sensitivity Analysis of the Incidence of Nausea in platinum-based Chemotherapy Patients

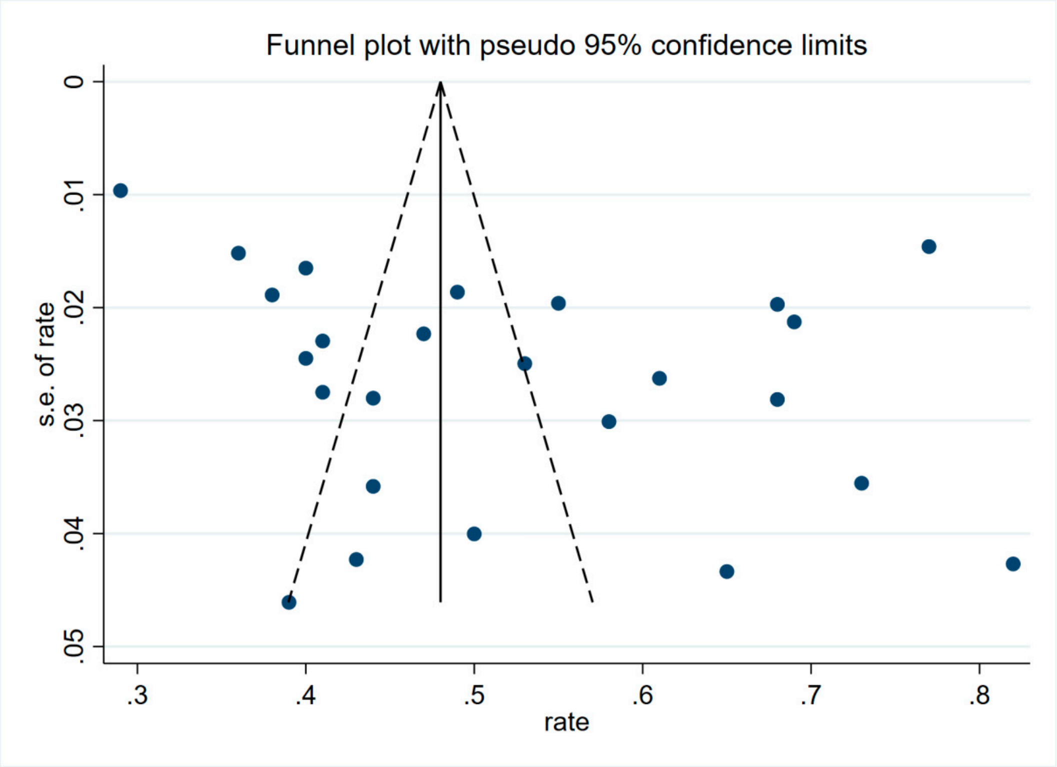

FigureS 3Funnel Plot of Publication Bias in Studies Reporting the Incidence of Nausea in platinum-based Chemotherapy Patients

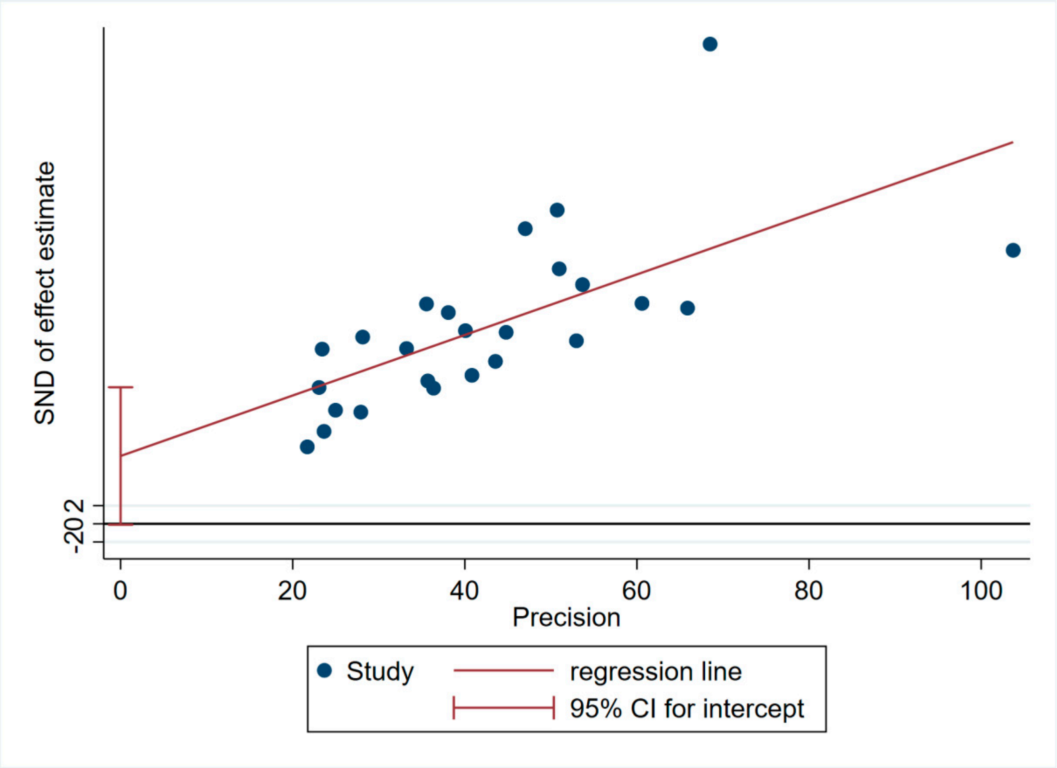

FigureS 4 Egger’ s Test of Publication Bias for Studies Assessing the Incidence of Nausea in platinum-based Chemotherapy Patients

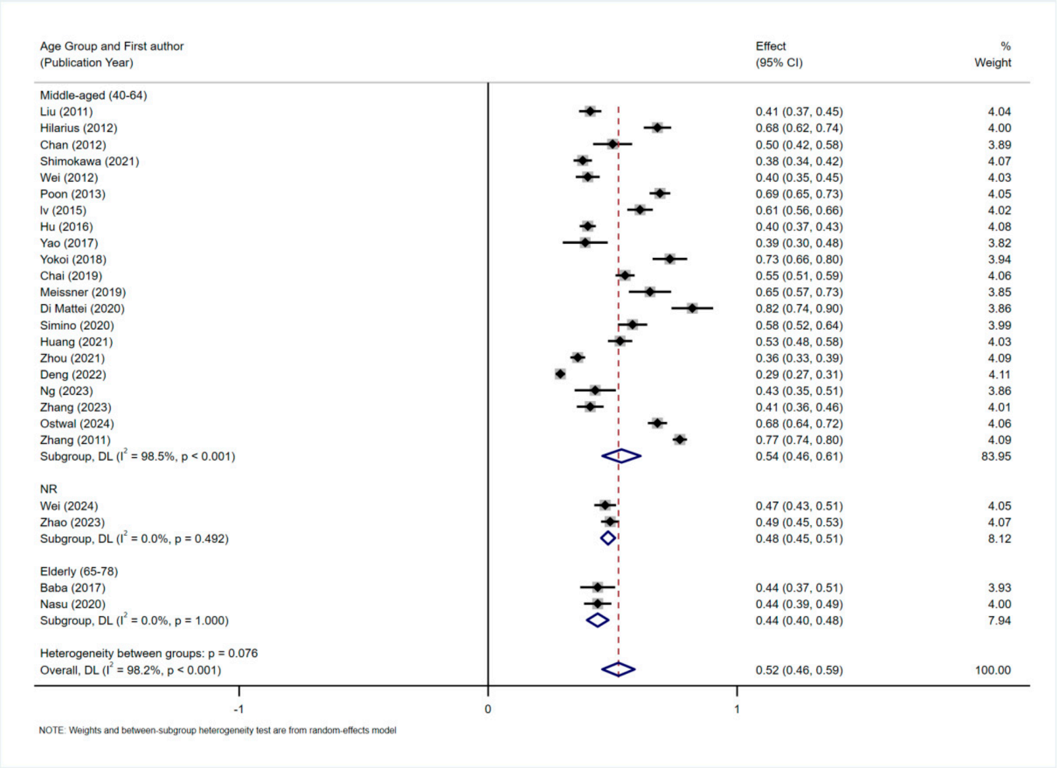

FigureS 5 Forest Plot of Subgroup Analysis by Age Group for the Incidence of Nausea in platinum-based Chemotherapy Patients

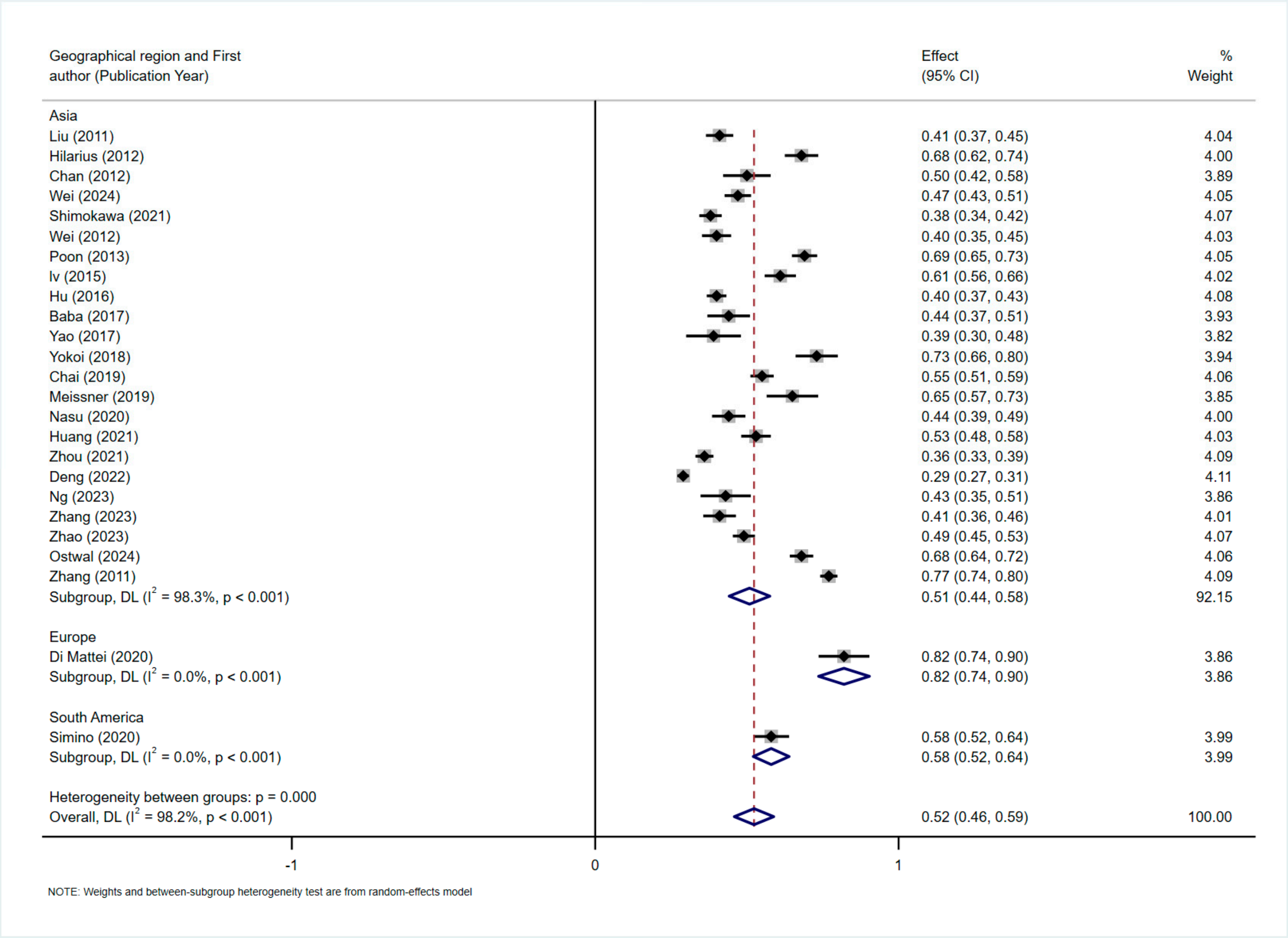
Geographical region and First author (Publication Year)
Effect (95% CI)
% Weight
Asia
Liu (2011) 0.41 (0.37, 0.45) 4.04
Hilarius (2012) 0.68 (0.62, 0.74) 4.00
Chan (2012) 0.50 (0.42, 0.58) 3.89
Wei (2024) 0.47 (0.43, 0.51) 4.05
Shimokawa (2021) 0.38 (0.34, 0.42) 4.07
Wei (2012) 0.40 (0.35, 0.45) 4.03
Poon (2013) 0.69 (0.65, 0.73) 4.05
lv (2015) 0.61 (0.56, 0.66) 4.02
Hu (2016) 0.40 (0.37, 0.43) 4.08
Baba (2017) 0.44 (0.37, 0.51) 3.93
Yao (2017) 0.39 (0.30, 0.48) 3.82
Yokoi (2018) 0.73 (0.66, 0.80) 3.94
Chai (2019) 0.55 (0.51, 0.59) 4.06
Meissner (2019) 0.65 (0.57, 0.73) 3.85
Nasu (2020) 0.44 (0.39, 0.49) 4.00
Huang (2021) 0.53 (0.48, 0.58) 4.03
Zhou (2021) 0.36 (0.33, 0.39) 4.09
Deng (2022) 0.29 (0.27, 0.31) 4.11
Ng (2023) 0.43 (0.35, 0.51) 3.86
Zhang (2023) 0.41 (0.36, 0.46) 4.01
Zhao (2023) 0.49 (0.45, 0.53) 4.07
Ostwal (2024) 0.68 (0.64, 0.72) 4.06
Zhang (2011) 0.77 (0.74, 0.80) 4.09
Subgroup, DL (I^2 = 98.3%, p < 0.001) 0.51 (0.44, 0.58) 92.15
Europe
Di Mattei (2020) 0.82 (0.74, 0.90) 3.86
Subgroup, DL (I^2 = 0.0%, p < 0.001) 0.82 (0.74, 0.90) 3.86
South America
Simino (2020) 0.58 (0.52, 0.64) 3.99
Subgroup, DL (I^2 = 0.0%, p < 0.001) 0.58 (0.52, 0.64) 3.99
Heterogeneity between groups: p = 0.000
Overall, DL (I^2 = 98.2%, p < 0.001) 0.52 (0.46, 0.59) 100.00
-1
0
1
NOTE: Weights and between-subgroup heterogeneity test are from random-effects model

FigureS 6　Forest Plot of Subgroup Analysis by Geographical Region for the Incidence of Nausea in platinum-based Chemotherapy Patients

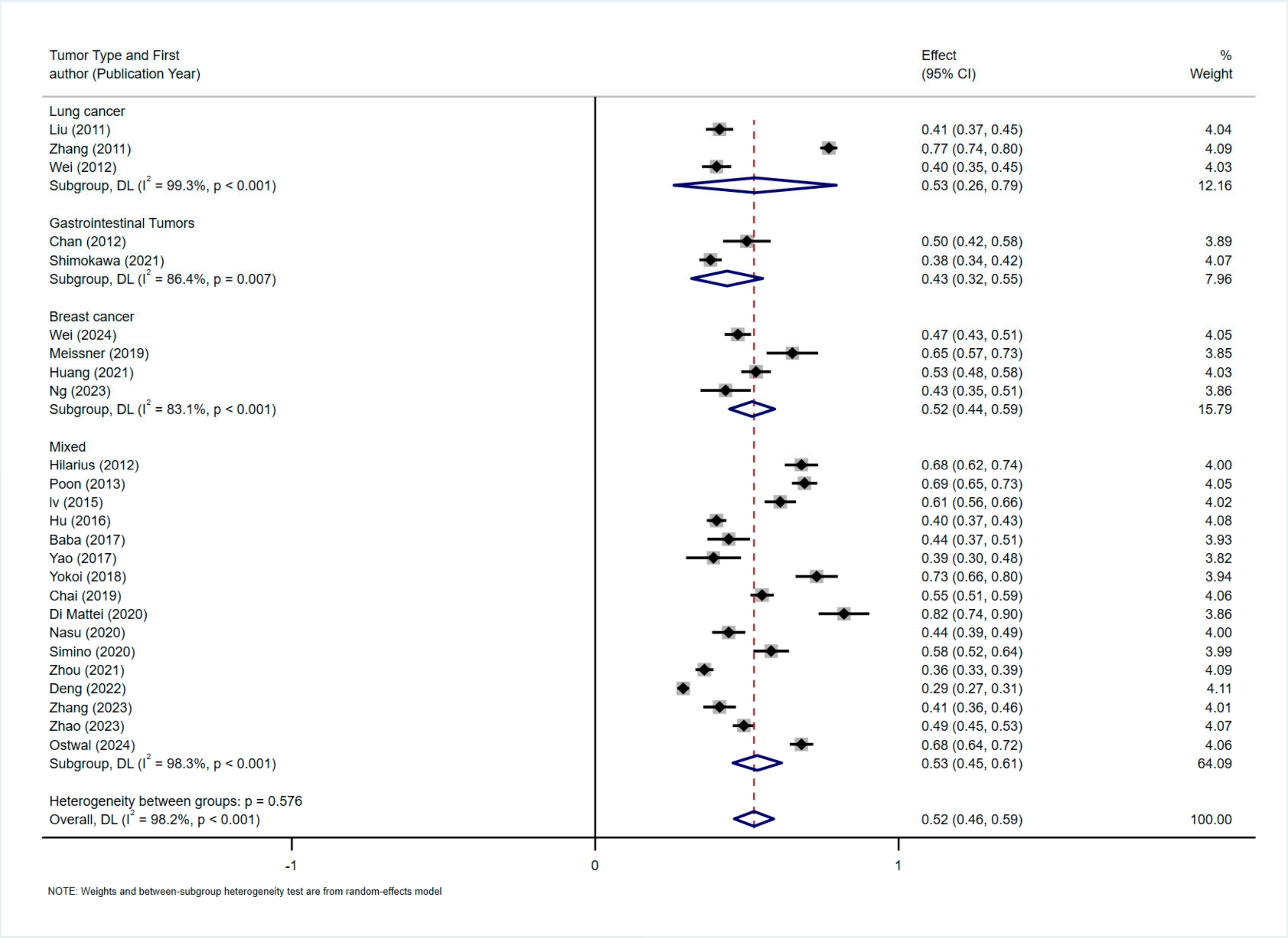
Tumor Type and First
author (Publication Year)
Effect
(95% CI)
%
Weight
Lung cancer
Liu (2011) 0.41 (0.37, 0.45) 4.04
Zhang (2011) 0.77 (0.74, 0.80) 4.09
Wei (2012) 0.40 (0.35, 0.45) 4.03
Subgroup, DL (I^2 = 99.3%, p < 0.001) 0.53 (0.26, 0.79) 12.16
Gastrointestinal Tumors
Chan (2012) 0.50 (0.42, 0.58) 3.89
Shimokawa (2021) 0.38 (0.34, 0.42) 4.07
Subgroup, DL (I^2 = 86.4%, p = 0.007) 0.43 (0.32, 0.55) 7.96
Breast cancer
Wei (2024) 0.47 (0.43, 0.51) 4.05
Meissner (2019) 0.65 (0.57, 0.73) 3.85
Huang (2021) 0.53 (0.48, 0.58) 4.03
Ng (2023) 0.43 (0.35, 0.51) 3.86
Subgroup, DL (I^2 = 83.1%, p < 0.001) 0.52 (0.44, 0.59) 15.79
Mixed
Hilarius (2012) 0.68 (0.62, 0.74) 4.00
Poon (2013) 0.69 (0.65, 0.73) 4.05
lv (2015) 0.61 (0.56, 0.66) 4.02
Hu (2016) 0.40 (0.37, 0.43) 4.08
Baba (2017) 0.44 (0.37, 0.51) 3.93
Yao (2017) 0.39 (0.30, 0.48) 3.82
Yokoi (2018) 0.73 (0.66, 0.80) 3.94
Chai (2019) 0.55 (0.51, 0.59) 4.06
Di Mattei (2020) 0.82 (0.74, 0.90) 3.86
Nasu (2020) 0.44 (0.39, 0.49) 4.00
Simino (2020) 0.58 (0.52, 0.64) 3.99
Zhou (2021) 0.36 (0.33, 0.39) 4.09
Deng (2022) 0.29 (0.27, 0.31) 4.11
Zhang (2023) 0.41 (0.36, 0.46) 4.01
Zhao (2023) 0.49 (0.45, 0.53) 4.07
Ostwal (2024) 0.68 (0.64, 0.72) 4.06
Subgroup, DL (I^2 = 98.3%, p < 0.001) 0.53 (0.45, 0.61) 64.09
Heterogeneity between groups: p = 0.576
Overall, DL (I^2 = 98.2%, p < 0.001) 0.52 (0.46, 0.59) 100.00
-1
0
1
NOTE: Weights and between-subgroup heterogeneity test are from random-effects model

FigureS 7 Forest Plot of Subgroup Analysis by Tumor Type for the Incidence of Nausea in platinum-based Chemotherapy Patients

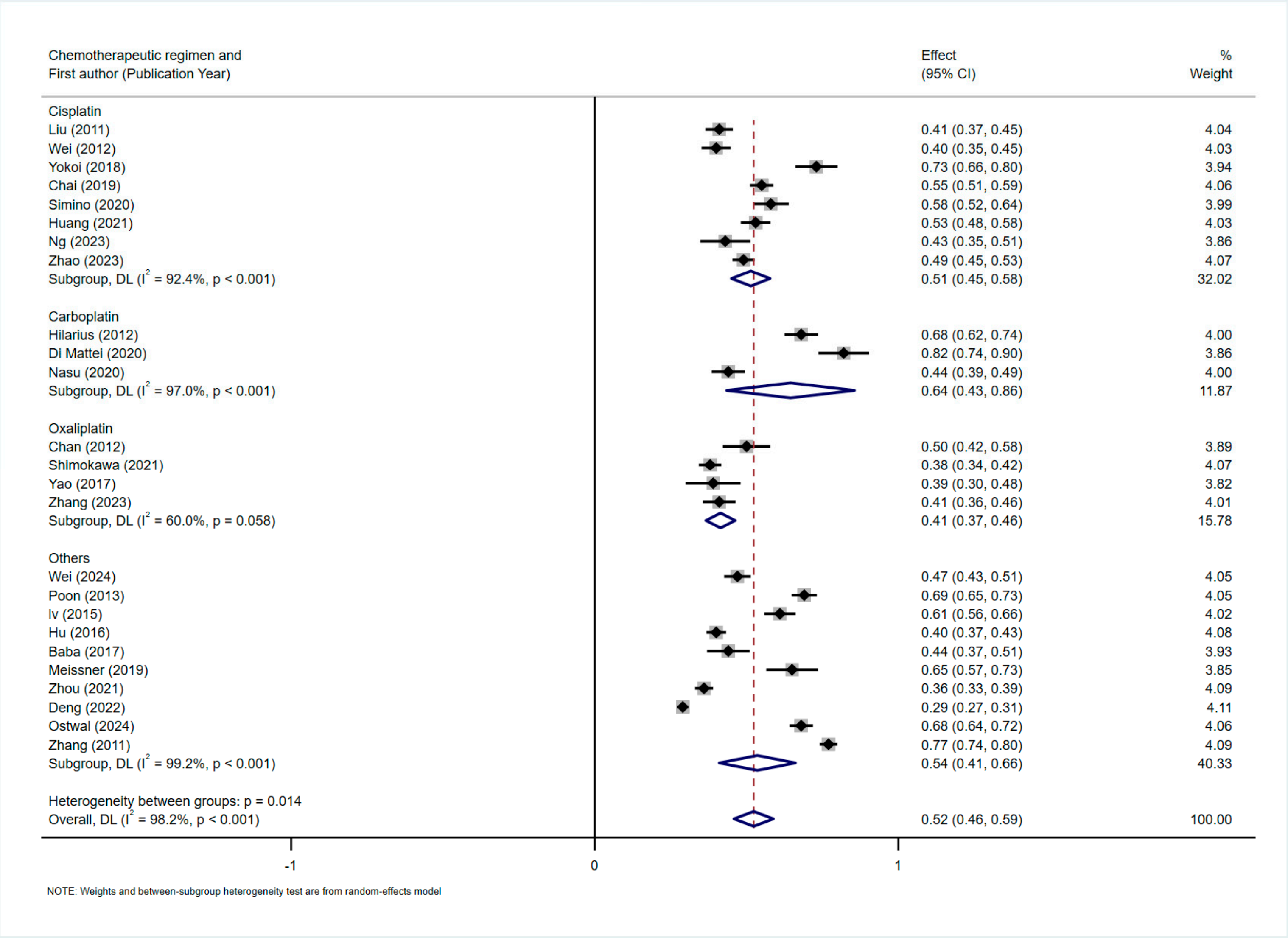
Chemotherapeutic regimen and
First author (Publication Year)
Effect
(95% CI)
%
Weight
Cisplatin
Liu (2011) 0.41 (0.37, 0.45) 4.04
Wei (2012) 0.40 (0.35, 0.45) 4.03
Yokoi (2018) 0.73 (0.66, 0.80) 3.94
Chai (2019) 0.55 (0.51, 0.59) 4.06
Simino (2020) 0.58 (0.52, 0.64) 3.99
Huang (2021) 0.53 (0.48, 0.58) 4.03
Ng (2023) 0.43 (0.35, 0.51) 3.86
Zhao (2023) 0.49 (0.45, 0.53) 4.07
Subgroup, DL (I^2 = 92.4%, p < 0.001) 0.51 (0.45, 0.58) 32.02
Carboplatin
Hilarius (2012) 0.68 (0.62, 0.74) 4.00
Di Mattei (2020) 0.82 (0.74, 0.90) 3.86
Nasu (2020) 0.44 (0.39, 0.49) 4.00
Subgroup, DL (I^2 = 97.0%, p < 0.001) 0.64 (0.43, 0.86) 11.87
Oxaliplatin
Chan (2012) 0.50 (0.42, 0.58) 3.89
Shimokawa (2021) 0.38 (0.34, 0.42) 4.07
Yao (2017) 0.39 (0.30, 0.48) 3.82
Zhang (2023) 0.41 (0.36, 0.46) 4.01
Subgroup, DL (I^2 = 60.0%, p = 0.058) 0.41 (0.37, 0.46) 15.78
Others
Wei (2024) 0.47 (0.43, 0.51) 4.05
Poon (2013) 0.69 (0.65, 0.73) 4.05
lv (2015) 0.61 (0.56, 0.66) 4.02
Hu (2016) 0.40 (0.37, 0.43) 4.08
Baba (2017) 0.44 (0.37, 0.51) 3.93
Meissner (2019) 0.65 (0.57, 0.73) 3.85
Zhou (2021) 0.36 (0.33, 0.39) 4.09
Deng (2022) 0.29 (0.27, 0.31) 4.11
Ostwal (2024) 0.68 (0.64, 0.72) 4.06
Zhang (2011) 0.77 (0.74, 0.80) 4.09
Subgroup, DL (I^2 = 99.2%, p < 0.001) 0.54 (0.41, 0.66) 40.33
Heterogeneity between groups: p = 0.014
Overall, DL (I^2 = 98.2%, p < 0.001) 0.52 (0.46, 0.59) 100.00
-1
0
1
NOTE: Weights and between-subgroup heterogeneity test are from random-effects model

FigureS 8 Forest Plot of Subgroup Analysis by Chemotherapeutic regimen for the Incidence of Nausea in platinum-based Chemotherapy Patients

Sources of Heterogeneity：

Despite extensive efforts, including sensitivity analysis, meta-regression, and subgroup analysis, the source of heterogeneity remained unexplained, potentially due to unmeasured confounders or random variation.

Incidence of Vomiting

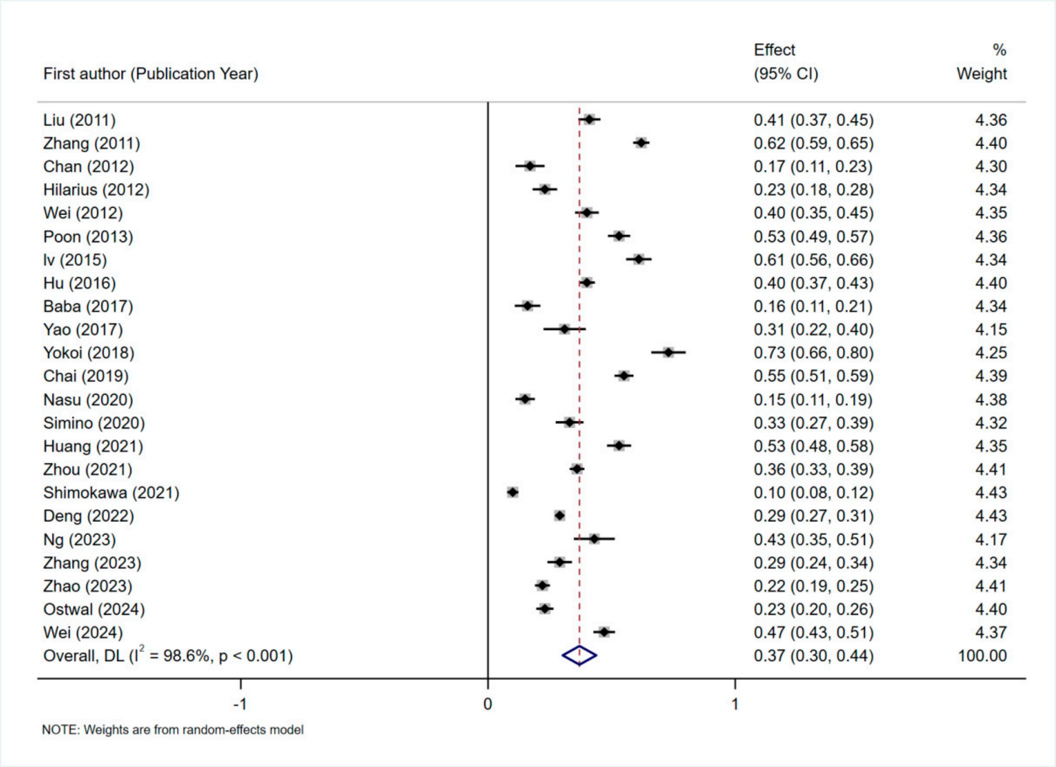

FigureS 9Forest Plot of the Incidence of Vomiting in platinum-based Chemotherapy Patients

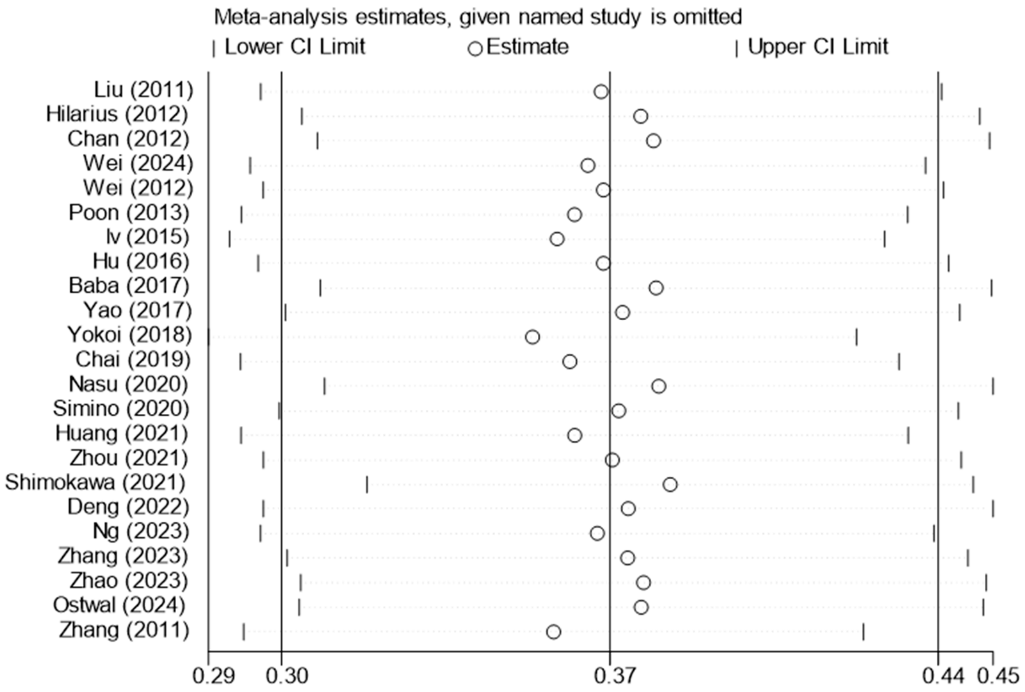

FigureS 10 Sensitivity Analysis of the Incidence of Vomiting in platinum-based Chemotherapy Patients

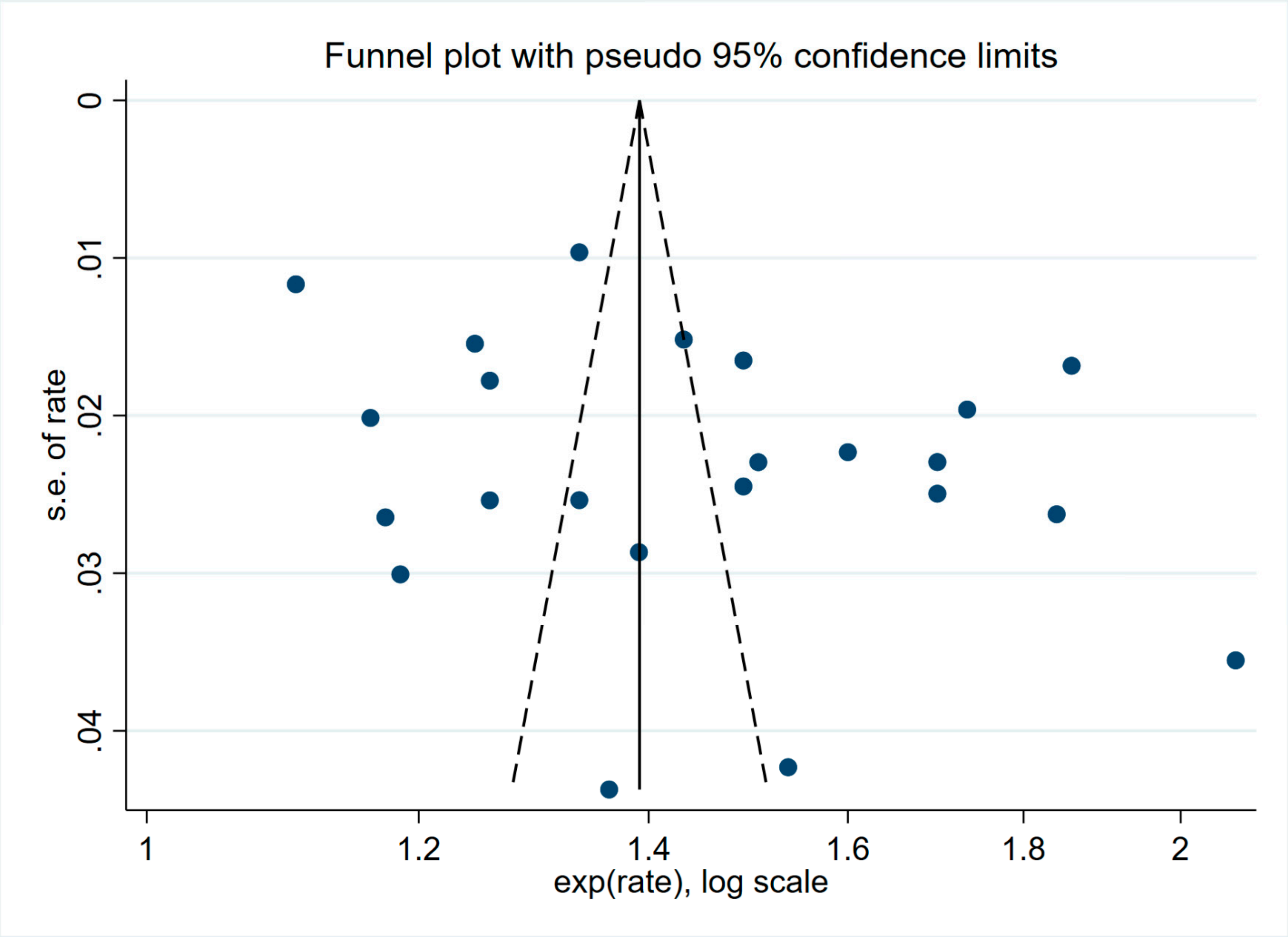

Funnel plot with pseudo 95% confidence limits
s.e. of rate
0
.01
.02
.03
.04
1
1.2
1.4
1.6
1.8
2
exp(rate), log scale

FigureS 11Funnel Plot of Publication Bias in Studies Reporting the Incidence of Nausea in platinum-based Chemotherapy Patients

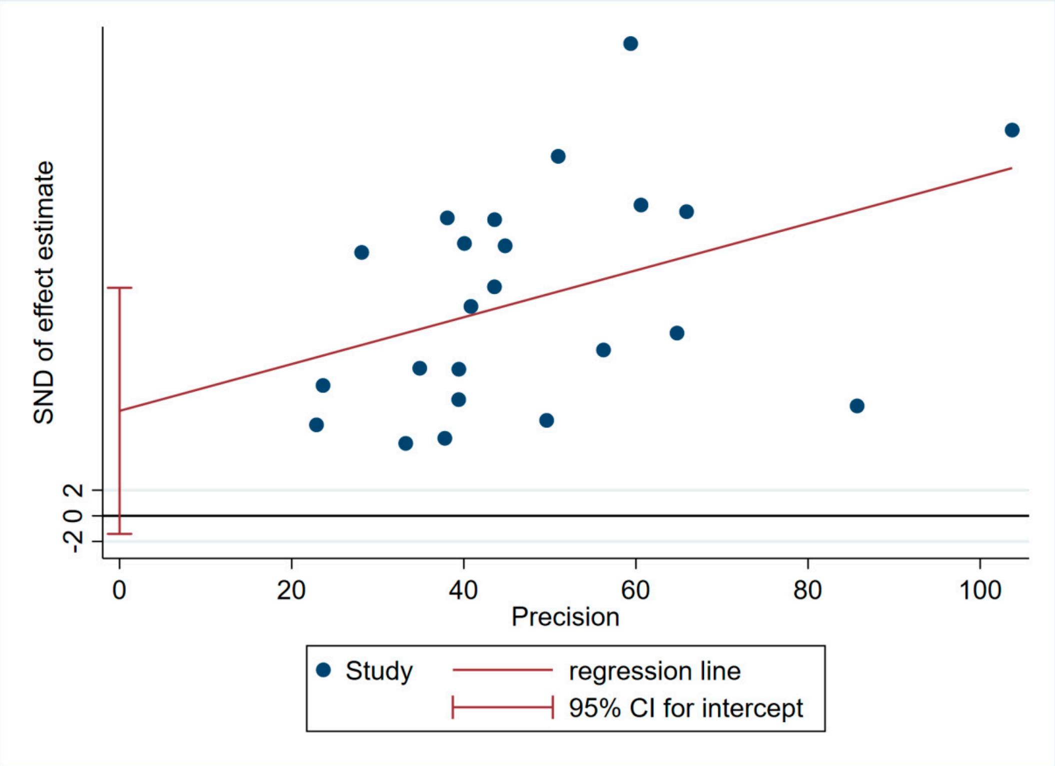

FigureS 12Egger’ s Test of Publication Bias for Studies Assessing the Incidence of Vomiting in platinum-based Chemotherapy Patients

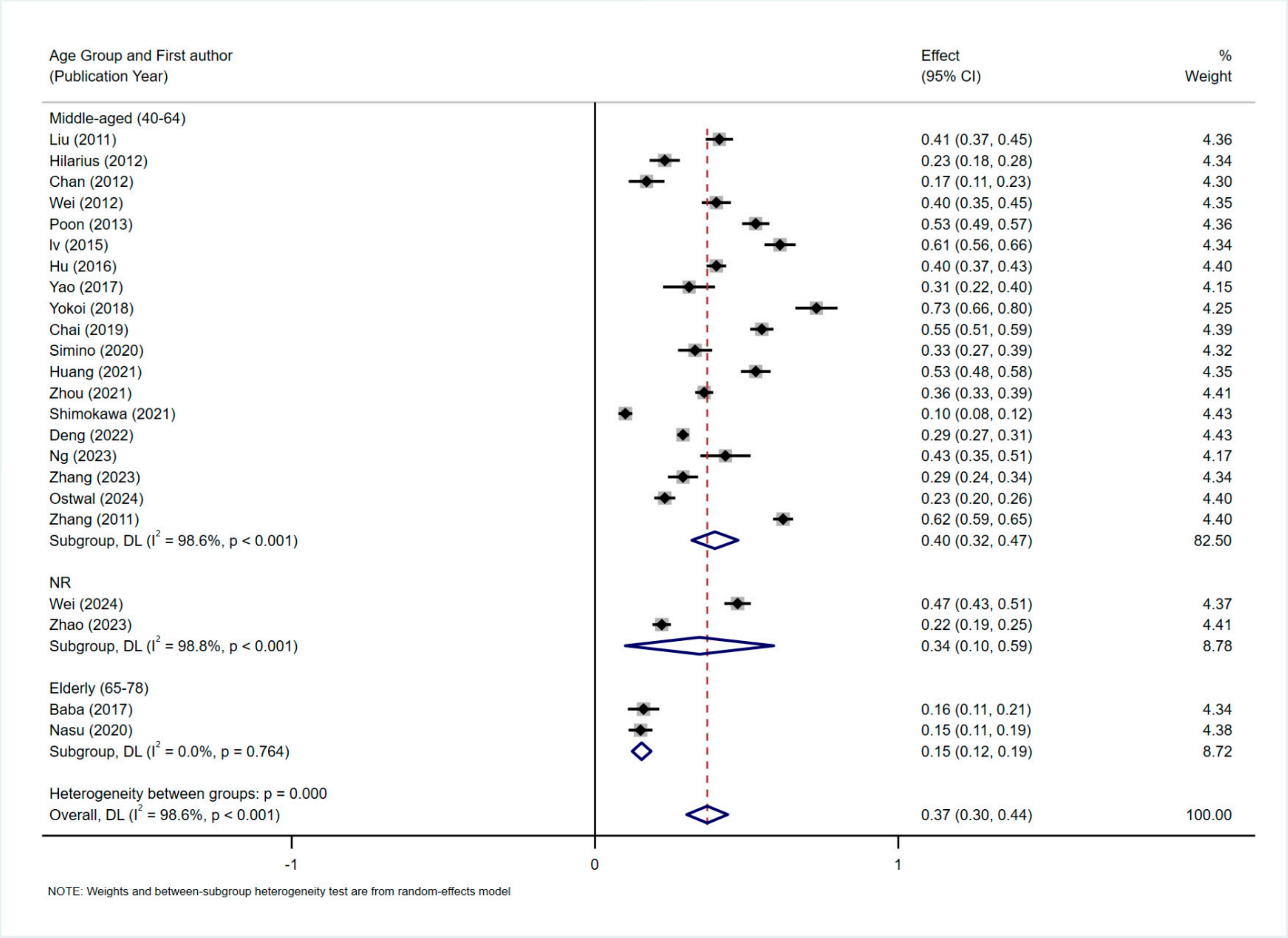
Age Group and First author
(Publication Year)
Effect
(95% CI)
%
Weight
Middle-aged (40-64)
Liu (2011) 0.41 (0.37, 0.45) 4.36
Hilarius (2012) 0.23 (0.18, 0.28) 4.34
Chan (2012) 0.17 (0.11, 0.23) 4.30
Wei (2012) 0.40 (0.35, 0.45) 4.35
Poon (2013) 0.53 (0.49, 0.57) 4.36
lv (2015) 0.61 (0.56, 0.66) 4.34
Hu (2016) 0.40 (0.37, 0.43) 4.40
Yao (2017) 0.31 (0.22, 0.40) 4.15
Yokoi (2018) 0.73 (0.66, 0.80) 4.25
Chai (2019) 0.55 (0.51, 0.59) 4.39
Simino (2020) 0.33 (0.27, 0.39) 4.32
Huang (2021) 0.53 (0.48, 0.58) 4.35
Zhou (2021) 0.36 (0.33, 0.39) 4.41
Shimokawa (2021) 0.10 (0.08, 0.12) 4.43
Deng (2022) 0.29 (0.27, 0.31) 4.43
Ng (2023) 0.43 (0.35, 0.51) 4.17
Zhang (2023) 0.29 (0.24, 0.34) 4.34
Ostwal (2024) 0.23 (0.20, 0.26) 4.40
Zhang (2011) 0.62 (0.59, 0.65) 4.40
Subgroup, DL (I^2 = 98.6%, p < 0.001) 0.40 (0.32, 0.47) 82.50
NR
Wei (2024) 0.47 (0.43, 0.51) 4.37
Zhao (2023) 0.22 (0.19, 0.25) 4.41
Subgroup, DL (I^2 = 98.8%, p < 0.001) 0.34 (0.10, 0.59) 8.78
Elderly (65-78)
Baba (2017) 0.16 (0.11, 0.21) 4.34
Nasu (2020) 0.15 (0.11, 0.19) 4.38
Subgroup, DL (I^2 = 0.0%, p = 0.764) 0.15 (0.12, 0.19) 8.72
Heterogeneity between groups: p = 0.000
Overall, DL (I^2 = 98.6%, p < 0.001) 0.37 (0.30, 0.44) 100.00
-1
0
1
NOTE: Weights and between-subgroup heterogeneity test are from random-effects model

FigureS 13Forest Plot of Subgroup Analysis by Age Group for the Incidence of Vomiting in platinum-based Chemotherapy Patients

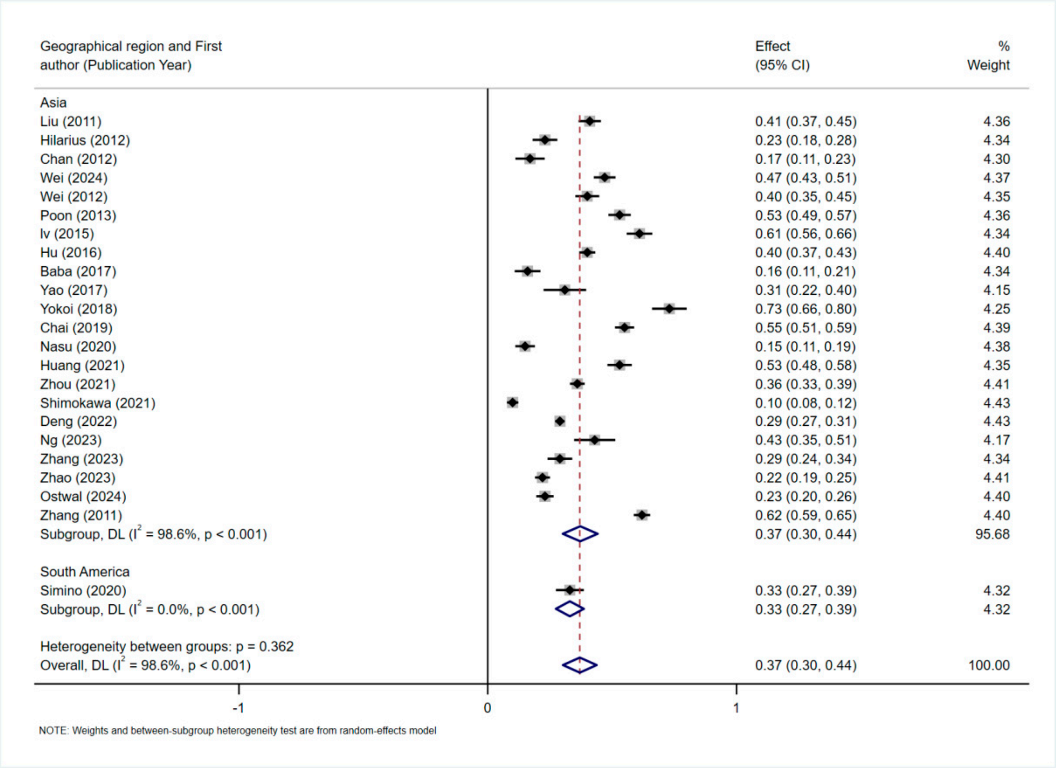

FigureS 14Forest Plot of Subgroup Analysis by Geographical Region for the Incidence of Vomiting in platinum-based Chemotherapy Patients

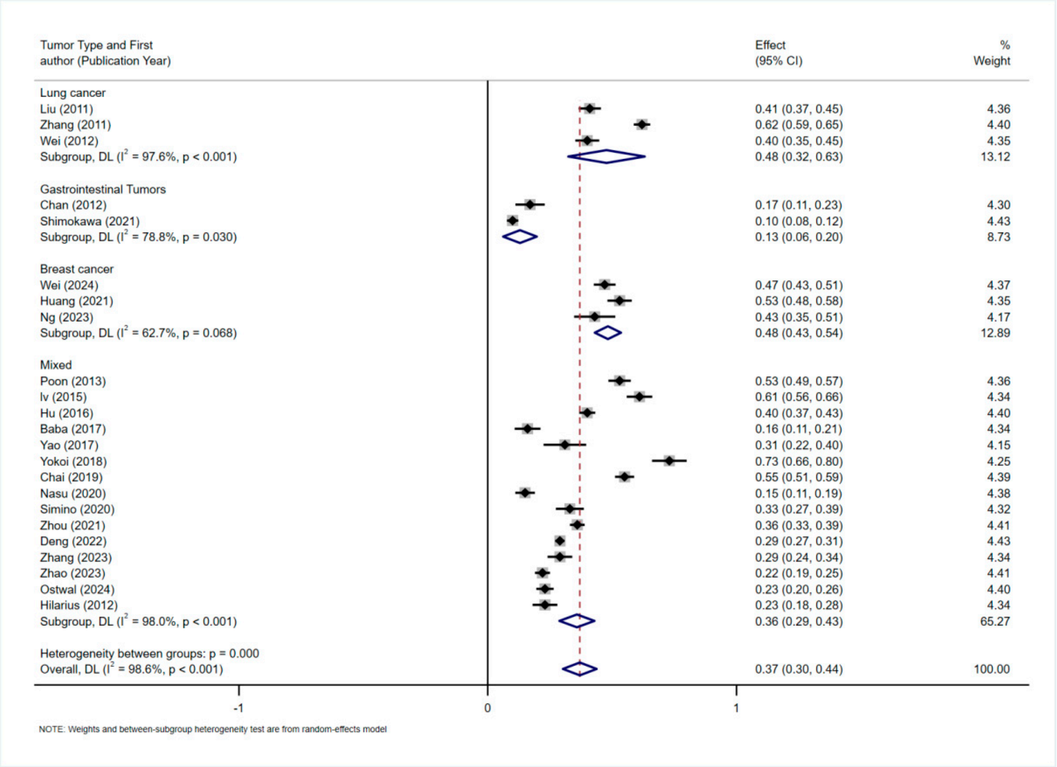

FigureS 15 Forest Plot of Subgroup Analysis by Tumor Type for the Incidence of Vomiting in platinum-based Chemotherapy Patients

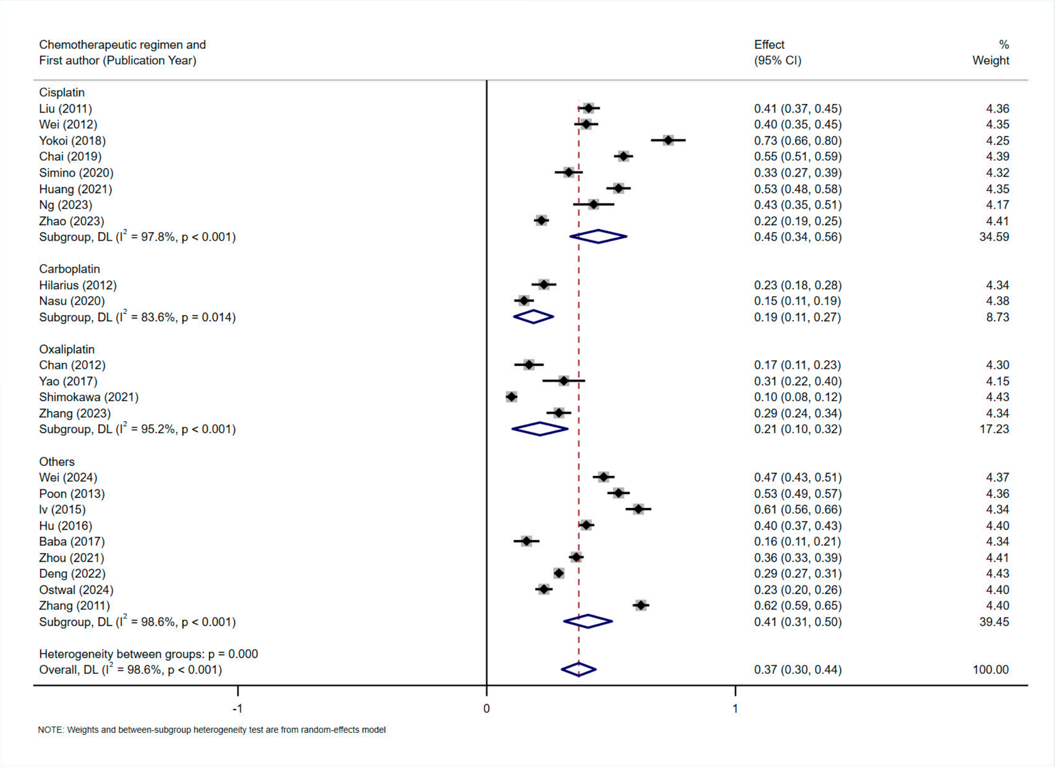

FigureS 16 Forest Plot of Subgroup Analysis by Chemotherapeutic regimen for the Incidence of Vomiting in platinum-based Chemotherapy Patients

Sources of Heterogeneity:

Despite extensive efforts, including sensitivity analysis, meta-regression, and subgroup analysis, the source of heterogeneity remained unexplained, potentially due to unmeasured confounders or random variation.

Age

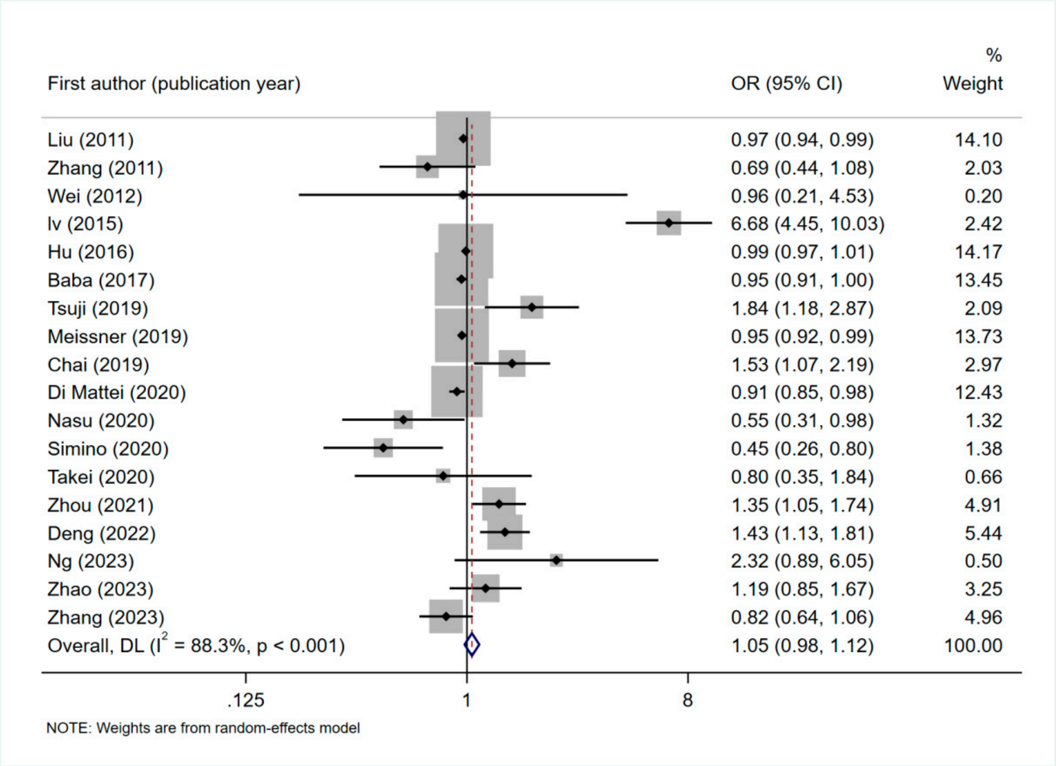

FigureS 17 Forest Plot of the Association Between Age and PINV

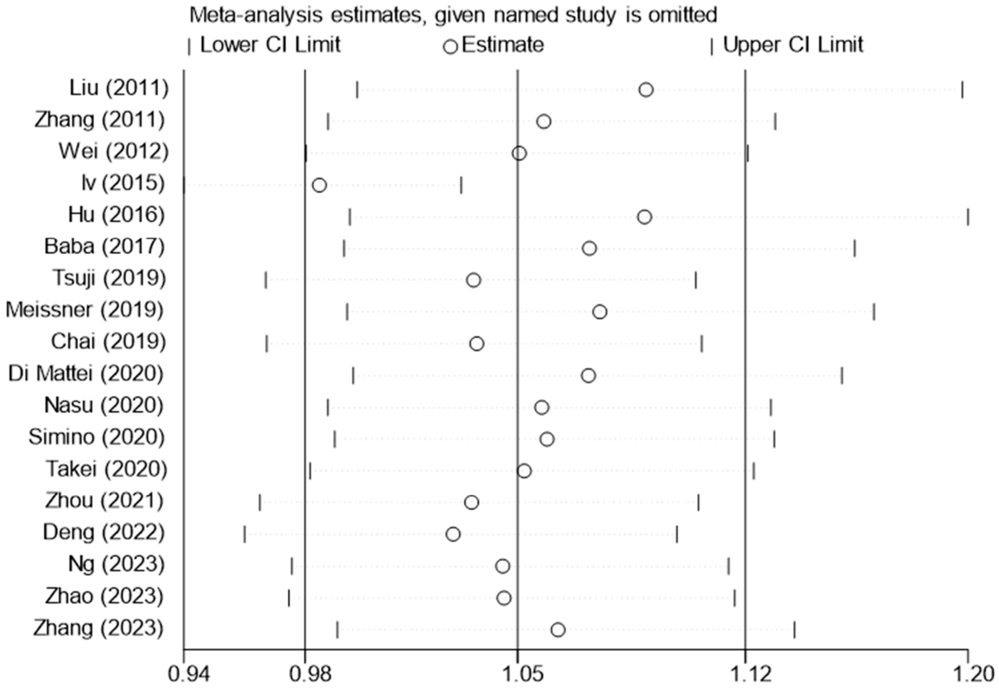

FigureS 18Sensitivity Analysis of the Association Between Age and PINV

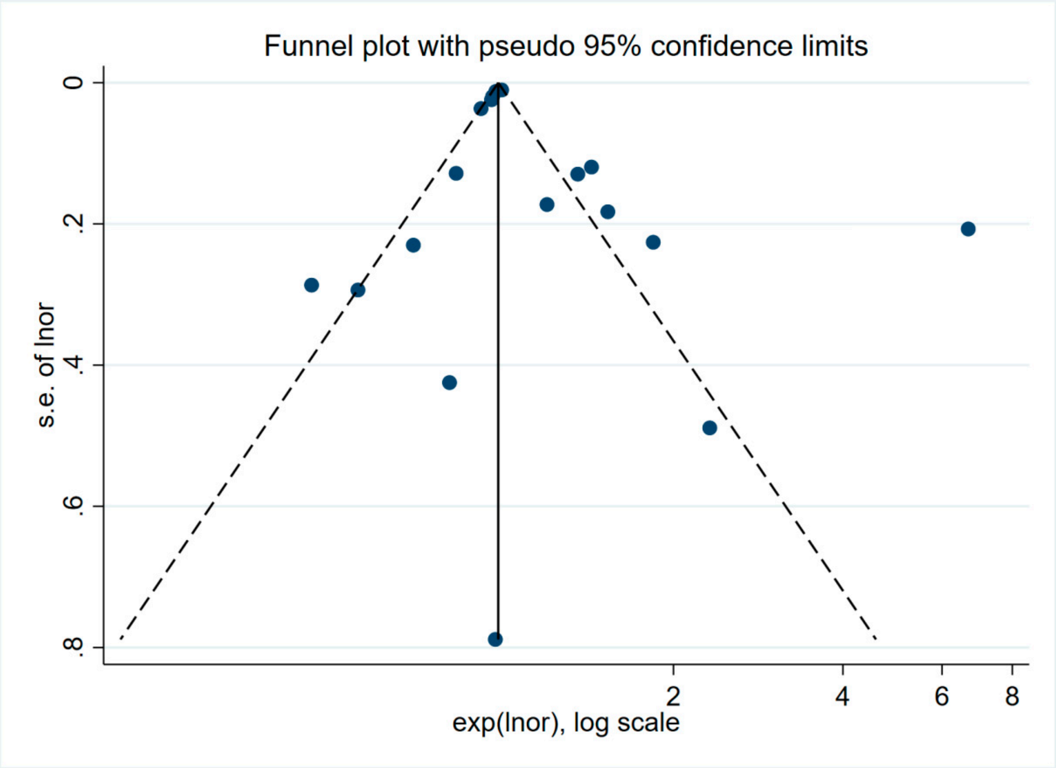

FigureS 19Funnel Plot for Assessing Publication Bias in Studies on Age as a Risk Factor for PINV

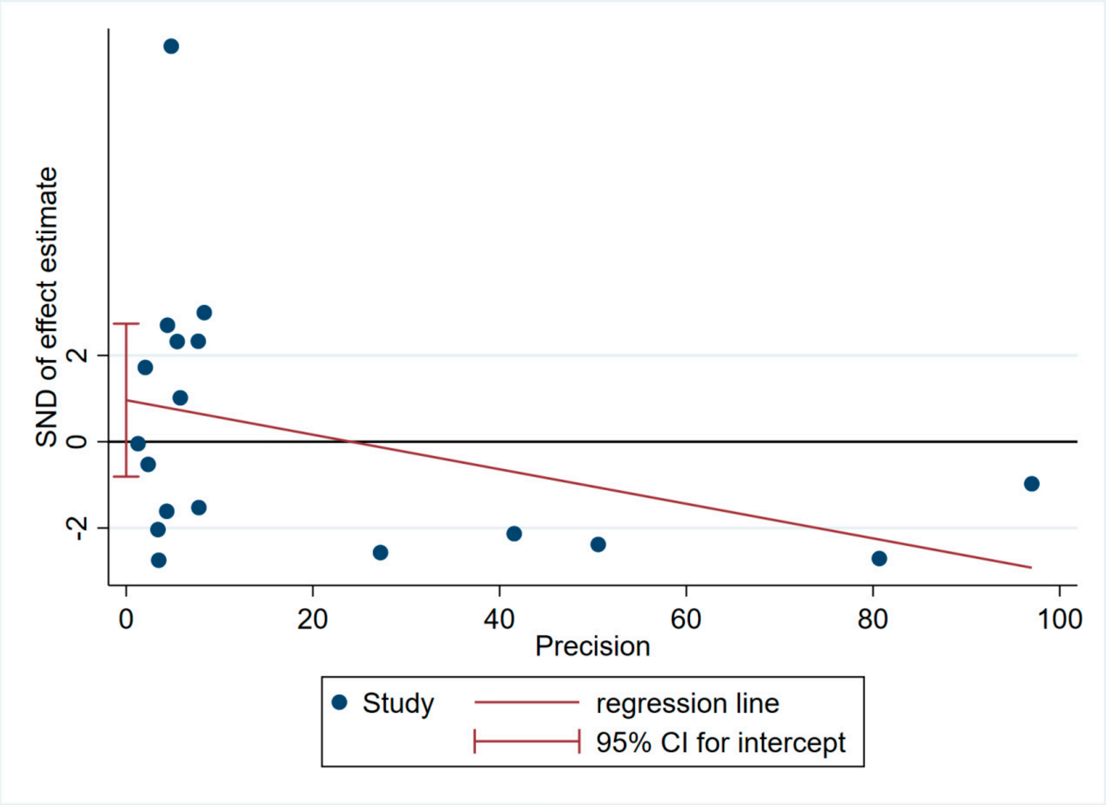

FigureS 20Egger's Regression Test to Detect Publication Bias in the Association Between Age and PINV

[illegible]

```
Meta-regression                                    Number of obs  =       18
REML estimate of between-study variance            tau2           =    .2461
% residual variation due to heterogeneity          I-squared_res  =   89.05%
Proportion of between-study variance explained     Adj R-squared  =   14.77%
Joint test for all covariates                      Model F(2,15)  =     2.32
With Knapp-Hartung modification                    Prob > F       =   0.1322
```

lnor	Coefficient	Std. err.	t	P>|t|	[95% conf.	interval]
studydesign	.379158	.3117489	1.22	0.243	-.2853191	1.043635
geographicalregion	-.2721644	.1852672	-1.47	0.162	-.667052	.1227233
_cons	.0030774	.5105591	0.01	0.995	-1.085154	1.091308

FigureS 21Meta-Regression Analysis of the Association Between Age and PINV

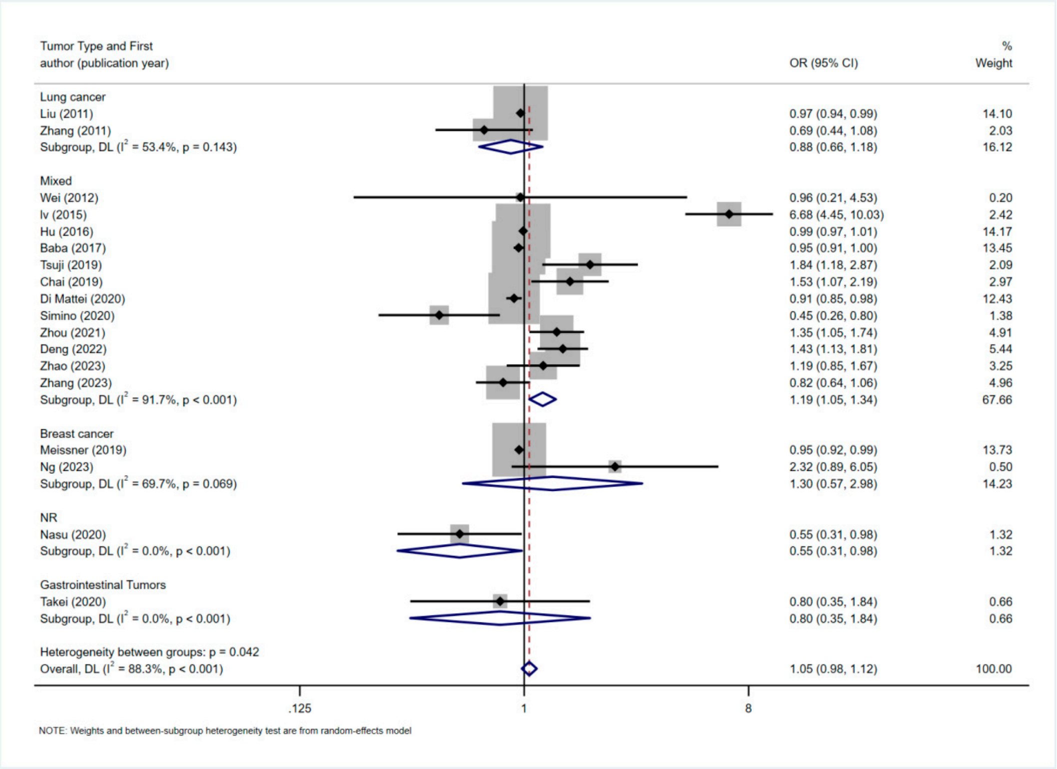

FigureS 22 Forest Plot of Subgroup Analysis by Tumor Type for the Association Between Age and PINV

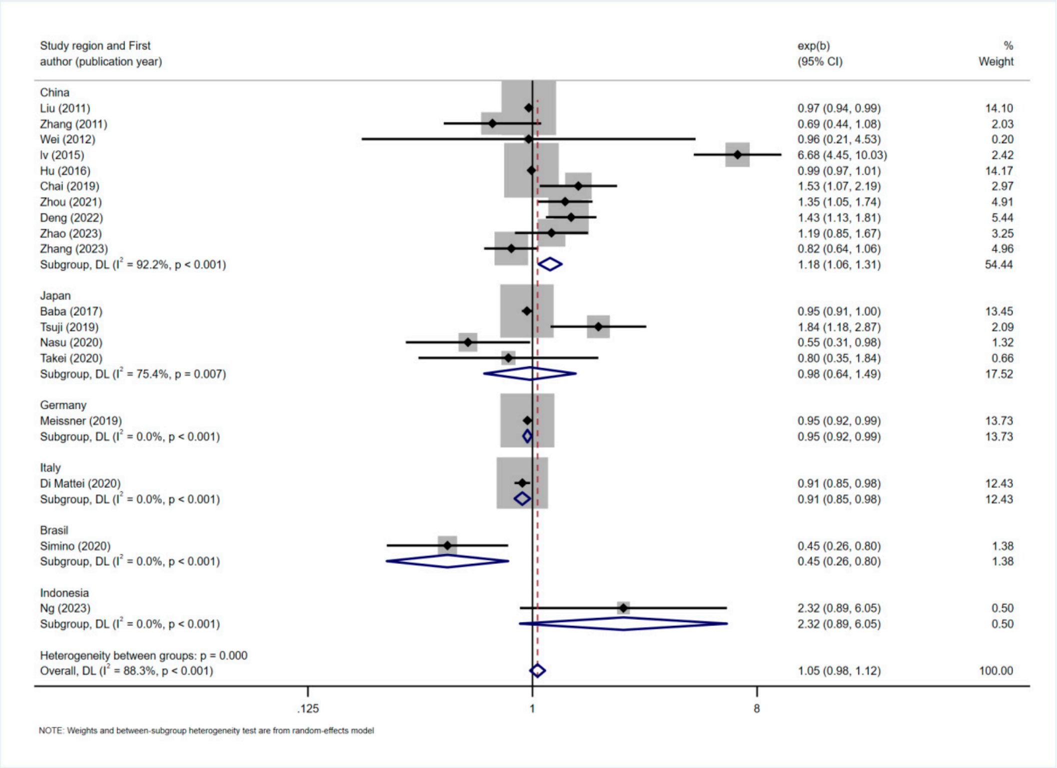

FigureS 23Forest Plot of Subgroup Analysis by Geographic Region for the Association Between Age and PINV

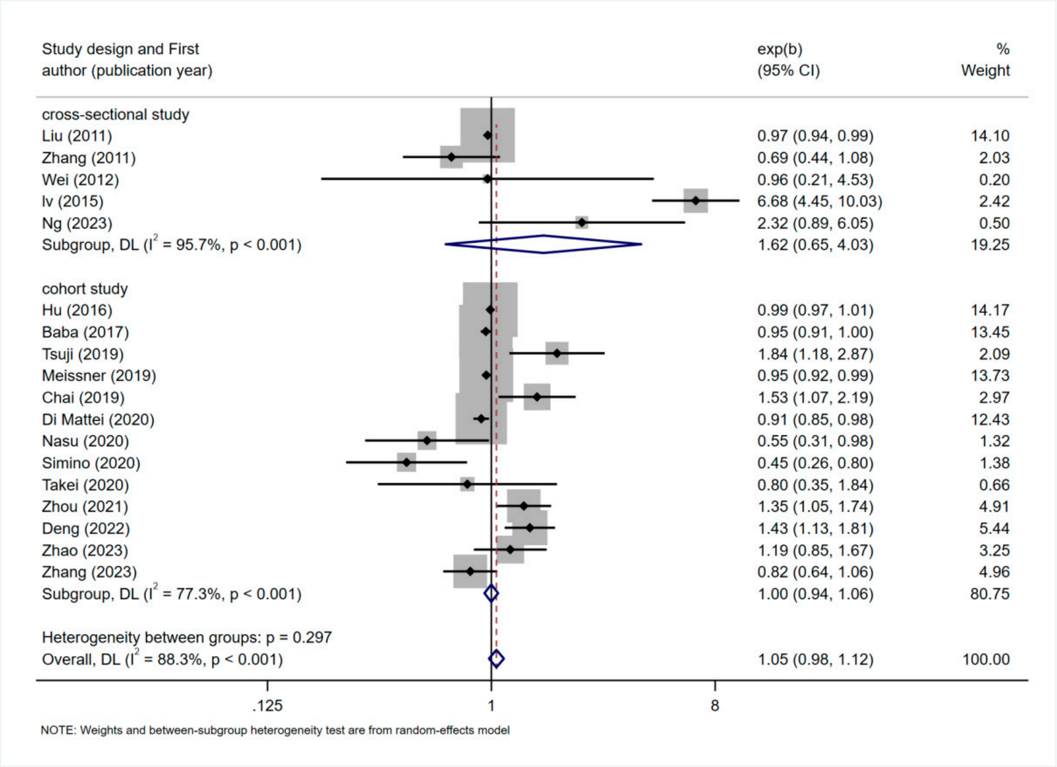

FigureS 24Forest Plot of Subgroup Analysis by Study Design for the Association Between Age and PINV

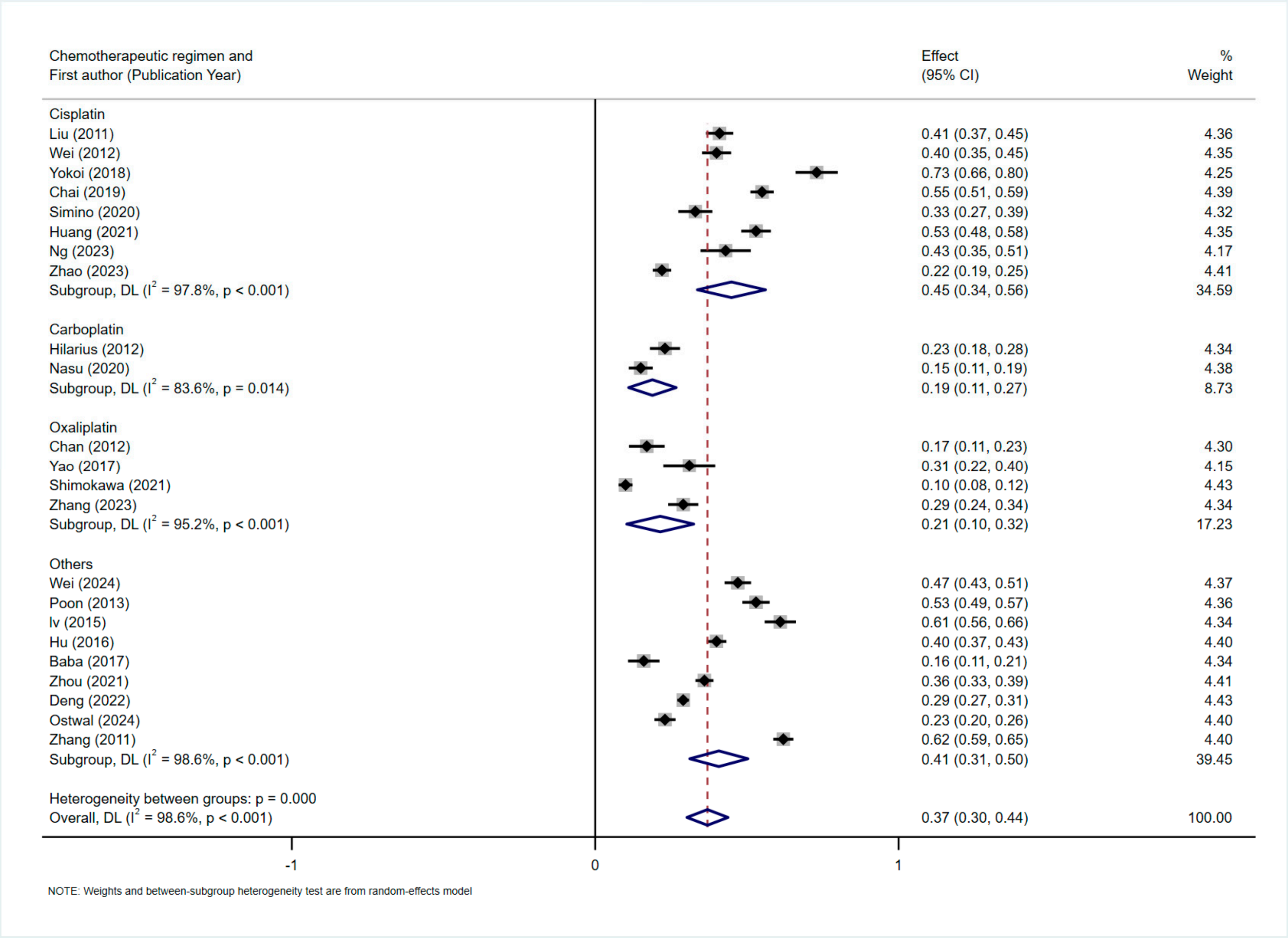
Chemotherapeutic regimen and
First author (Publication Year)
Effect
(95% CI)
%
Weight
Cisplatin
Liu (2011) 0.41 (0.37, 0.45) 4.36
Wei (2012) 0.40 (0.35, 0.45) 4.35
Yokoi (2018) 0.73 (0.66, 0.80) 4.25
Chai (2019) 0.55 (0.51, 0.59) 4.39
Simino (2020) 0.33 (0.27, 0.39) 4.32
Huang (2021) 0.53 (0.48, 0.58) 4.35
Ng (2023) 0.43 (0.35, 0.51) 4.17
Zhao (2023) 0.22 (0.19, 0.25) 4.41
Subgroup, DL (I^2 = 97.8%, p < 0.001) 0.45 (0.34, 0.56) 34.59
Carboplatin
Hilarius (2012) 0.23 (0.18, 0.28) 4.34
Nasu (2020) 0.15 (0.11, 0.19) 4.38
Subgroup, DL (I^2 = 83.6%, p = 0.014) 0.19 (0.11, 0.27) 8.73
Oxaliplatin
Chan (2012) 0.17 (0.11, 0.23) 4.30
Yao (2017) 0.31 (0.22, 0.40) 4.15
Shimokawa (2021) 0.10 (0.08, 0.12) 4.43
Zhang (2023) 0.29 (0.24, 0.34) 4.34
Subgroup, DL (I^2 = 95.2%, p < 0.001) 0.21 (0.10, 0.32) 17.23
Others
Wei (2024) 0.47 (0.43, 0.51) 4.37
Poon (2013) 0.53 (0.49, 0.57) 4.36
lv (2015) 0.61 (0.56, 0.66) 4.34
Hu (2016) 0.40 (0.37, 0.43) 4.40
Baba (2017) 0.16 (0.11, 0.21) 4.34
Zhou (2021) 0.36 (0.33, 0.39) 4.41
Deng (2022) 0.29 (0.27, 0.31) 4.43
Ostwal (2024) 0.23 (0.20, 0.26) 4.40
Zhang (2011) 0.62 (0.59, 0.65) 4.40
Subgroup, DL (I^2 = 98.6%, p < 0.001) 0.41 (0.31, 0.50) 39.45
Heterogeneity between groups: p = 0.000
Overall, DL (I^2 = 98.6%, p < 0.001) 0.37 (0.30, 0.44) 100.00
-1
0
1
NOTE: Weights and between-subgroup heterogeneity test are from random-effects model

FigureS 25Forest Plot of Subgroup Analysis by Chemotherapeutic regimen for the Association Between Age and PINV

Male

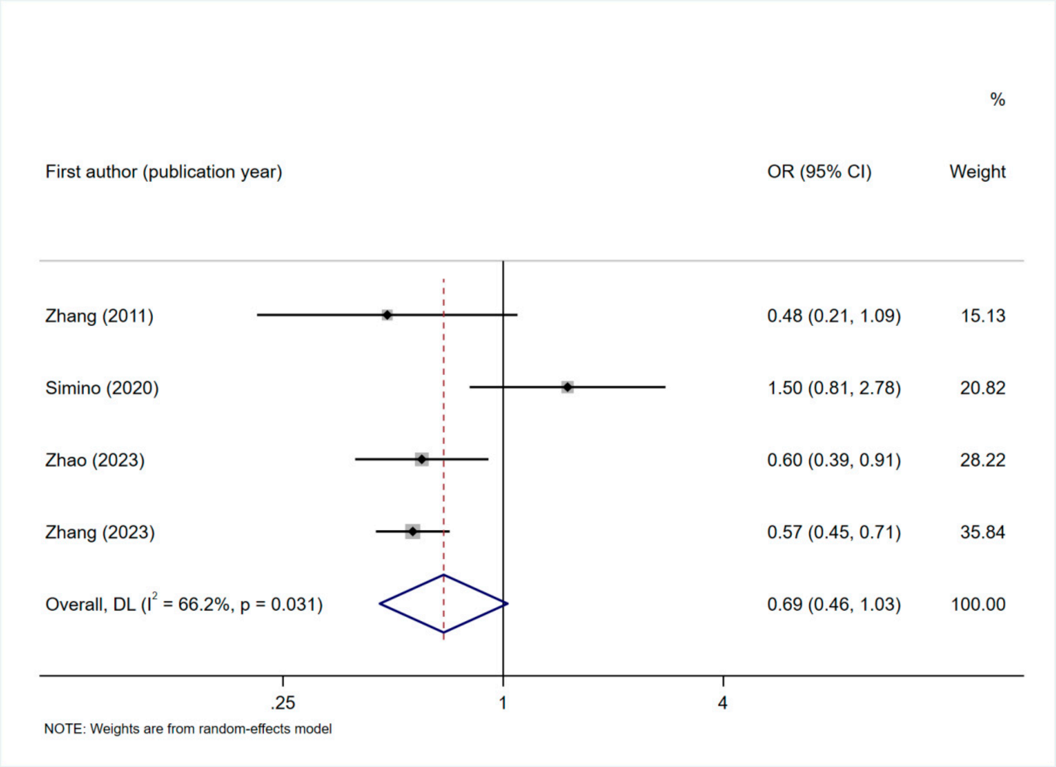

FigureS 26Forest Plot of the Association Between Male Gender and PINV

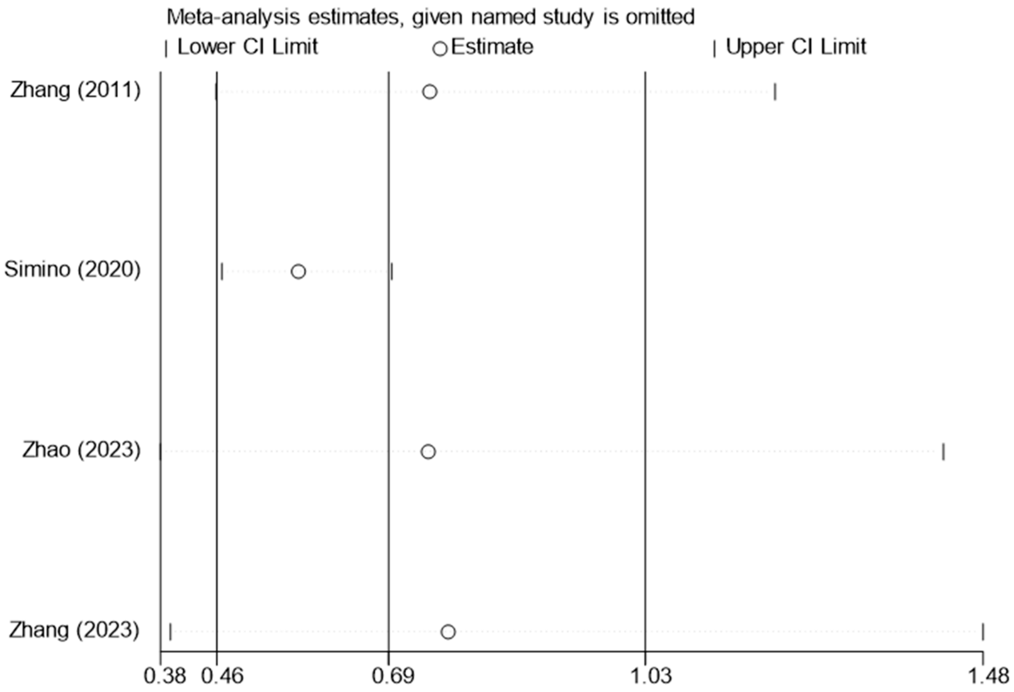

FigureS 27Sensitivity Analysis of the Association Between Male Gender and PINV

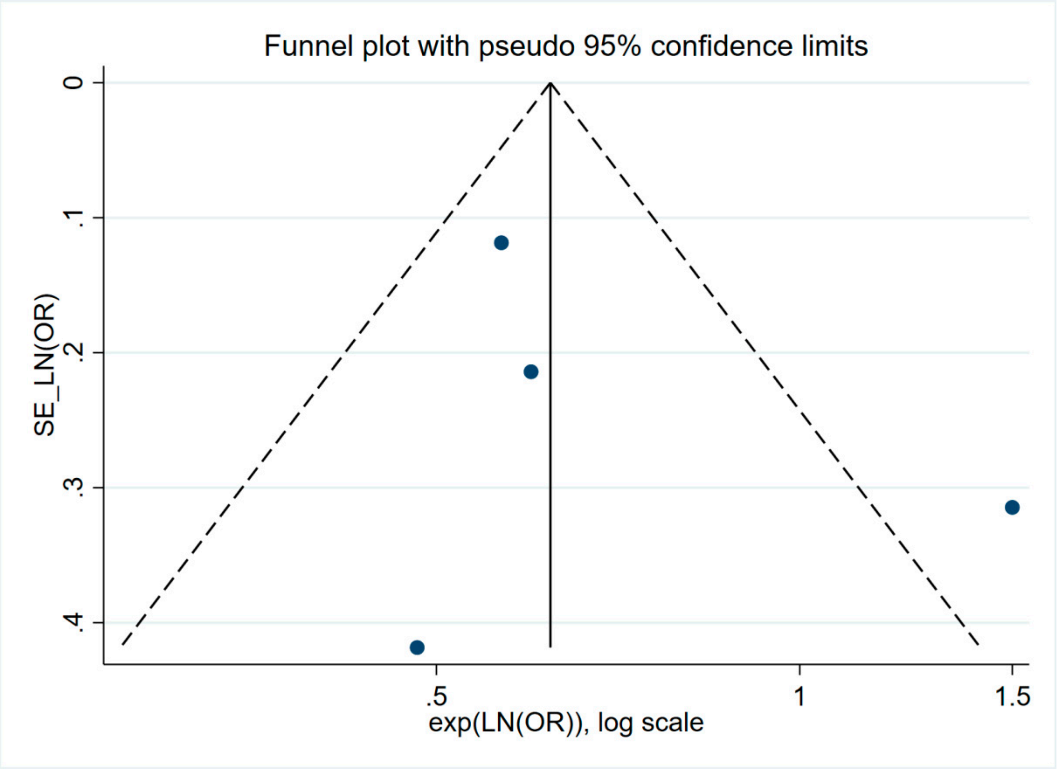

FigureS 28Funnel Plot of Publication Bias in Studies on Male Gender as a Risk Factor for PINV

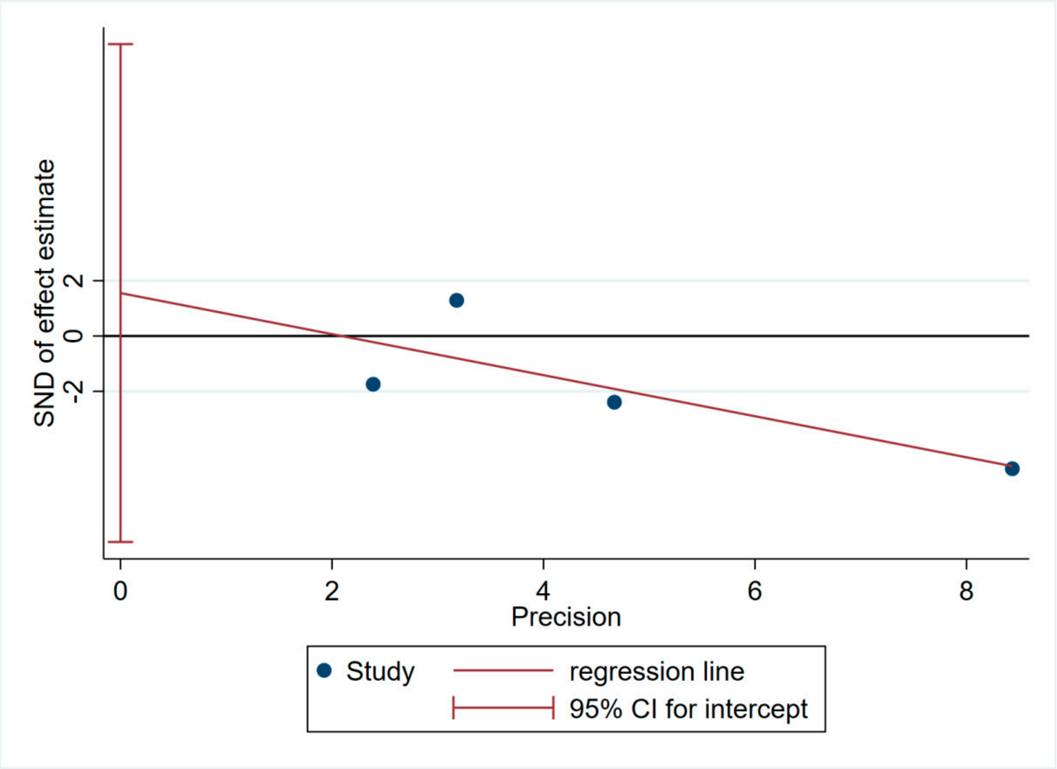

FigureS 29 Egger's Test of Publication Bias for Studies Examining Male Gender as a Risk Factor for PINV

Egger's test for small-study effects:
Regress standard normal deviate of intervention
effect estimate against its standard error

.

Number of studies = 4 Root MSE = 1.864

Std_Eff	Coefficient	Std. err.	t	P>|t|	[95% conf.	interval]
slope	-.7423954	.4012493	-1.85	0.206	-2.468832	.9840412
bias	1.553398	2.092187	0.74	0.535	-7.448558	10.55535

Test of H0: no small-study effects P = 0.535

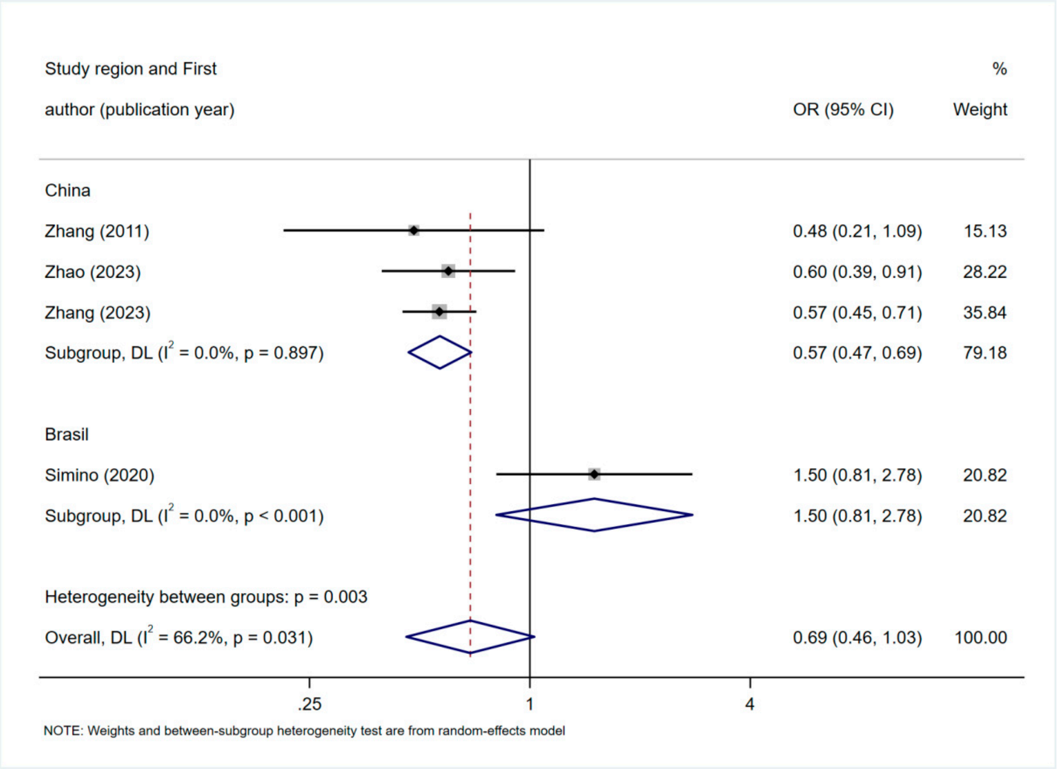

FigureS 30 Forest Plot of Subgroup Analysis by Geographical Region for the Association Between Age and PINV

Geographical Region as a Source of Heterogeneity:

To explore potential sources of heterogeneity, we conducted a subgroup analysis based on geographical regions, as shown in Figure S30. The results demonstrated substantial variation in effect sizes across regions:

China: OR = 0.57 (95% CI: 0.47–0.69), contributing 79.18% of the total weight.

Brazil: OR = 1.50 (95% CI: 0.81–2.78), contributing 20.82% of the total weight.

These findings suggest that geographical region may be an important source of heterogeneity in this meta-analysis (p for subgroup difference = 0.003). In particular, studies conducted in China indicated that younger age may be associated with a lower risk of PINV, whereas the single study from Brazil suggested a potential trend toward increased risk. Variations in population characteristics, chemotherapy regimens, supportive care protocols, and healthcare infrastructure across regions may contribute to these discrepancies.

Female

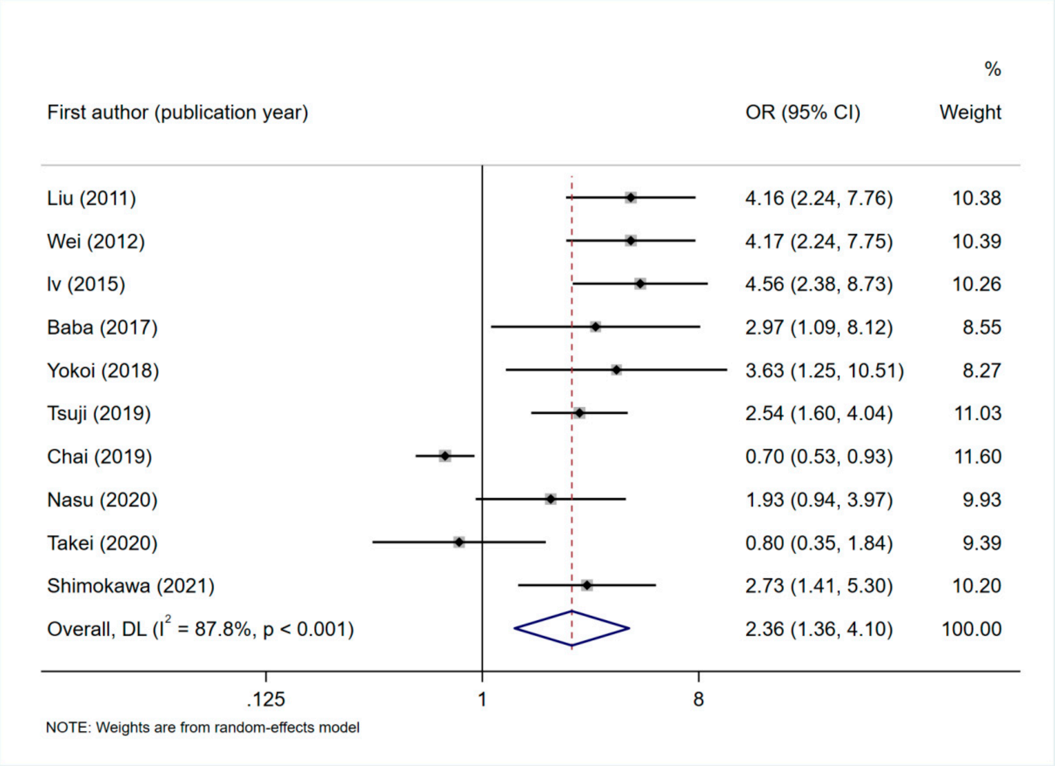

FigureS 31Forest Plot of the Association Between Female Gender and PINV

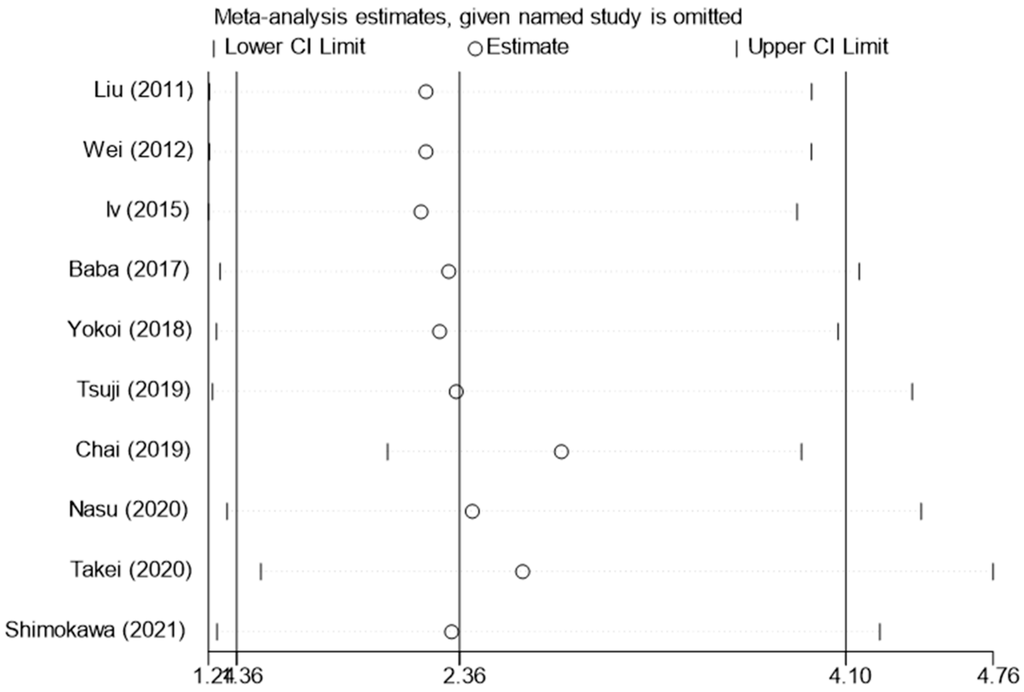

FigureS 32 Sensitivity Analysis of the Association Between Female Gender and PINV

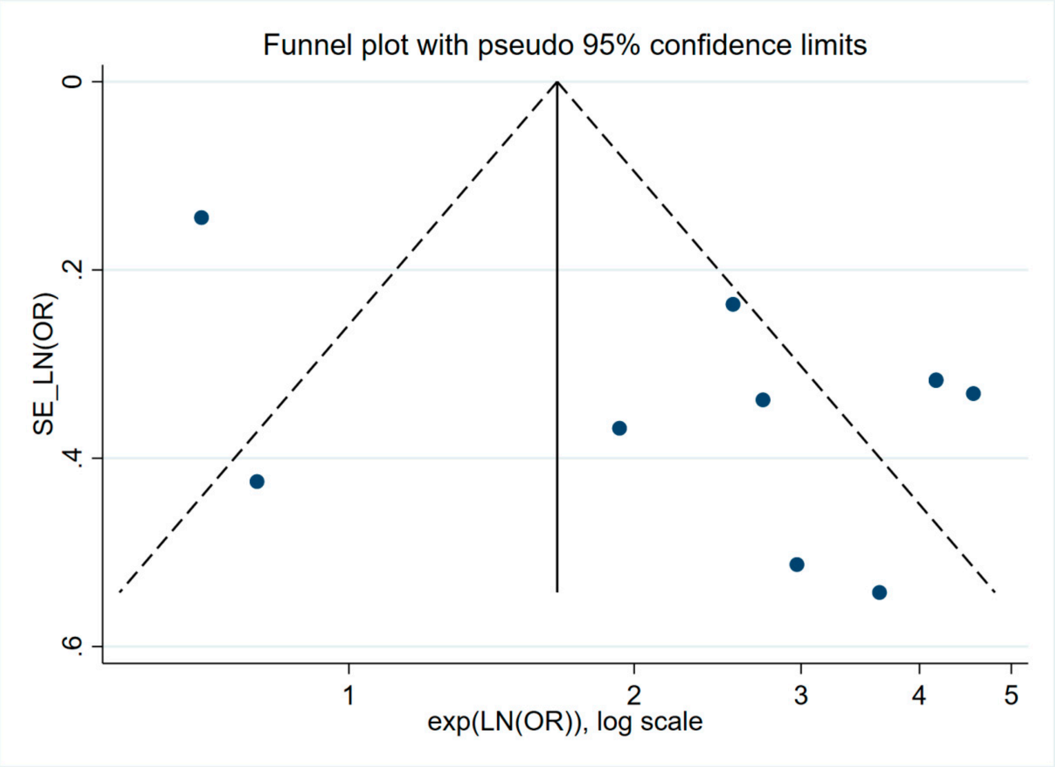

FigureS 33 Funnel Plot of Publication Bias in Studies on Female Gender as a Risk Factor for PINV

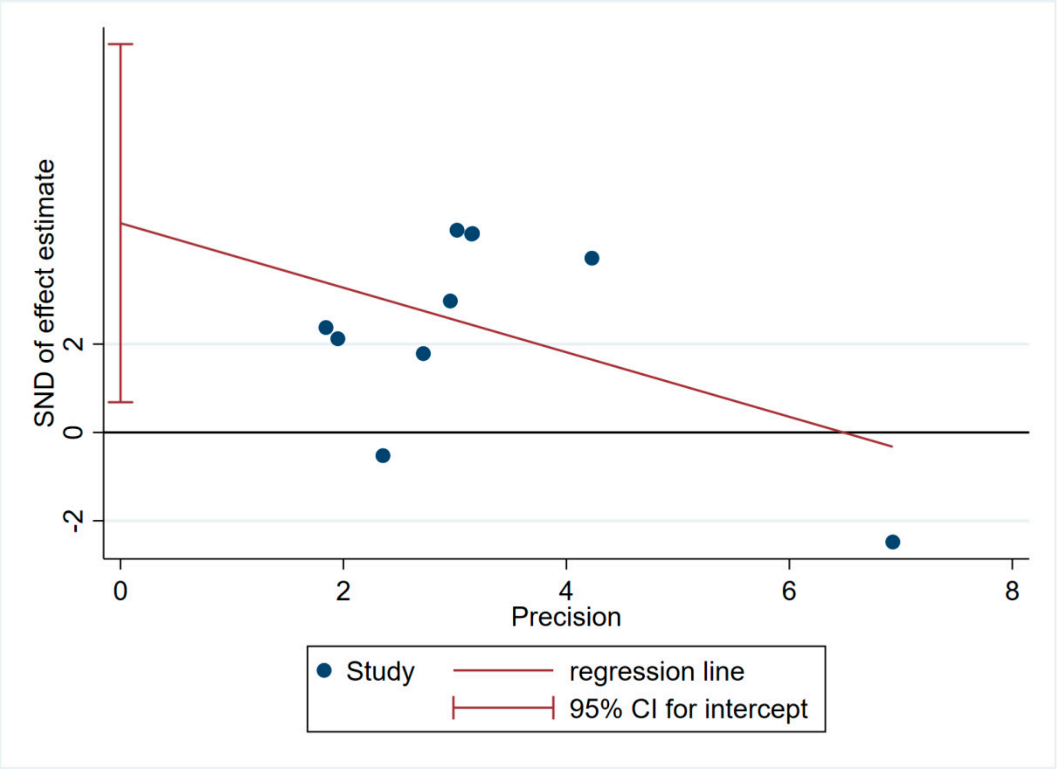

FigureS 34 Egger's Test of Publication Bias for Studies Examining Female Gender as a Risk Factor for PINV

```
gger's test for small-study effects:
egress standard normal deviate of intervention
 effect estimate against its standard error

umber of studies =  10                              Root MSE      =   2.201
```

Std_Eff	Coefficient	Std. err.	t	P>|t|	[95% conf. interval]	
slope	-.7308581	.4998158	-1.46	0.182	-1.883435	.4217193
bias	4.739053	1.758294	2.70	0.027	.684419	8.793687

```
est of H0: no small-study effects          P = 0.027
```

```
Nonparametric trim-and-fill analysis of publication bias
Linear estimator, imputing on the left

Iteration                            Number of studies =     12
  Model: Random-effects                       observed =     10
 Method: REML                                  imputed =      2

Pooling
  Model: Random-effects
 Method: REML
```

Studies	Effect size	[95% conf. interval]	
Observed	0.850	0.404	1.297
Observed + Imputed	0.688	0.268	1.108

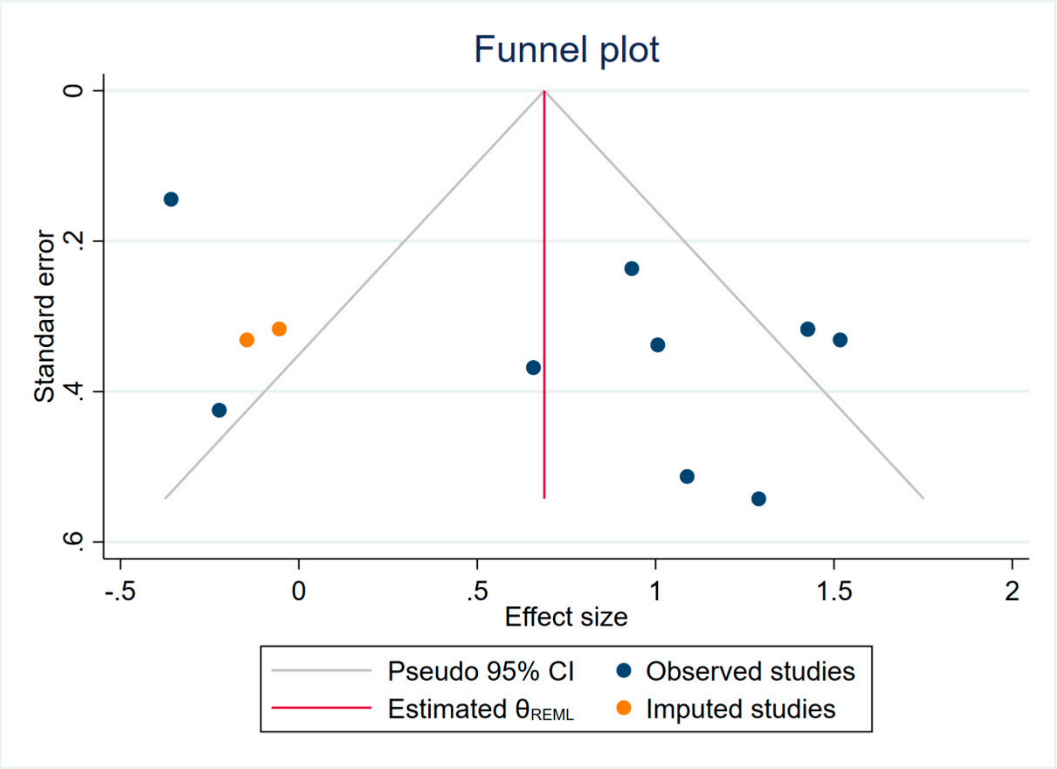

FigureS 35 Funnel Plot with Trim and Fill Correction for the Association Between Female Gender and PINV

```
                                       Number of obs  =       10
 between-study variance                tau2           =    .2835
tion due to heterogeneity              I-squared_res  =   81.87%
tween-study variance explained         Adj R-squared  =   27.92%
ll covariates                          Model F(2,7)   =     2.17
ng modification                        Prob > F       =   0.1851
```

oefficient	Std. err.	t	P>|t|	[95% conf.	interval]
-.121208	.502421	-0.24	0.816	-1.309245	1.066829
.851802	.4738146	1.80	0.115	-.2685915	1.972195
-.1260327	1.070126	-0.12	0.910	-2.656479	2.404414

FigureS 36Regression Analysis of Publication Bias in Studies Examining Female Gender as a Risk Factor for PINV

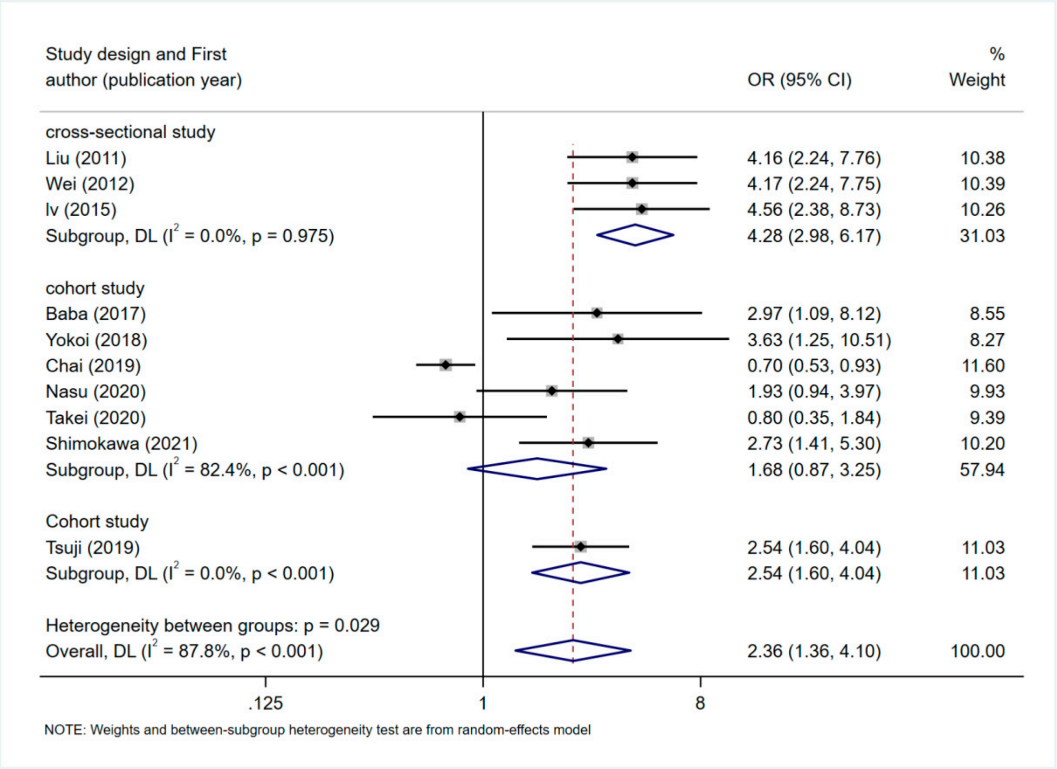

FigureS 37 Forest Plot of Subgroup Analysis by Study Design for the Association Between Female Gender and PINV

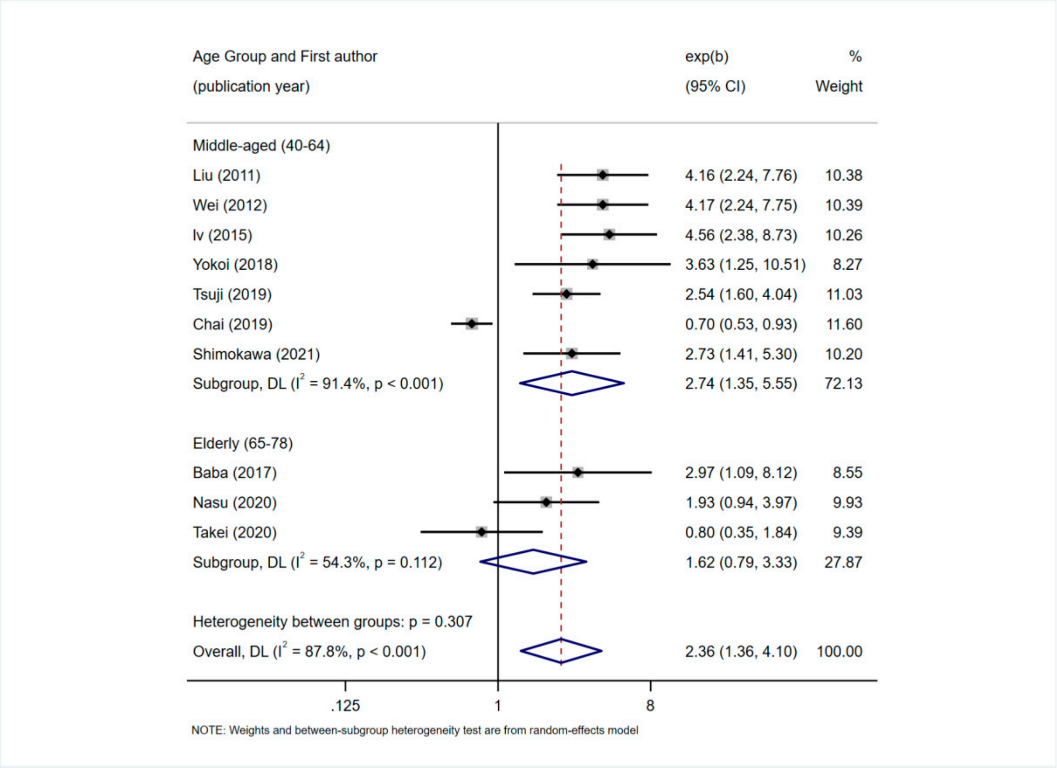

FigureS 38 Forest Plot of Subgroup Analysis by Age Group for the Association Between Female Gender and PINV

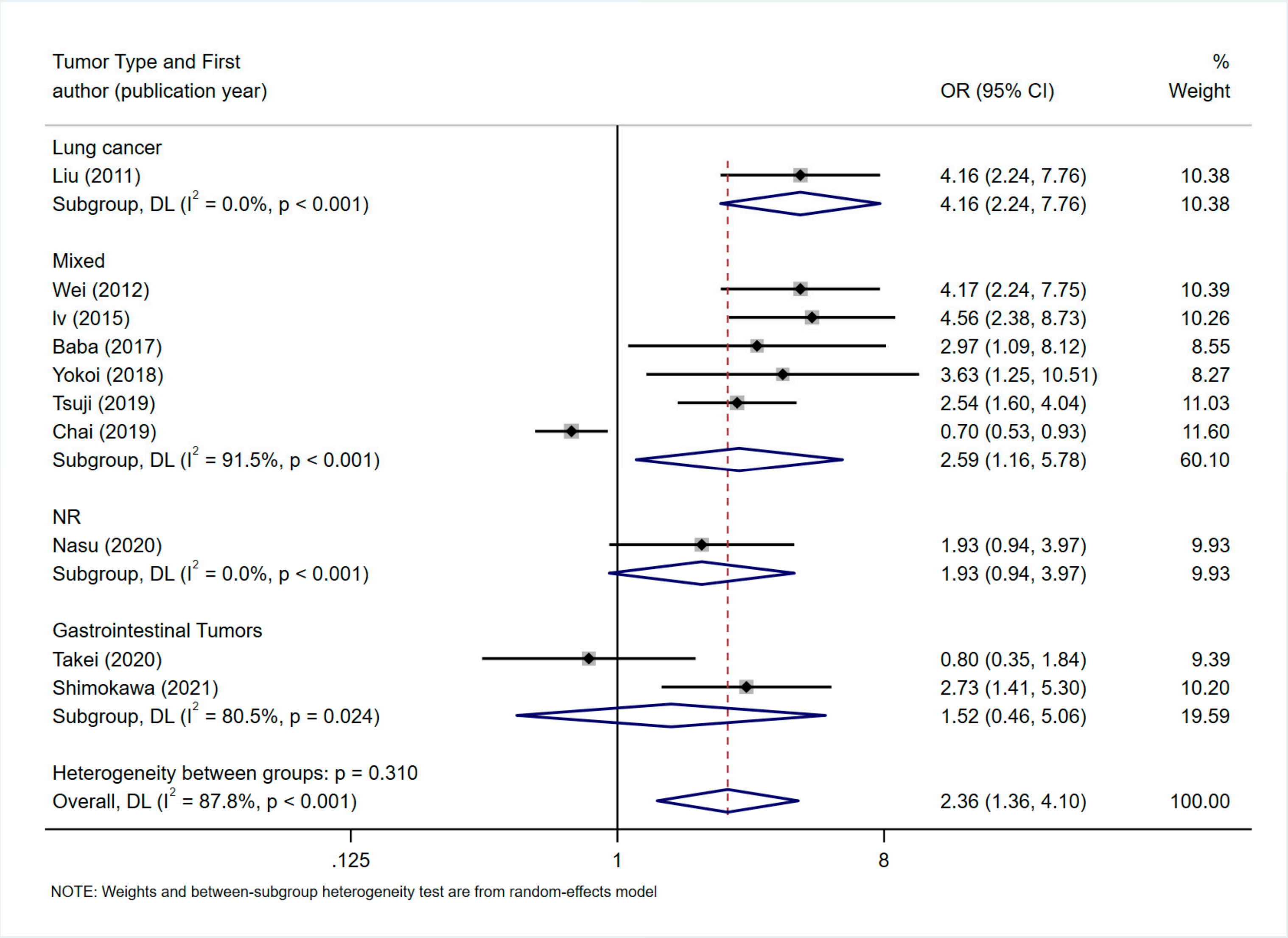
Tumor Type and First
author (publication year)
OR (95% CI)
%
Weight
Lung cancer
Liu (2011)
4.16 (2.24, 7.76)
10.38
Subgroup, DL (I^2 = 0.0%, p < 0.001)
4.16 (2.24, 7.76)
10.38
Mixed
Wei (2012)
4.17 (2.24, 7.75)
10.39
lv (2015)
4.56 (2.38, 8.73)
10.26
Baba (2017)
2.97 (1.09, 8.12)
8.55
Yokoi (2018)
3.63 (1.25, 10.51)
8.27
Tsuji (2019)
2.54 (1.60, 4.04)
11.03
Chai (2019)
0.70 (0.53, 0.93)
11.60
Subgroup, DL (I^2 = 91.5%, p < 0.001)
2.59 (1.16, 5.78)
60.10
NR
Nasu (2020)
1.93 (0.94, 3.97)
9.93
Subgroup, DL (I^2 = 0.0%, p < 0.001)
1.93 (0.94, 3.97)
9.93
Gastrointestinal Tumors
Takei (2020)
0.80 (0.35, 1.84)
9.39
Shimokawa (2021)
2.73 (1.41, 5.30)
10.20
Subgroup, DL (I^2 = 80.5%, p = 0.024)
1.52 (0.46, 5.06)
19.59
Heterogeneity between groups: p = 0.310
Overall, DL (I^2 = 87.8%, p < 0.001)
2.36 (1.36, 4.10)
100.00
.125
1
8
NOTE: Weights and between-subgroup heterogeneity test are from random-effects model

FigureS 39Forest Plot of Subgroup Analysis by Tumor Type for the Association Between Female Gender and PINV

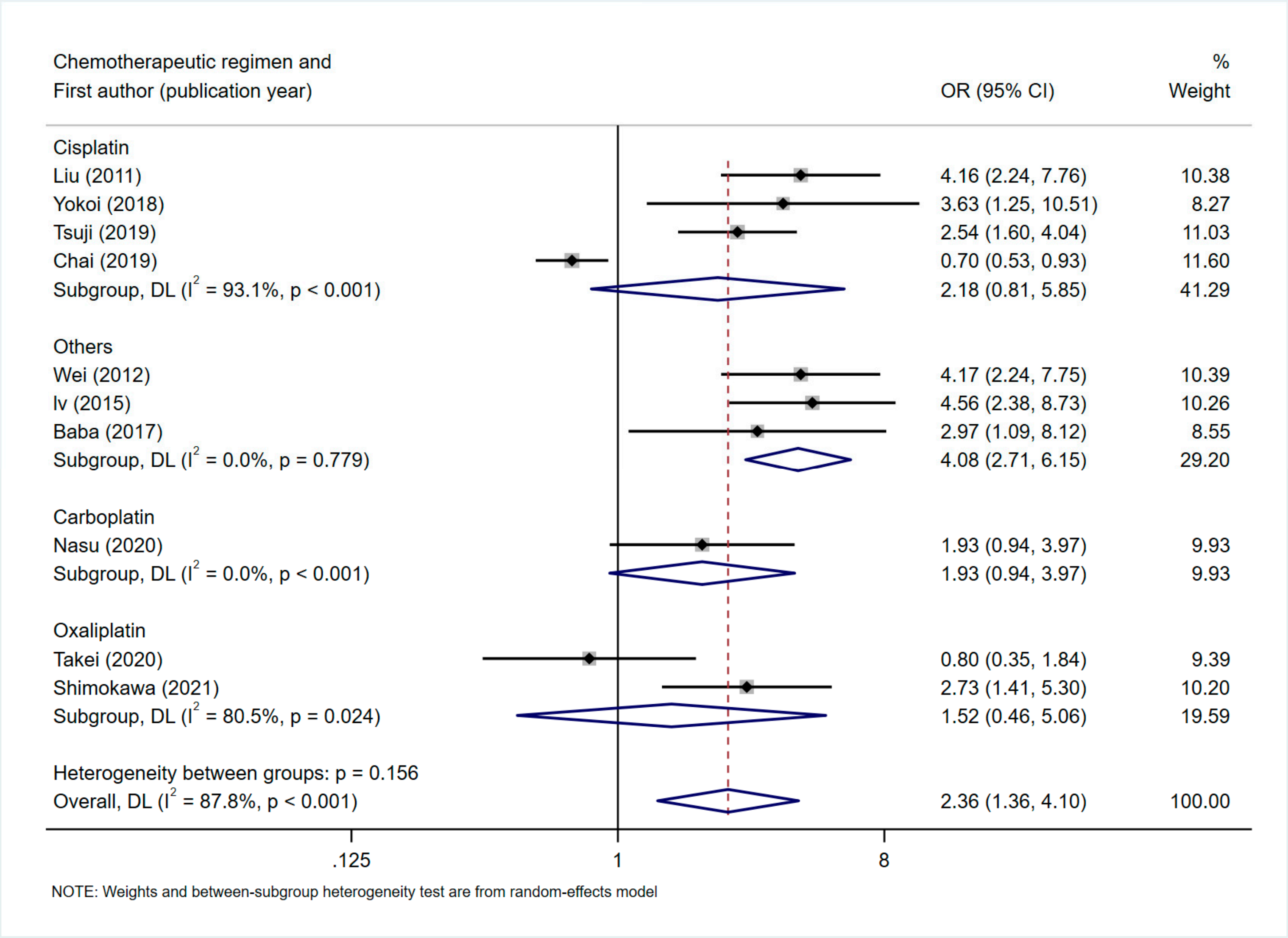
Chemotherapeutic regimen and
First author (publication year)
OR (95% CI)
%
Weight
Cisplatin
Liu (2011)
4.16 (2.24, 7.76)
10.38
Yokoi (2018)
3.63 (1.25, 10.51)
8.27
Tsuji (2019)
2.54 (1.60, 4.04)
11.03
Chai (2019)
0.70 (0.53, 0.93)
11.60
Subgroup, DL (I² = 93.1%, p < 0.001)
2.18 (0.81, 5.85)
41.29
Others
Wei (2012)
4.17 (2.24, 7.75)
10.39
lv (2015)
4.56 (2.38, 8.73)
10.26
Baba (2017)
2.97 (1.09, 8.12)
8.55
Subgroup, DL (I² = 0.0%, p = 0.779)
4.08 (2.71, 6.15)
29.20
Carboplatin
Nasu (2020)
1.93 (0.94, 3.97)
9.93
Subgroup, DL (I² = 0.0%, p < 0.001)
1.93 (0.94, 3.97)
9.93
Oxaliplatin
Takei (2020)
0.80 (0.35, 1.84)
9.39
Shimokawa (2021)
2.73 (1.41, 5.30)
10.20
Subgroup, DL (I² = 80.5%, p = 0.024)
1.52 (0.46, 5.06)
19.59
Heterogeneity between groups: p = 0.156
Overall, DL (I² = 87.8%, p < 0.001)
2.36 (1.36, 4.10)
100.00
.125
1
8
NOTE: Weights and between-subgroup heterogeneity test are from random-effects model

FigureS 40Forest Plot of Subgroup Analysis by Chemotherapeutic regimen for the Association Between Female Gender and PINV

Sources of Heterogeneity：

Despite extensive efforts, including sensitivity analysis, meta-regression, and subgroup analysis, the source of heterogeneity remained unexplained, potentially due to unmeasured confounders or random variation.

Alcohol consumption

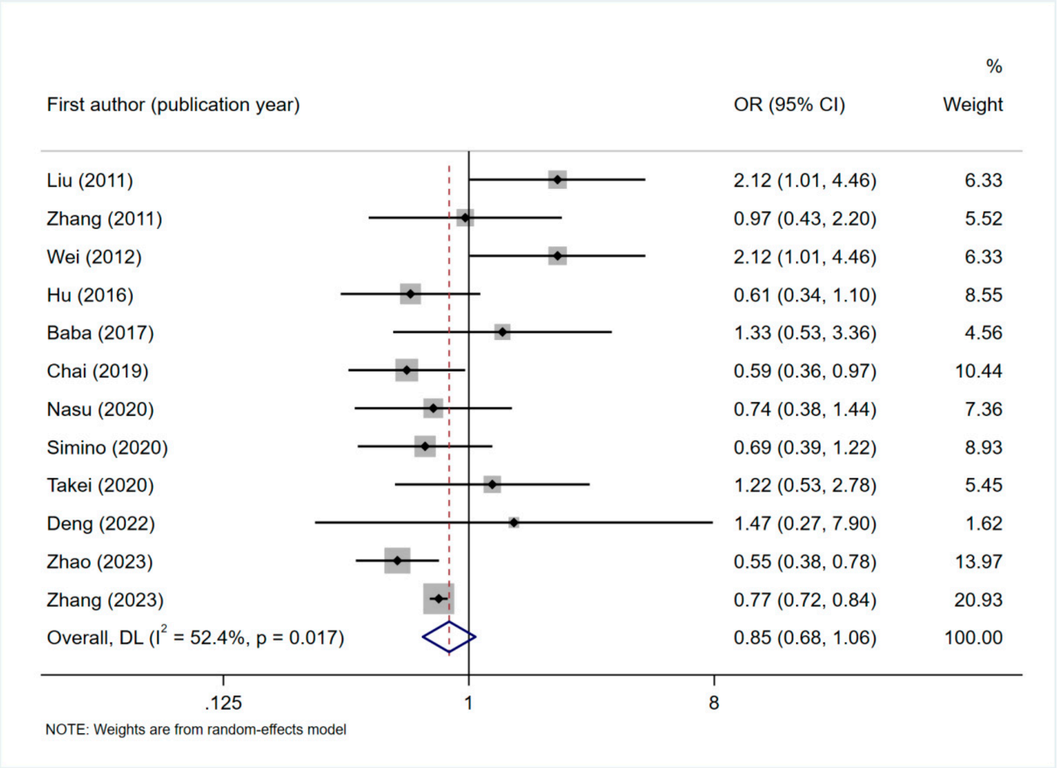

FigureS 41Forest Plot of the Association Between Alcohol Consumption and PINV

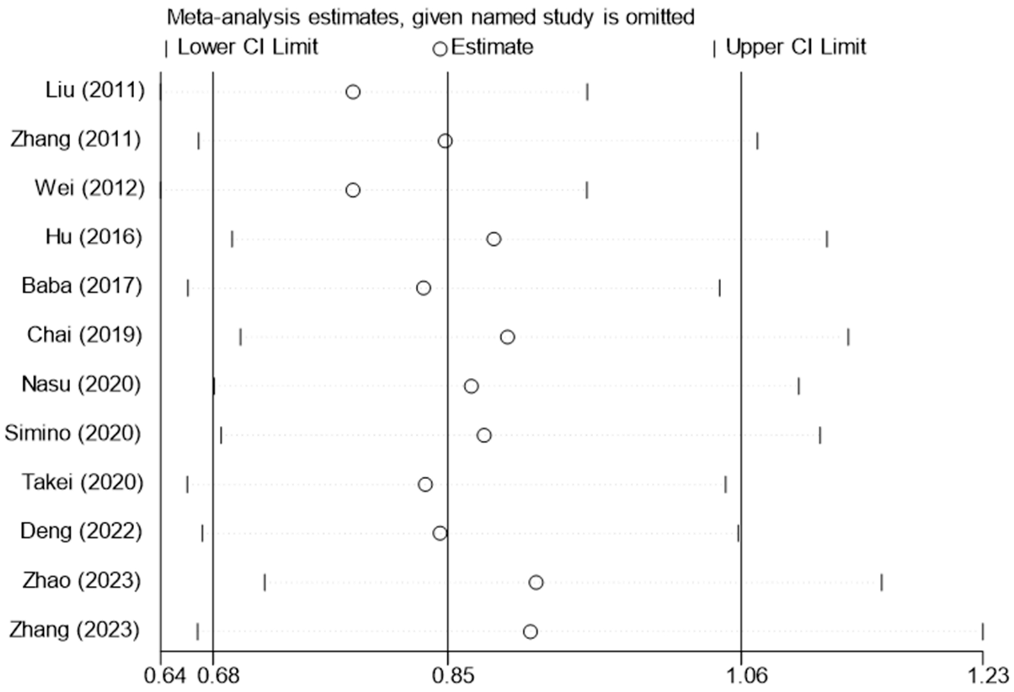

FigureS 42Sensitivity Analysis of the Association Between Alcohol Consumption and PINV

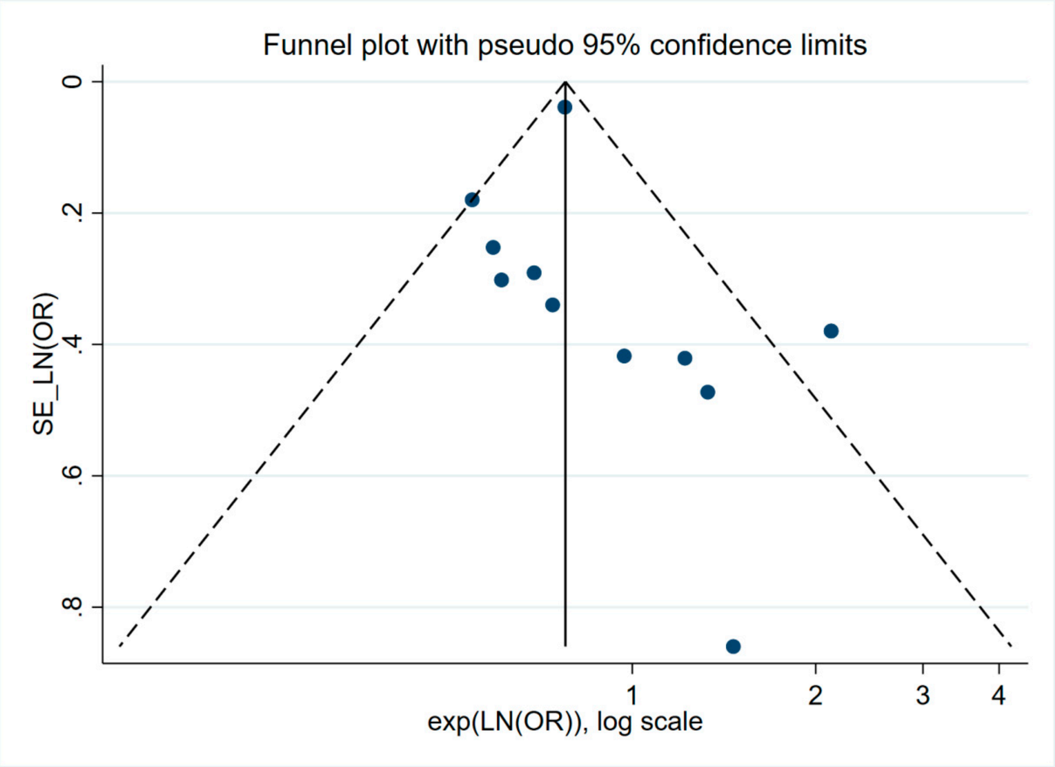

FigureS 43 Funnel Plot of Publication Bias in Studies on Alcohol Consumption as a Risk Factor for PINV

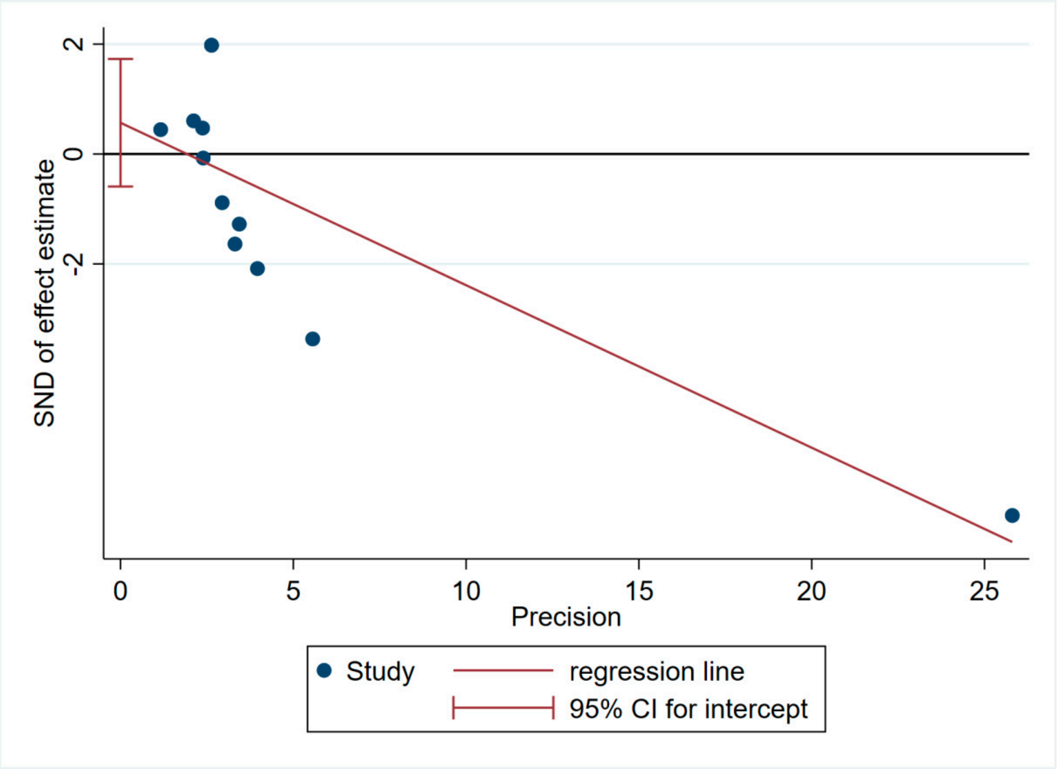

FigureS 44 Egger's Test of Publication Bias for Studies Examining Alcohol Consumption as a Risk Factor for PINV

```
Regress standard normal deviate of intervention
  effect estimate against its standard error

.
Number of studies =  12                              Root MSE      =   1.437
```

Std_Eff	Coefficient	Std. err.	t	P>|t|	[95% conf. interval]	
slope	-.2956591	.0648278	-4.56	0.001	-.4401044	-.1512138
bias	.5691085	.5210113	1.09	0.300	-.591777	1.729994

```
Test of H0: no small-study effects          P = 0.300
```

BMI

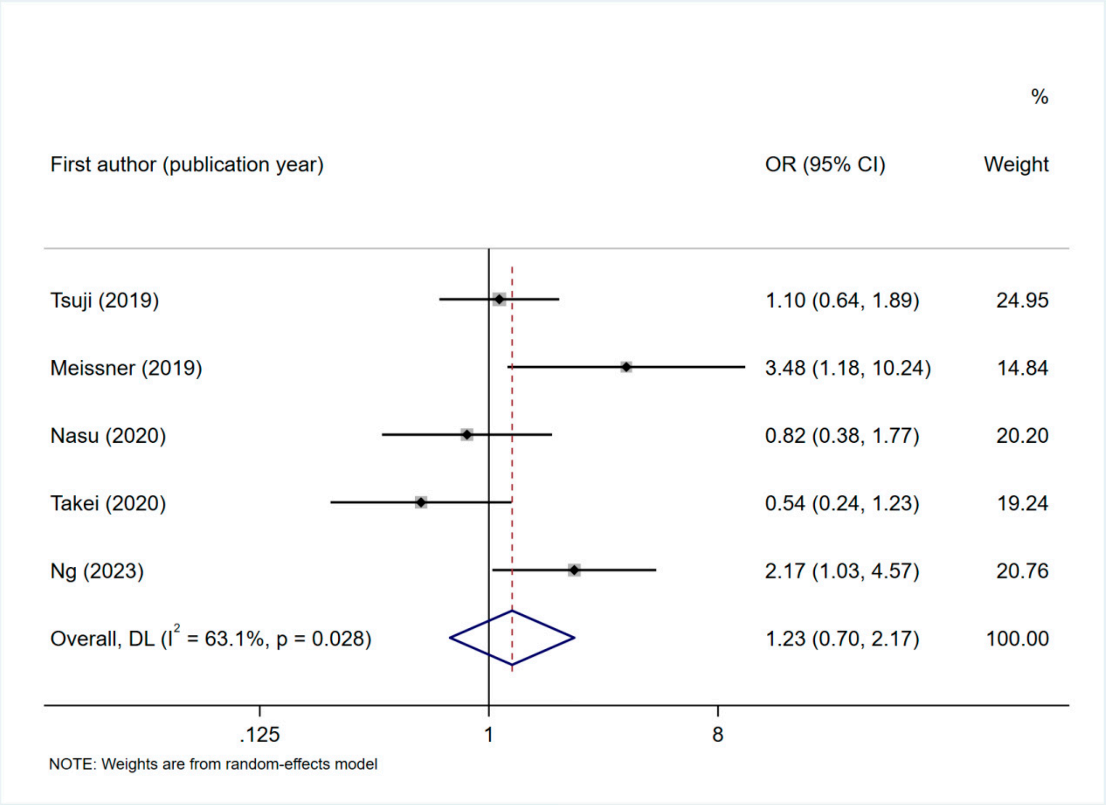

FigureS 45 Forest Plot of the Association Between BMI and PINV

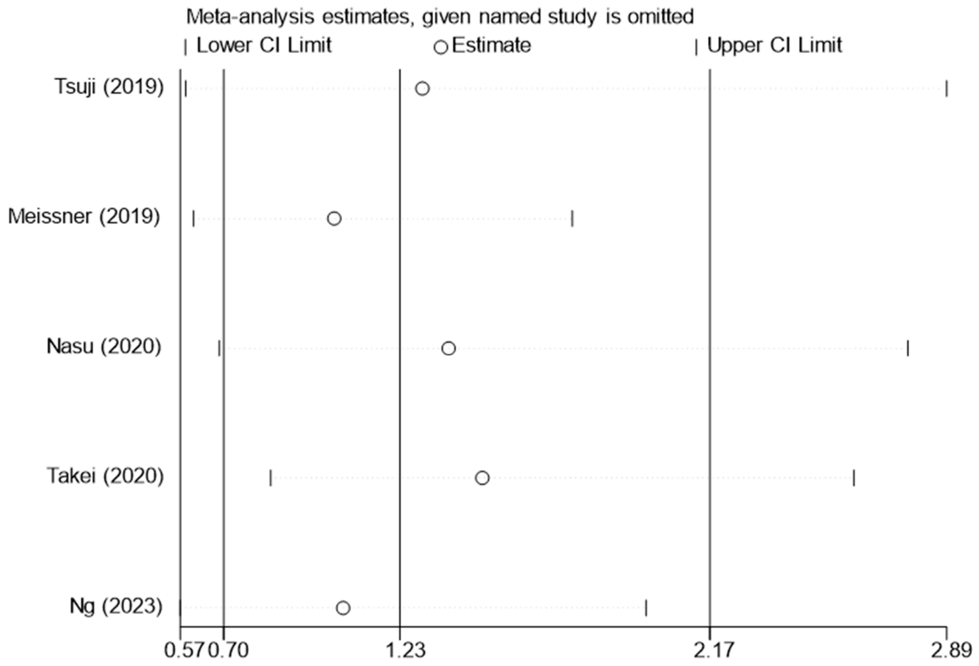

FigureS 46 Sensitivity Analysis of the Association Between BMI and PINV

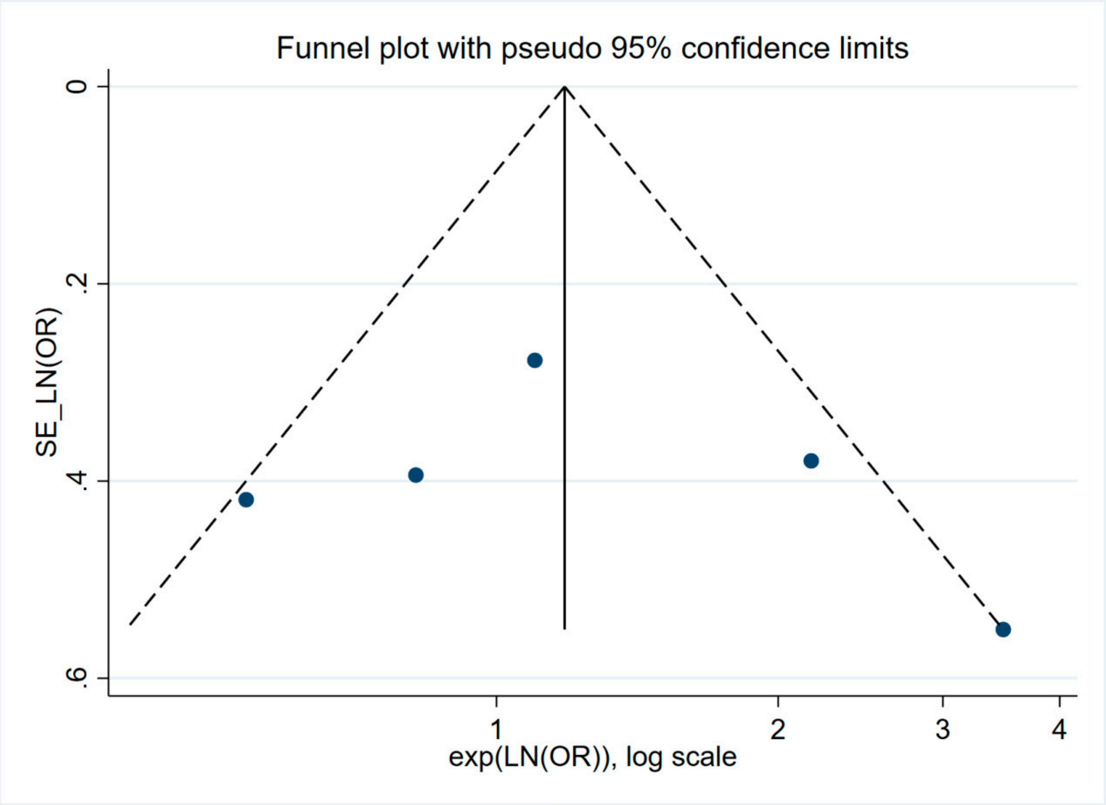

FigureS 47 Funnel Plot of Publication Bias in Studies on BMI as a Risk Factor for PINV

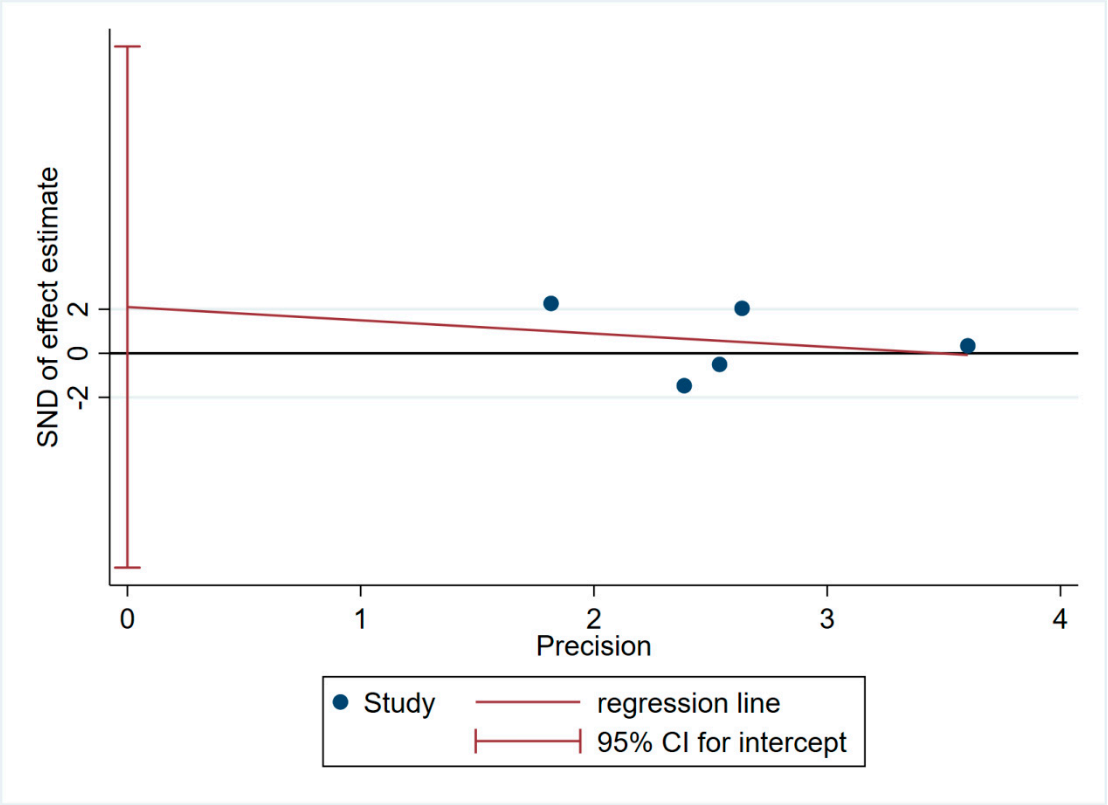

FigureS 48 Egger's Test of Publication Bias for Studies Examining BMI as a Risk Factor for PINV

Egger's test for small-study effects:
Regress standard normal deviate of intervention
effect estimate against its standard error

.
Number of studies = 5 Root MSE = 1.807

Std_Eff	Coefficient	Std. err.	t	P>|t|	[95% conf.	interval]
slope	-.6037634	1.398326	-0.43	0.695	-5.053862	3.846335
bias	2.101695	3.718679	0.57	0.611	-9.732801	13.93619

Test of H0: no small-study effects P = 0.611

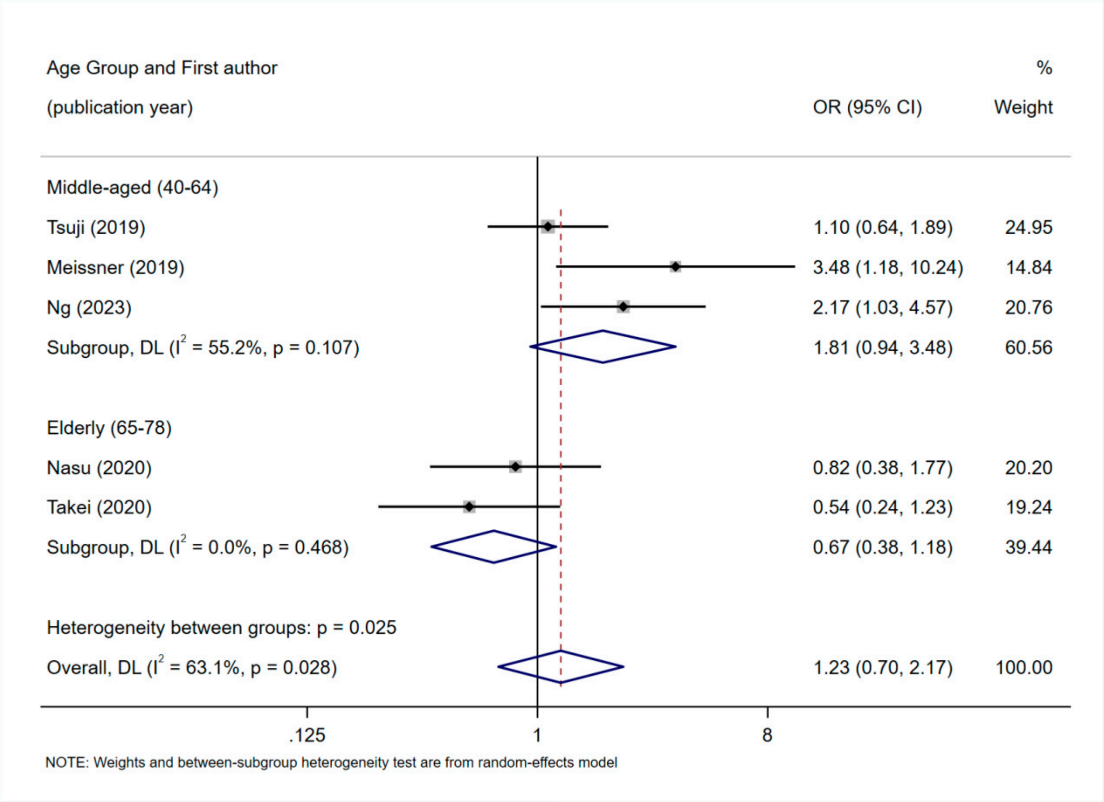

FigureS 49 Forest Plot of Subgroup Analysis by Age Group for the Association Between BMI and PINV

Age Group as a Source of Heterogeneity:

To investigate potential sources of heterogeneity, a subgroup analysis was performed based on age groups, as shown in the forest plot. The results revealed notable differences in effect sizes between middle-aged and elderly patients:

Middle-aged group (40–64 years): OR = 1.81 (95% CI: 0.94–3.48), contributing 60.56% of the total weight.

Elderly group (65–78 years): OR = 0.67 (95% CI: 0.38–1.18), contributing 39.44% of the total weight.

The between-group heterogeneity was statistically significant (p = 0.025), indicating that age group may be a relevant source of heterogeneity in the association between BMI and PINV. Specifically, higher BMI appeared to be associated with a greater risk of PINV among middle-aged patients, whereas this trend was not evident in the elderly group. These differences may reflect age-related variations in body composition, metabolic reserve, chemotherapy metabolism, or symptom perception, which could influence the clinical impact of BMI on nausea and vomiting outcomes.

Smoking

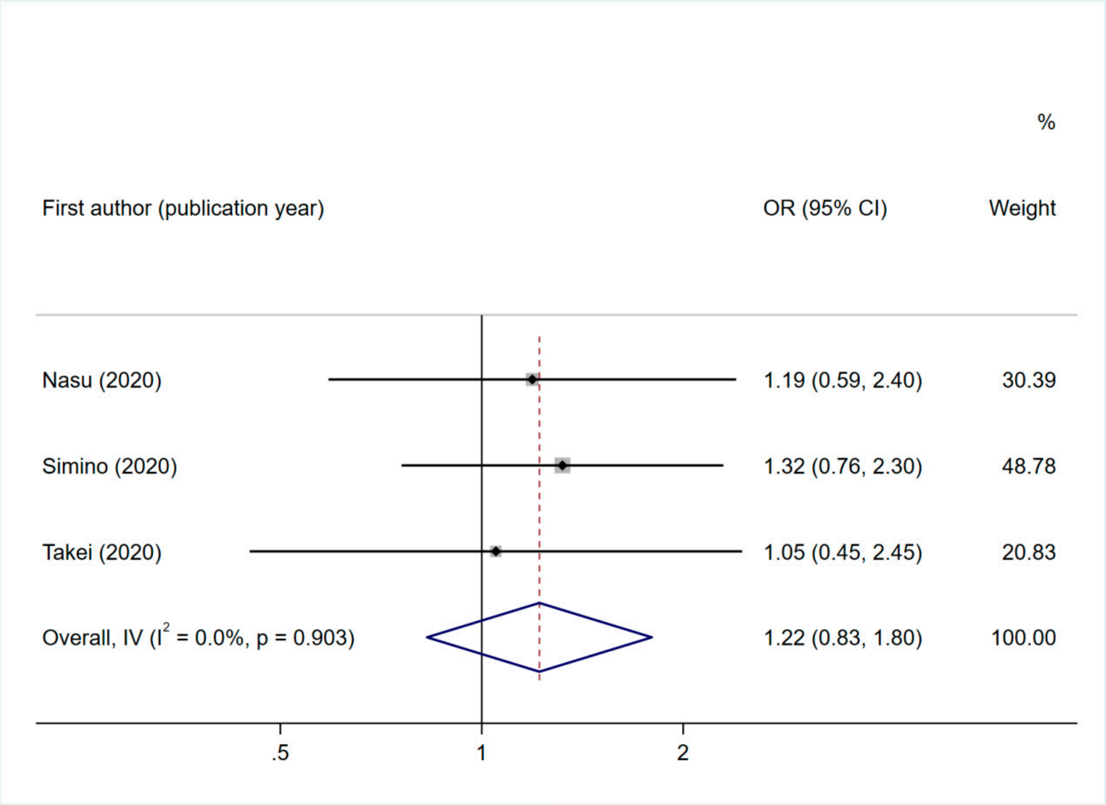

FigureS 50 Forest Plot of the Association Between BMI and PINV

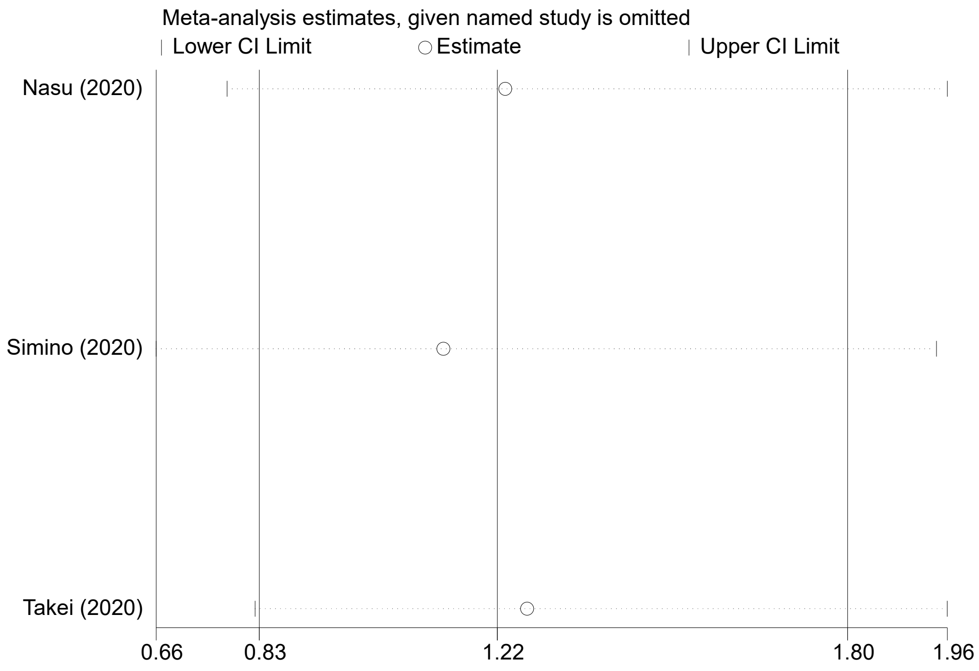

FigureS 51Sensitivity Analysis of the Association Between BMI and PINV

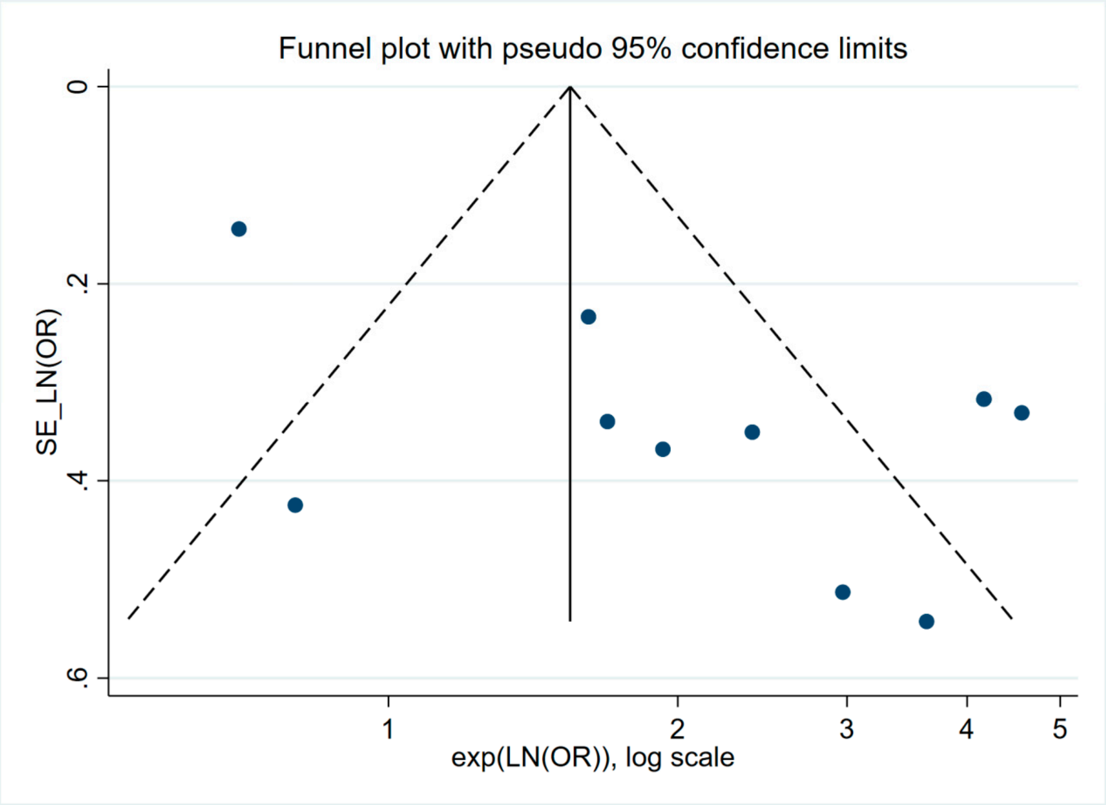

FigureS 52Funnel Plot of Publication Bias in Studies on BMI as a Risk Factor for PINV

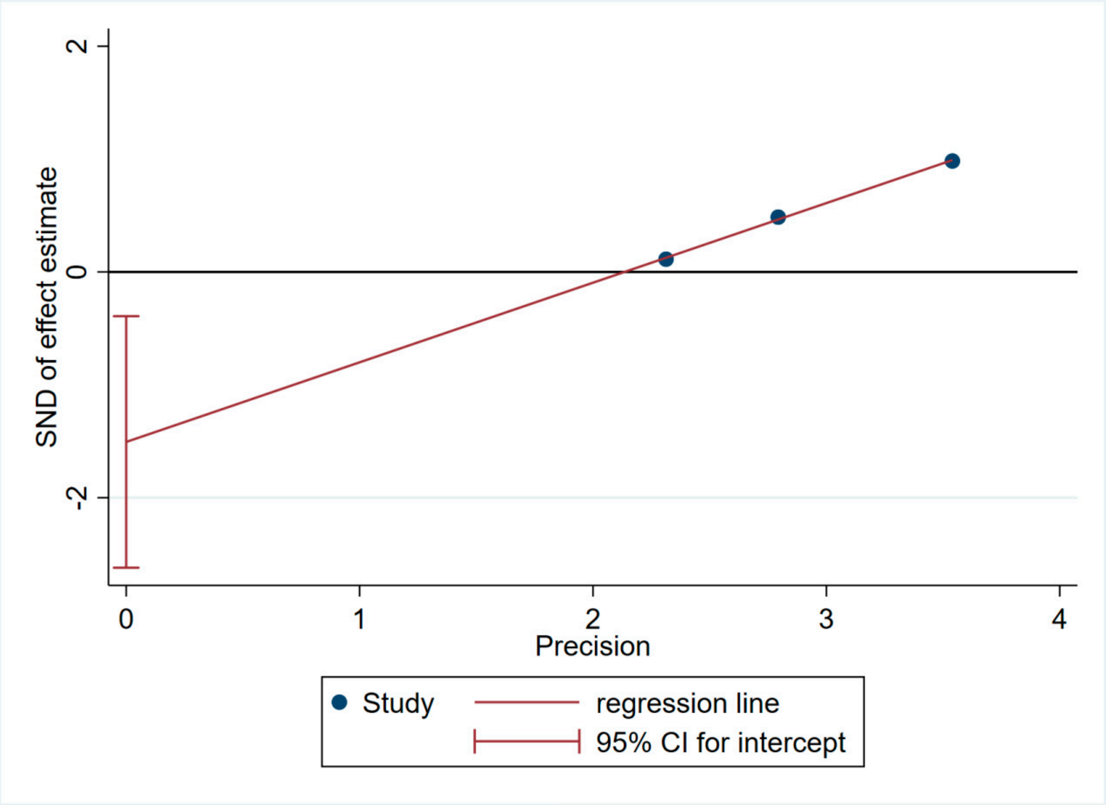

FigureS 53 Egger's Test of Publication Bias for Studies Examining BMI as a Risk Factor for PINV

Egger's test for small-study effects:
Regress standard normal deviate of intervention
effect estimate against its standard error

.
Number of studies = 3 Root MSE = .0262

Std_Eff	Coefficient	Std. err.	t	P>|t|	[95% conf. interval]	
slope	.7054006	.0299645	23.54	0.027	.3246653	1.086136
bias	-1.505986	.0876823	-17.18	0.037	-2.620095	-.3918768

Test of H0: no small-study effects P = 0.037

Nonparametric trim-and-fill analysis of publication bias
Linear estimator, imputing on the right

Iteration Number of studies = 5
Model: Random-effects observed = 3
Method: REML imputed = 2

Pooling
Model: Random-effects
Method: REML

Studies	Effect size	[95% conf. interval]	
Observed	0.198	-0.188	0.585
Observed + Imputed	0.278	-0.037	0.592

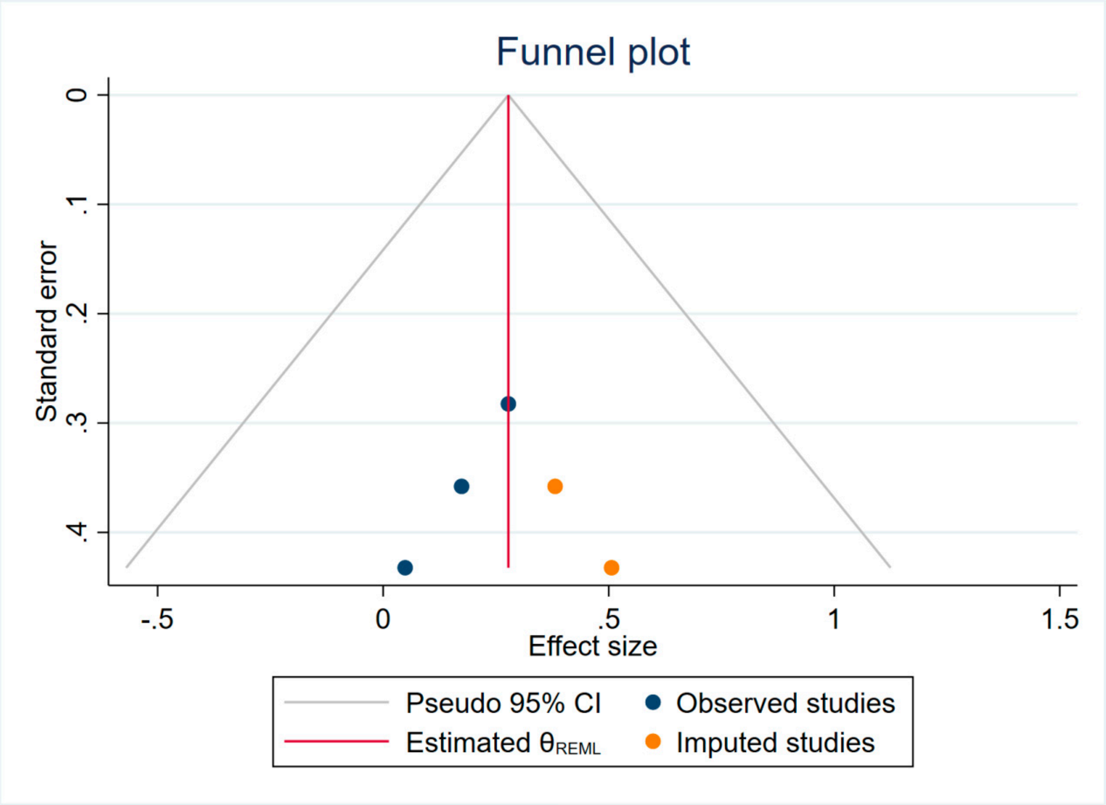

FigureS 54 Funnel Plot with Trim and Fill Correction for the Association Between BMI and PINV

Anxiety

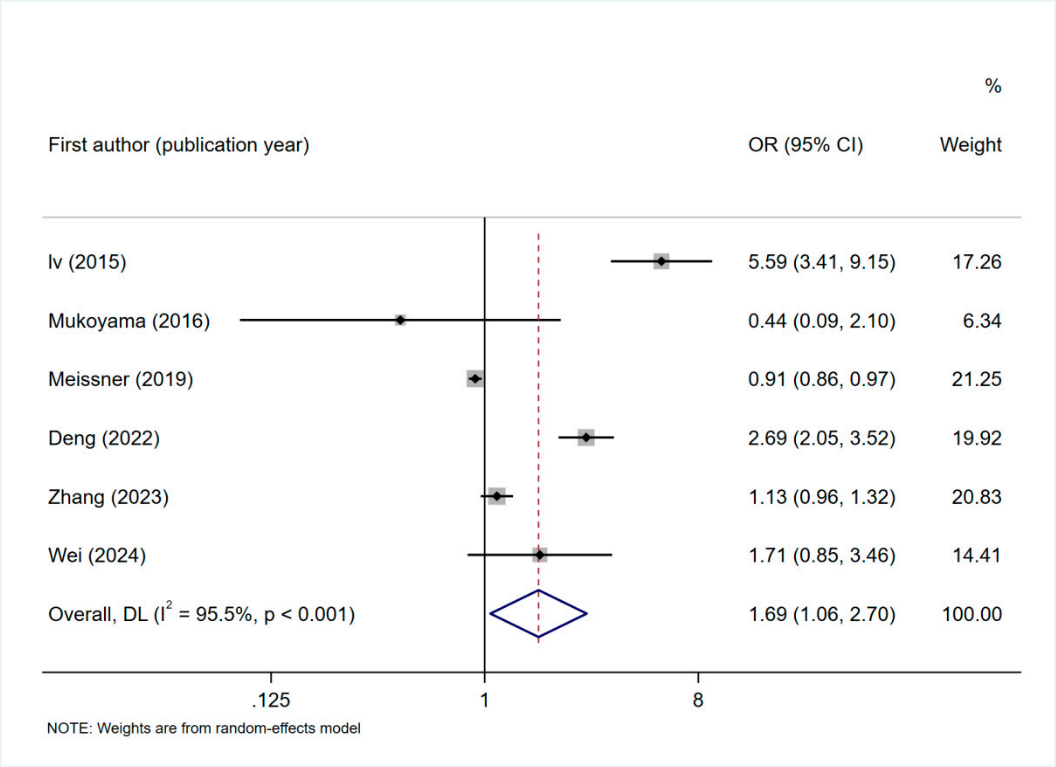

FigureS 55Forest Plot of the Association Between Anxiety and PINV

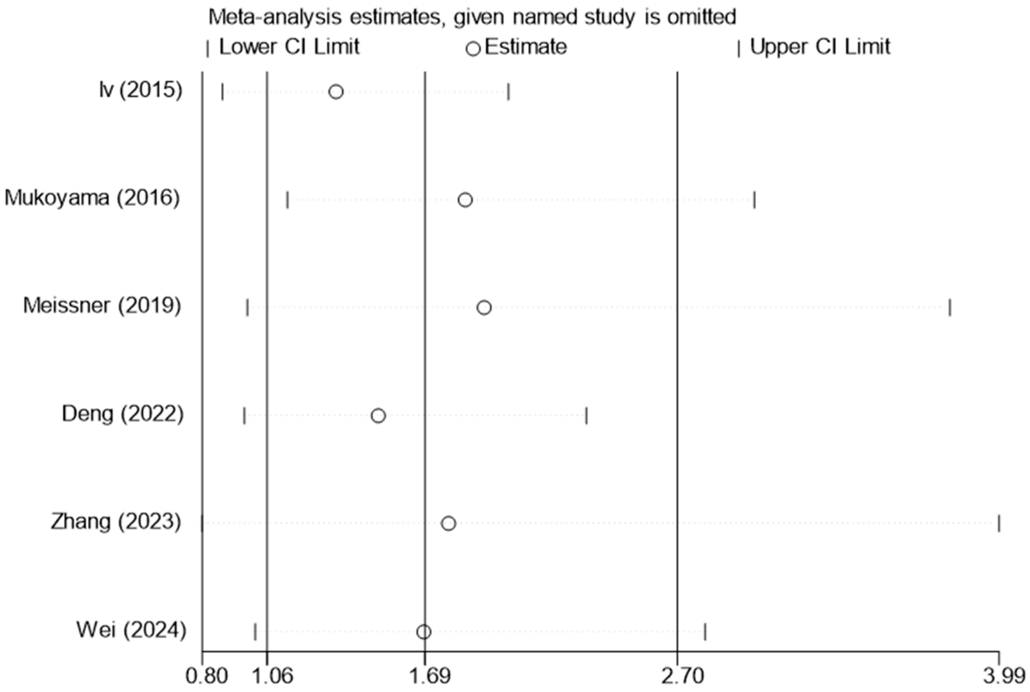

FigureS 56Sensitivity Analysis of the Association Between Anxiety and PINV

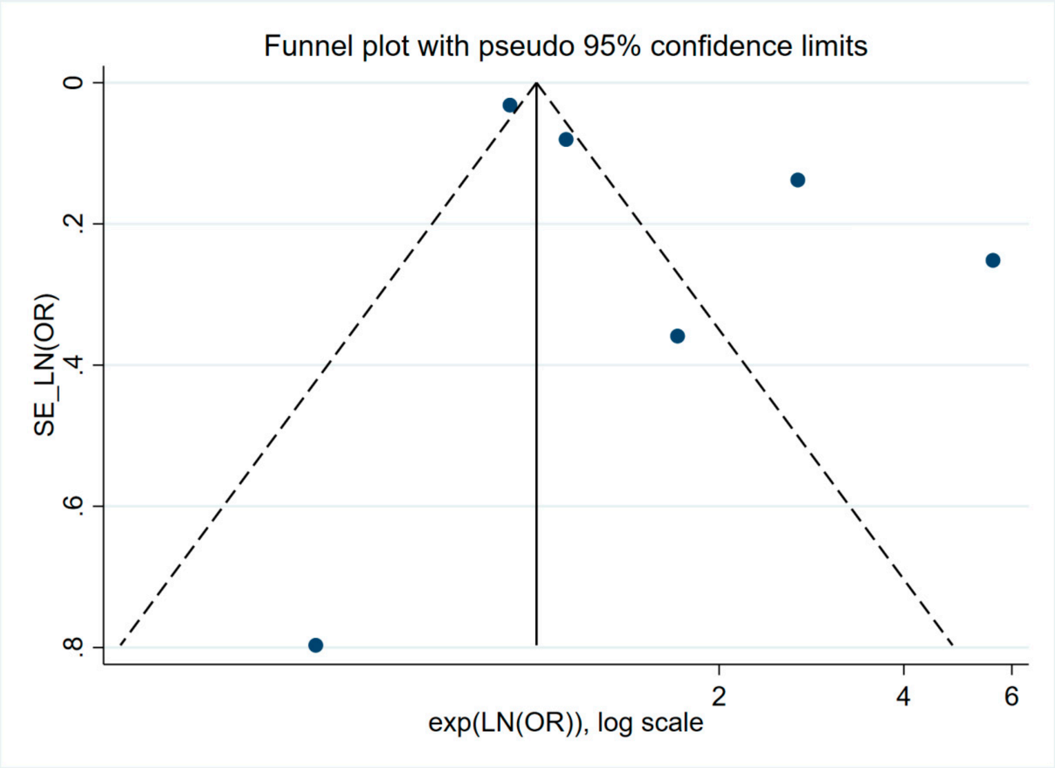

FigureS 57Funnel Plot of Publication Bias in Studies on Anxiety as a Risk Factor for PINV

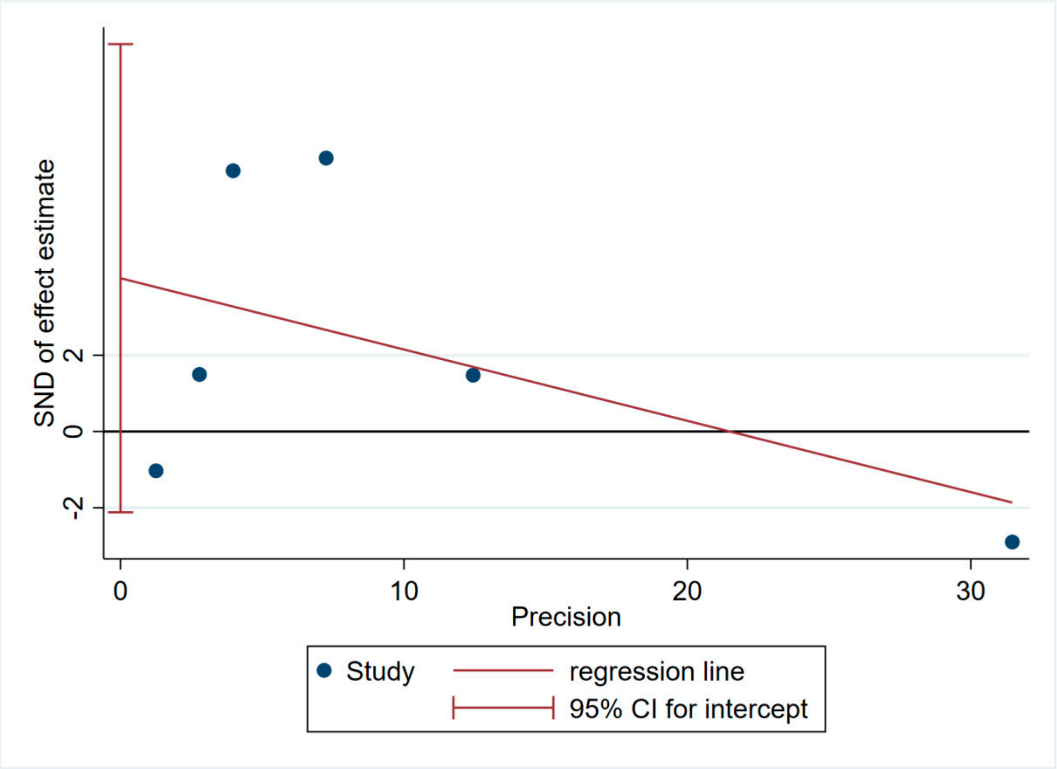

FigureS 58Egger's Test of Publication Bias for Studies Examining Anxiety as a Risk Factor for PINV

```
Egger's test for small-study effects:
Regress standard normal deviate of intervention
  effect estimate against its standard error

.
Number of studies =  6                              Root MSE      =   3.916
------------------------------------------------------------------------------
     Std_Eff | Coefficient  Std. err.      t    P>|t|     [95% conf. interval]
-------------+----------------------------------------------------------------
       slope |  -.1870236   .1549178    -1.21   0.294    -.6171444    .2430972
        bias |   4.020579   2.211442     1.82   0.143    -2.119369    10.16053
------------------------------------------------------------------------------

Test of H0: no small-study effects          P = 0.143


Meta-regression                                     Number of obs   =        6
REML estimate of between-study variance             tau2            =   .03441
% residual variation due to heterogeneity           I-squared_res   =   53.31%
Proportion of between-study variance explained      Adj R-squared   =   93.62%
Joint test for all covariates                       Model F(3,2)    =     5.60
With Knapp-Hartung modification                     Prob > F        =   0.1552
------------------------------------------------------------------------------------
              lnor | Coefficient  Std. err.      t    P>|t|     [95% conf. interval]
-------------------+----------------------------------------------------------------
geographicalregion |  -1.025795   .4063649    -2.52   0.128    -2.774242    .7226521
          agegroup |  -.3823603   .2010198    -1.90   0.198    -1.247279    .4825579
       studydesign |   .7872997    .531347     1.48   0.277    -1.498902    3.073501
             _cons |   1.554535   1.215118     1.28   0.329    -3.673696    6.782766
------------------------------------------------------------------------------------
```

FigureS 59Regression Analysis of Publication Bias in Studies Examining Anxiety as a Risk Factor for PINV

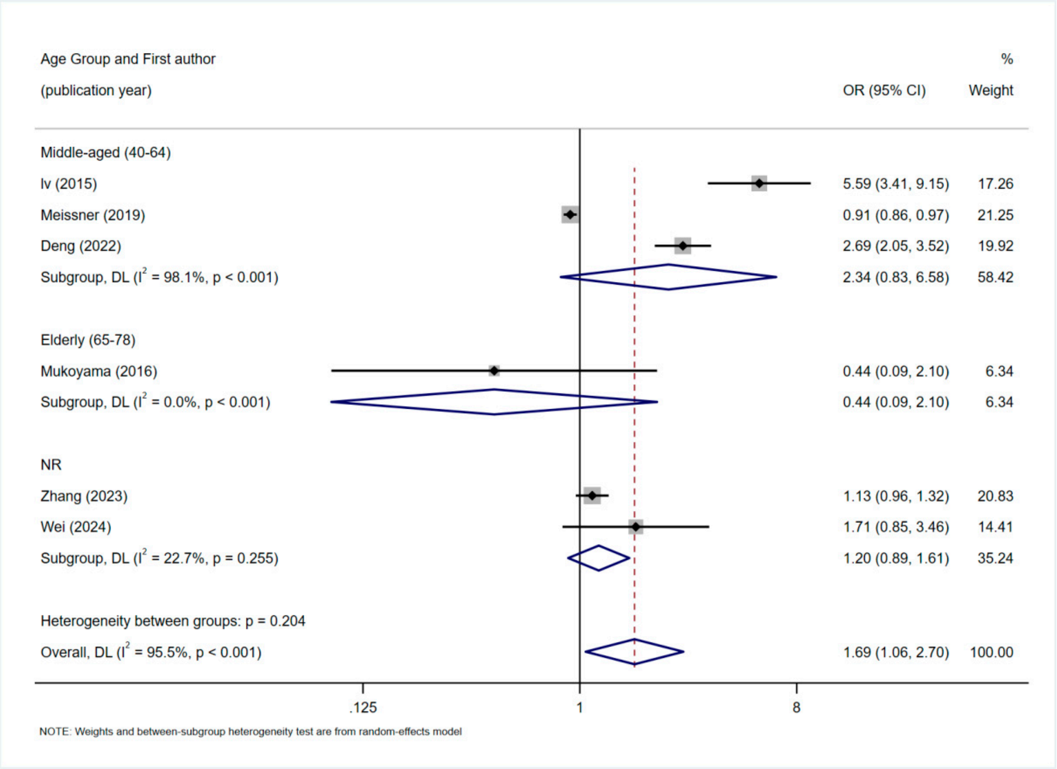

FigureS 60Forest Plot of Subgroup Analysis by Age Group for the Association Between Anxietyand PINV

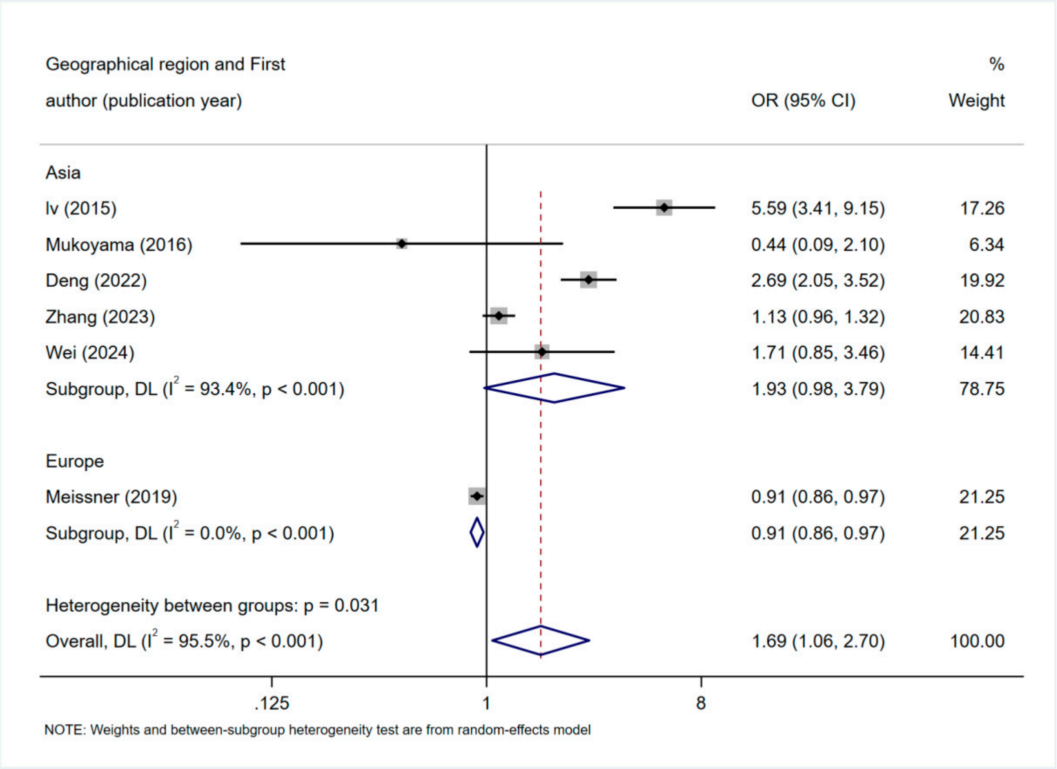

FigureS 61Forest Plot of Subgroup Analysis by Geographical Region for the Association Between Anxiety and PINV

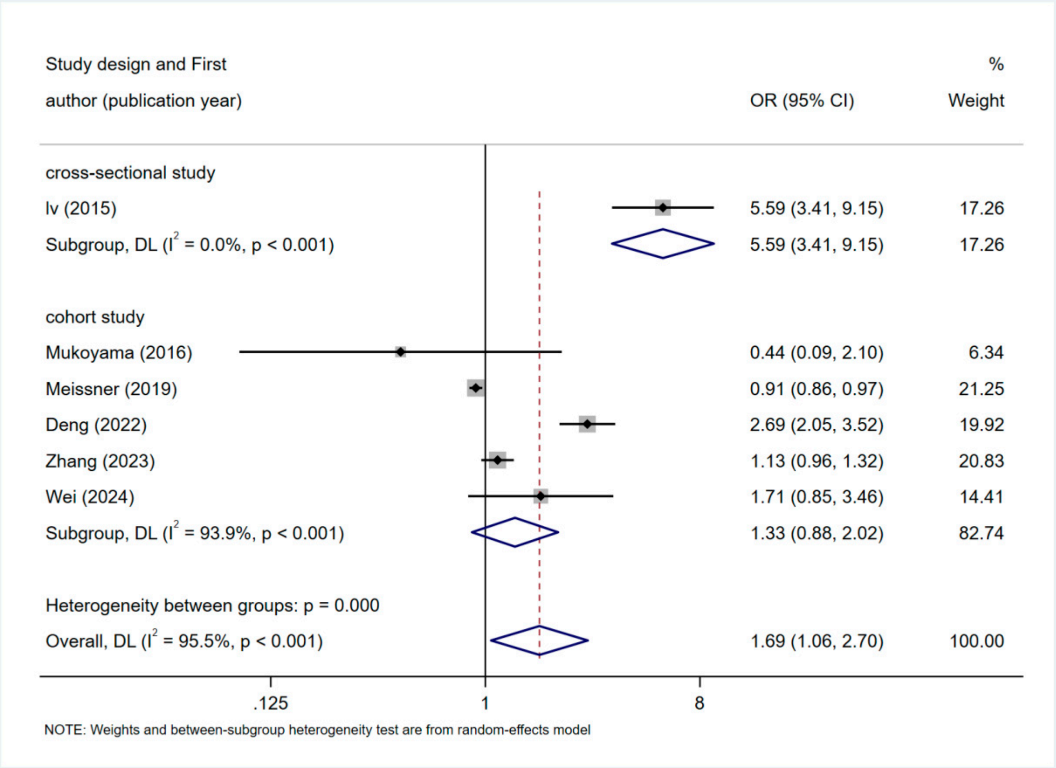

FigureS 62Forest Plot of Subgroup Analysis by Study Design for the Association Between Anxiety and PINV

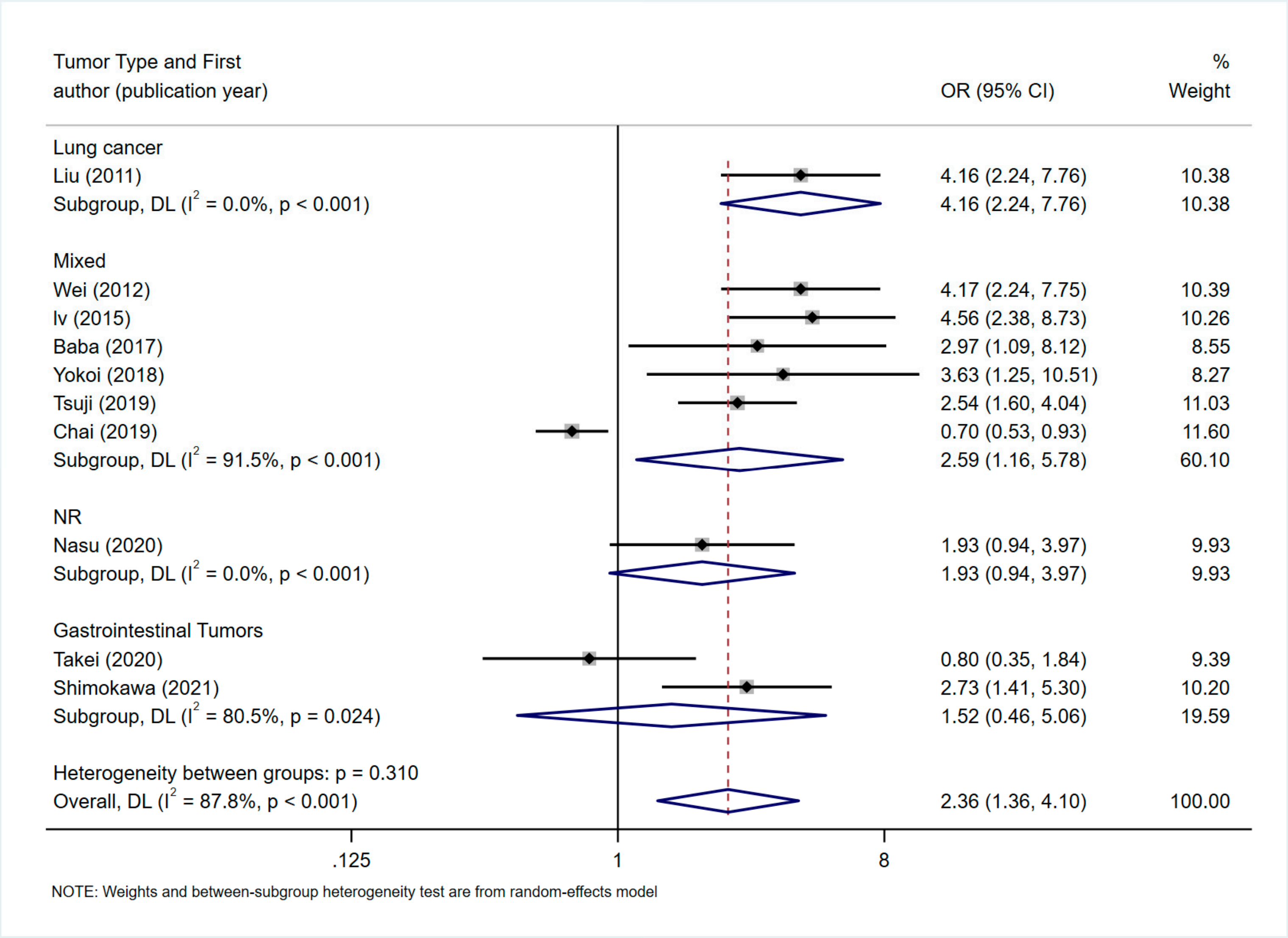
Tumor Type and First
author (publication year)
OR (95% CI)
%
Weight
Lung cancer
Liu (2011) 4.16 (2.24, 7.76) 10.38
Subgroup, DL (I^2 = 0.0%, p < 0.001) 4.16 (2.24, 7.76) 10.38
Mixed
Wei (2012) 4.17 (2.24, 7.75) 10.39
lv (2015) 4.56 (2.38, 8.73) 10.26
Baba (2017) 2.97 (1.09, 8.12) 8.55
Yokoi (2018) 3.63 (1.25, 10.51) 8.27
Tsuji (2019) 2.54 (1.60, 4.04) 11.03
Chai (2019) 0.70 (0.53, 0.93) 11.60
Subgroup, DL (I^2 = 91.5%, p < 0.001) 2.59 (1.16, 5.78) 60.10
NR
Nasu (2020) 1.93 (0.94, 3.97) 9.93
Subgroup, DL (I^2 = 0.0%, p < 0.001) 1.93 (0.94, 3.97) 9.93
Gastrointestinal Tumors
Takei (2020) 0.80 (0.35, 1.84) 9.39
Shimokawa (2021) 2.73 (1.41, 5.30) 10.20
Subgroup, DL (I^2 = 80.5%, p = 0.024) 1.52 (0.46, 5.06) 19.59
Heterogeneity between groups: p = 0.310
Overall, DL (I^2 = 87.8%, p < 0.001) 2.36 (1.36, 4.10) 100.00
.125
1
8
NOTE: Weights and between-subgroup heterogeneity test are from random-effects model

FigureS 63Forest Plot of Subgroup Analysis by Tumor Type for the Association Between Anxiety and PINV

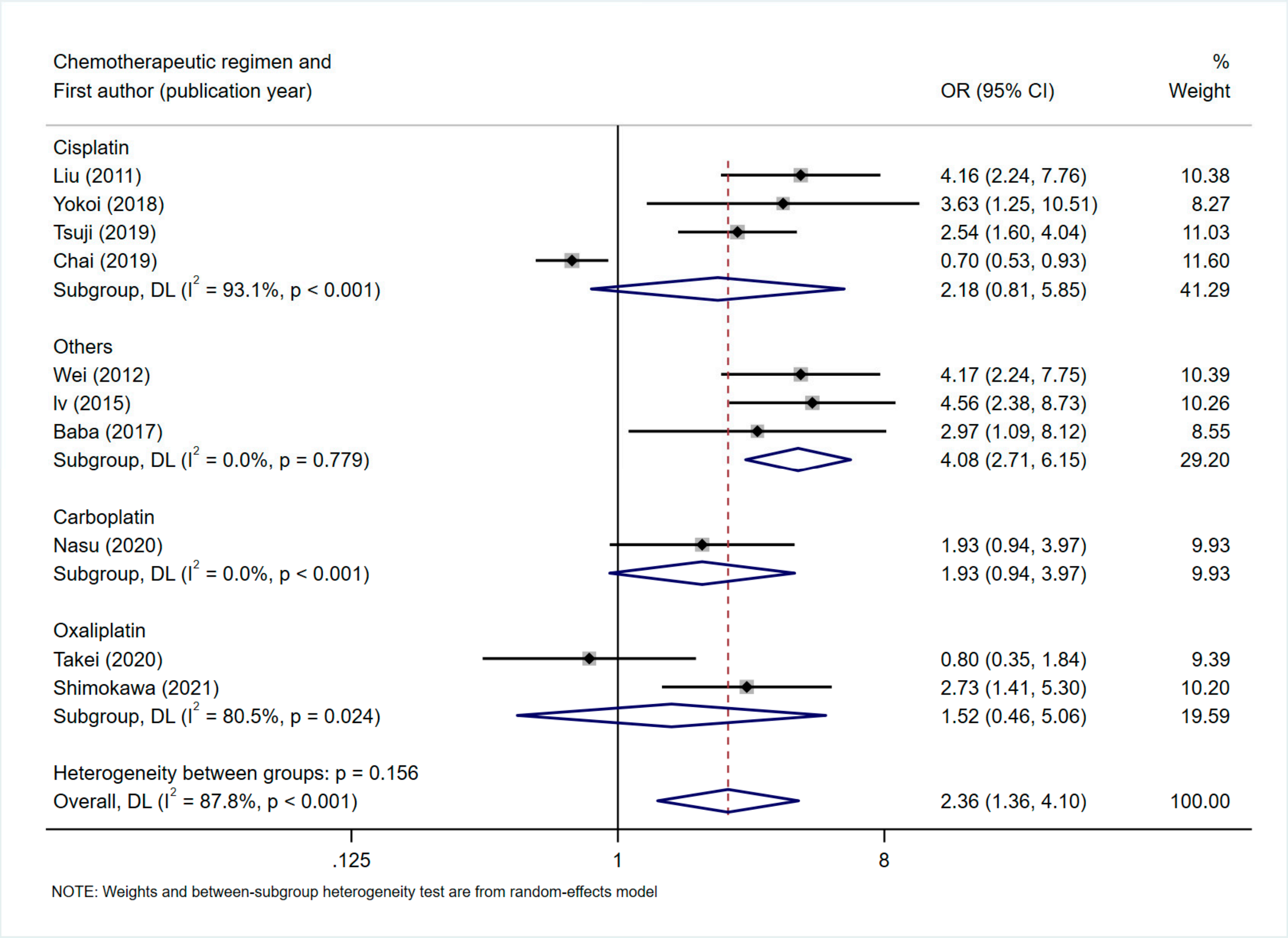
Chemotherapeutic regimen and
First author (publication year)
OR (95% CI)
%
Weight
Cisplatin
Liu (2011) 4.16 (2.24, 7.76) 10.38
Yokoi (2018) 3.63 (1.25, 10.51) 8.27
Tsuji (2019) 2.54 (1.60, 4.04) 11.03
Chai (2019) 0.70 (0.53, 0.93) 11.60
Subgroup, DL (I² = 93.1%, p < 0.001) 2.18 (0.81, 5.85) 41.29
Others
Wei (2012) 4.17 (2.24, 7.75) 10.39
lv (2015) 4.56 (2.38, 8.73) 10.26
Baba (2017) 2.97 (1.09, 8.12) 8.55
Subgroup, DL (I² = 0.0%, p = 0.779) 4.08 (2.71, 6.15) 29.20
Carboplatin
Nasu (2020) 1.93 (0.94, 3.97) 9.93
Subgroup, DL (I² = 0.0%, p < 0.001) 1.93 (0.94, 3.97) 9.93
Oxaliplatin
Takei (2020) 0.80 (0.35, 1.84) 9.39
Shimokawa (2021) 2.73 (1.41, 5.30) 10.20
Subgroup, DL (I² = 80.5%, p = 0.024) 1.52 (0.46, 5.06) 19.59
Heterogeneity between groups: p = 0.156
Overall, DL (I² = 87.8%, p < 0.001) 2.36 (1.36, 4.10) 100.00
.125
1
8
NOTE: Weights and between-subgroup heterogeneity test are from random-effects model

FigureS 64Forest Plot of Subgroup Analysis by Chemotherapeutic regimen for the Association Between Anxiety and PINV

Sources of Heterogeneity：

Despite extensive efforts, including sensitivity analysis, meta-regression, and subgroup analysis, the source of heterogeneity remained unexplained, potentially due to unmeasured confounders or random variation.

Fatigue

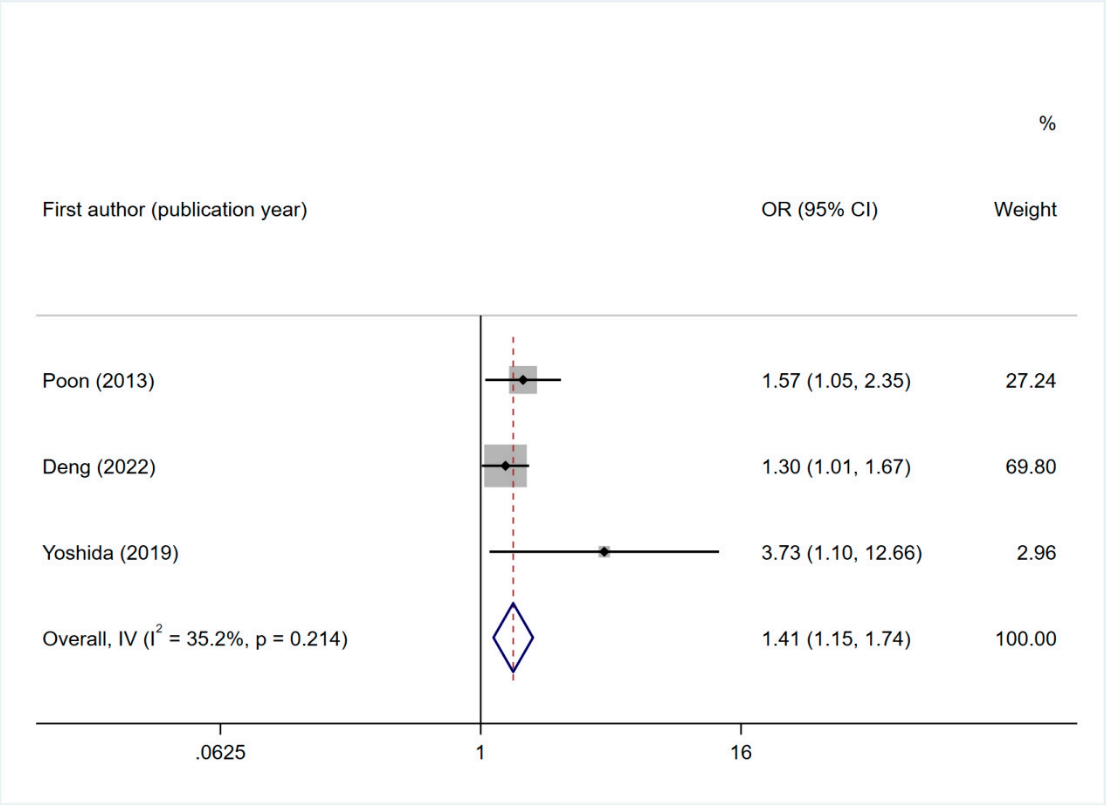

FigureS 65 Forest Plot of the Association Between Fatigue and PINV

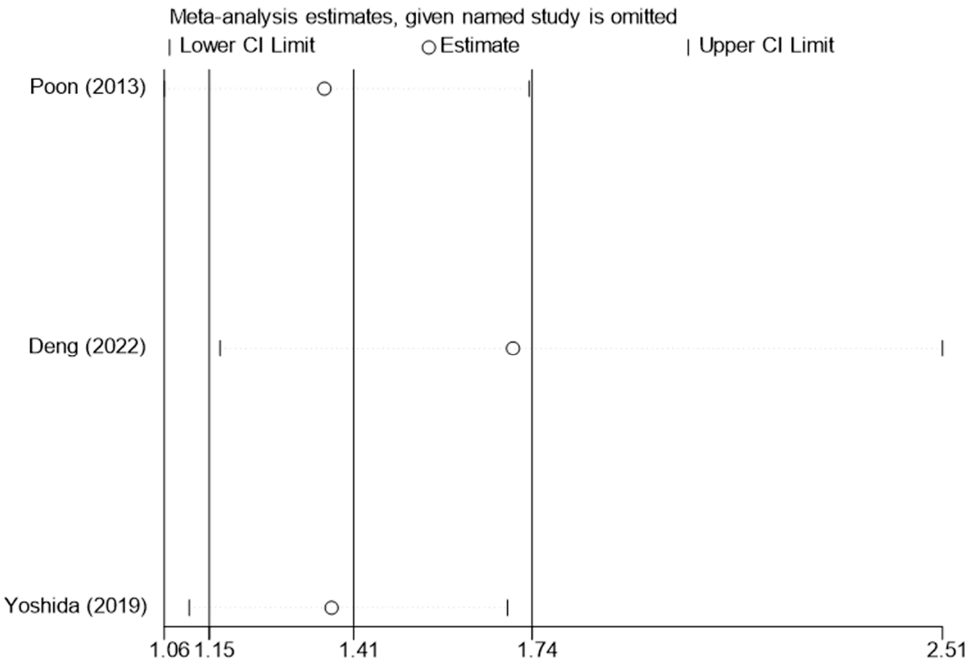

FigureS 66 Sensitivity Analysis of the Association Between Fatigue and PINV

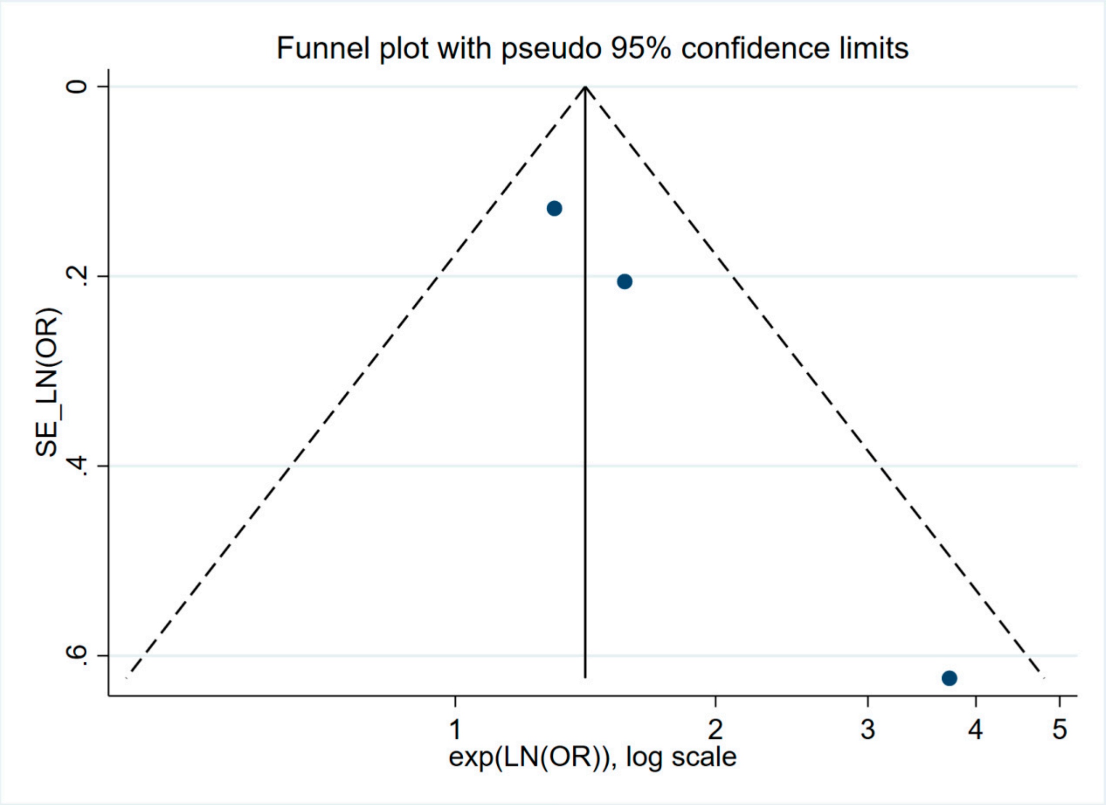

FigureS 67Funnel Plot of Publication Bias in Studies on Fatigue as a Risk Factor for PINV

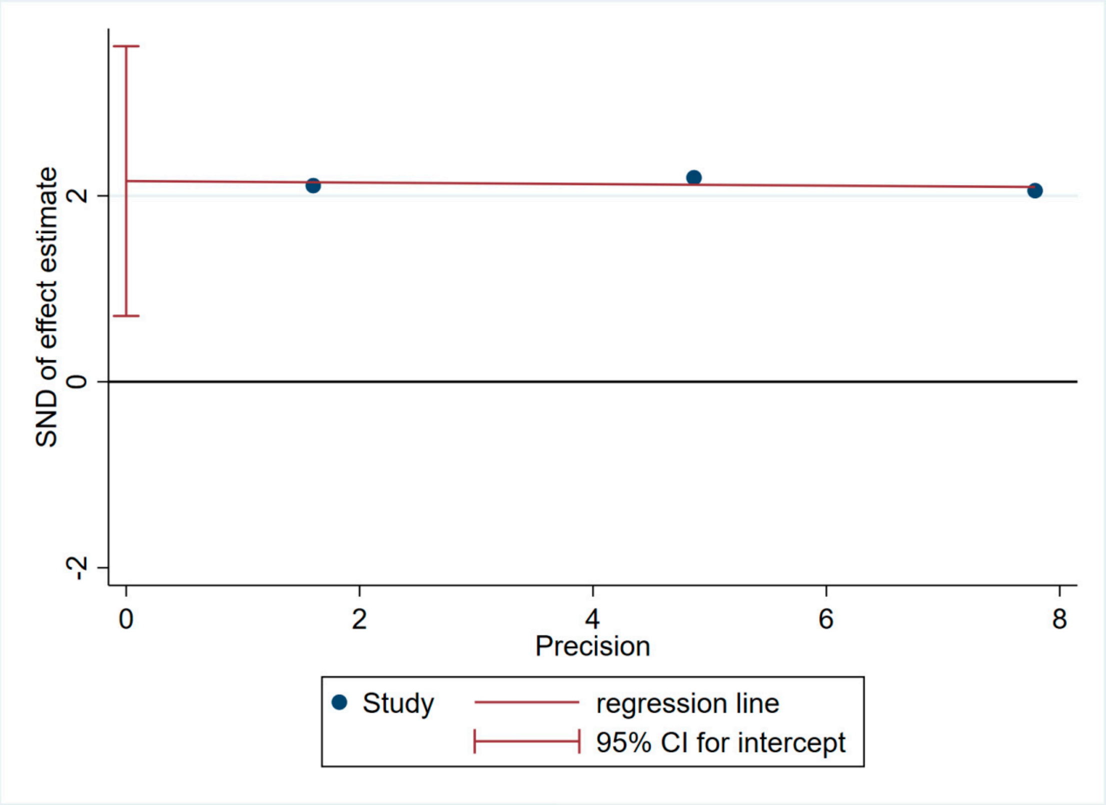

FigureS 68Egger's Test of Publication Bias for Studies Examining Fatigue as a Risk Factor for PINV

```
Regress standard normal deviate of intervention
  effect estimate against its standard error

.
Number of studies =  3                                Root MSE      =   .0928

     Std_Eff | Coefficient  Std. err.      t    P>|t|     [95% conf. interval]
-------------+----------------------------------------------------------------
       slope |  -.0080894   .0212139    -0.38   0.768    -.2776372    .2614585
        bias |   2.158439   .1141821    18.90   0.034     .7076179     3.60926
------------------------------------------------------------------------------

Test of H0: no small-study effects          P = 0.034
```

Motion sickness

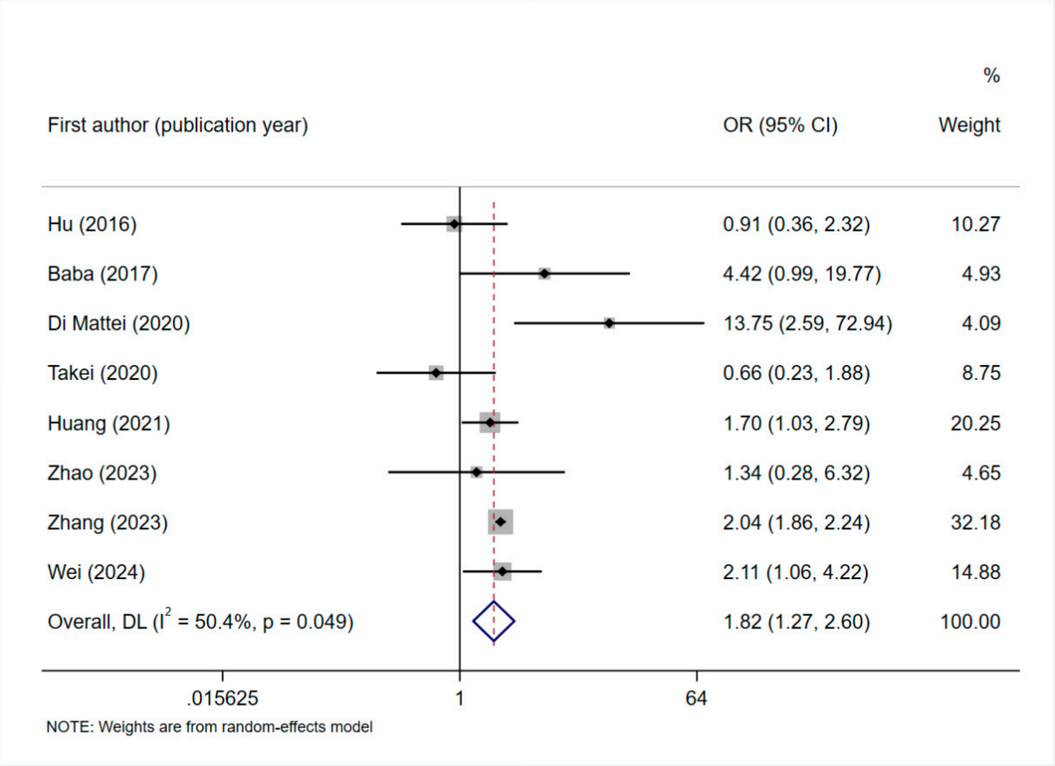

FigureS 69 Forest Plot of the Association Between Motion Sickness History and PINV

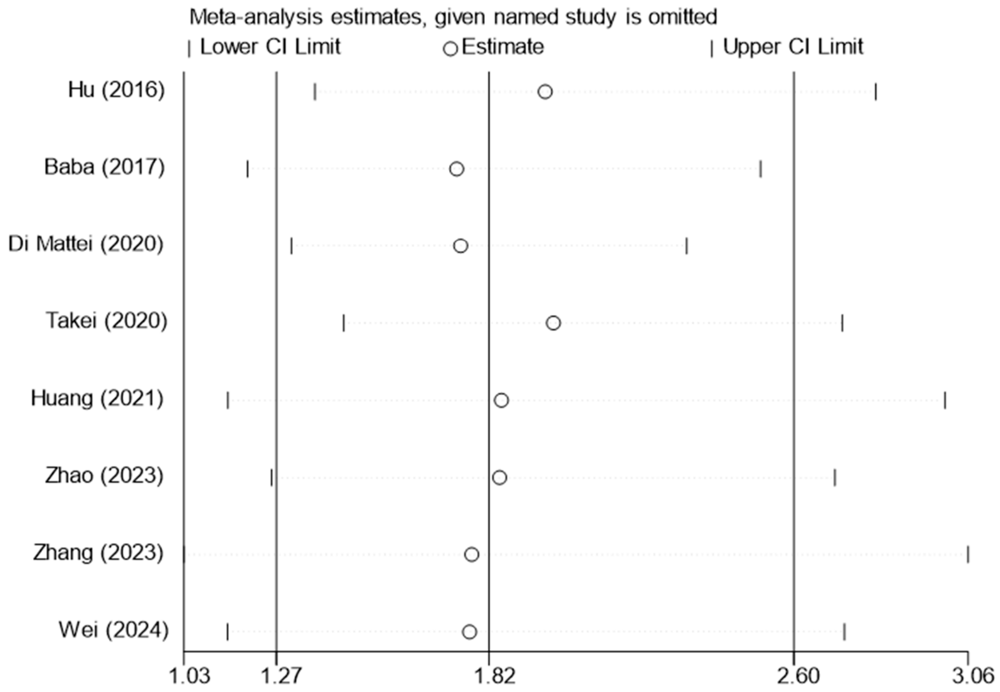

FigureS 70Sensitivity Analysis of the Association Between Motion Sickness History and PINV

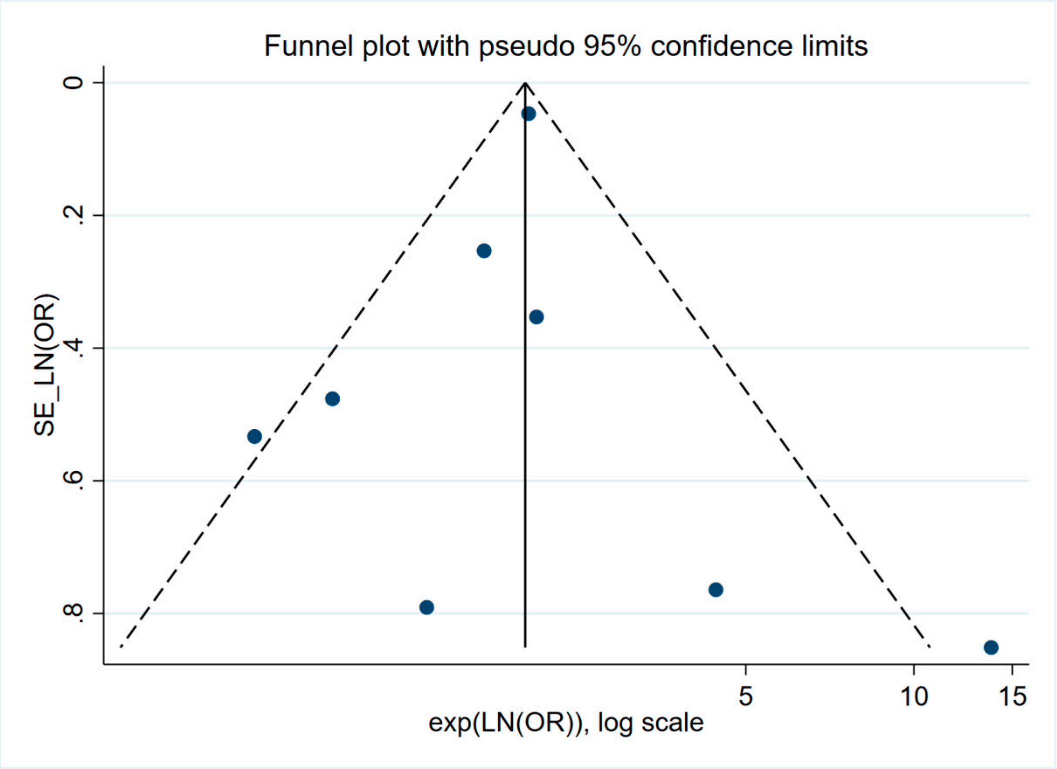

FigureS 71Funnel Plot of Publication Bias in Studies on Motion Sickness as a Risk Factor for PINV

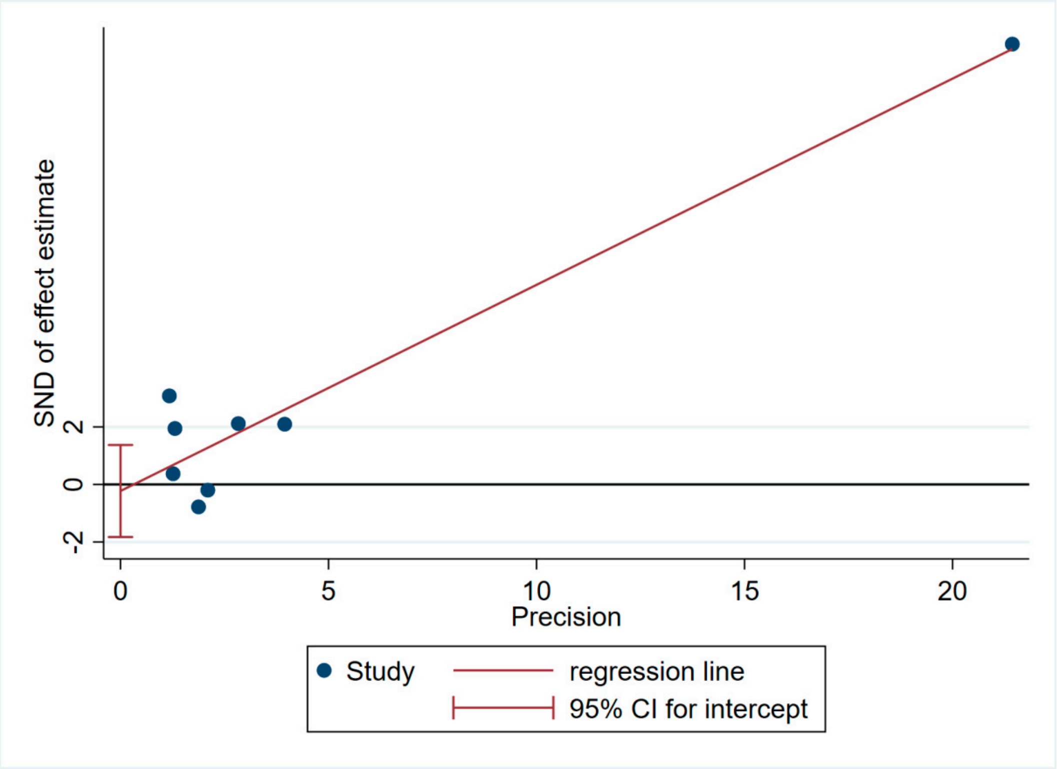

FigureS 72 Egger's Test of Publication Bias for Studies Examining Motion Sickness as a Risk Factor for PINV

```
Regress standard normal deviate of intervention
  effect estimate against its standard error

.
Number of studies =  8                              Root MSE      =   1.519

     Std_Eff | Coefficient  Std. err.      t    P>|t|     [95% conf. interval]
-------------+----------------------------------------------------------------
       slope |   .7166455   .0830727     8.63   0.000      .513374    .9199171
        bias |  -.2278823   .6539653    -0.35   0.739    -1.828078    1.372313

Test of H0: no small-study effects          P = 0.739
```

As the heterogeneity was within an acceptable range, no further subgroup analysis was conducted.

Performance status

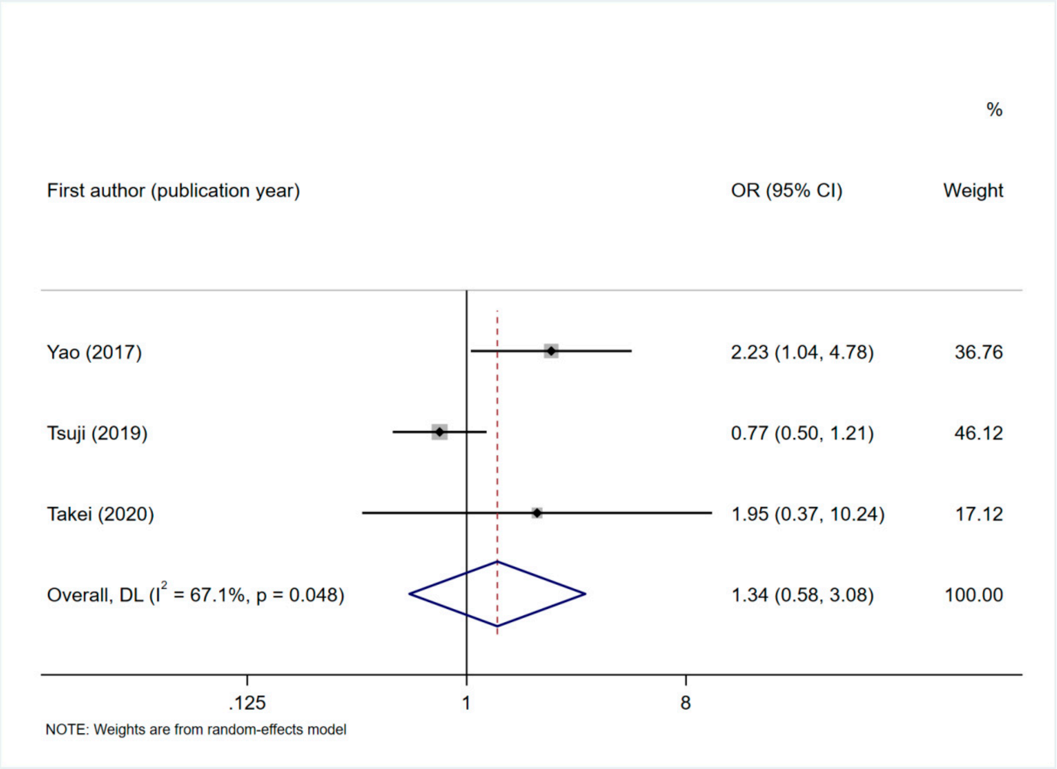

FigureS 73 Forest Plot of the Association Between Performance Status and PINV

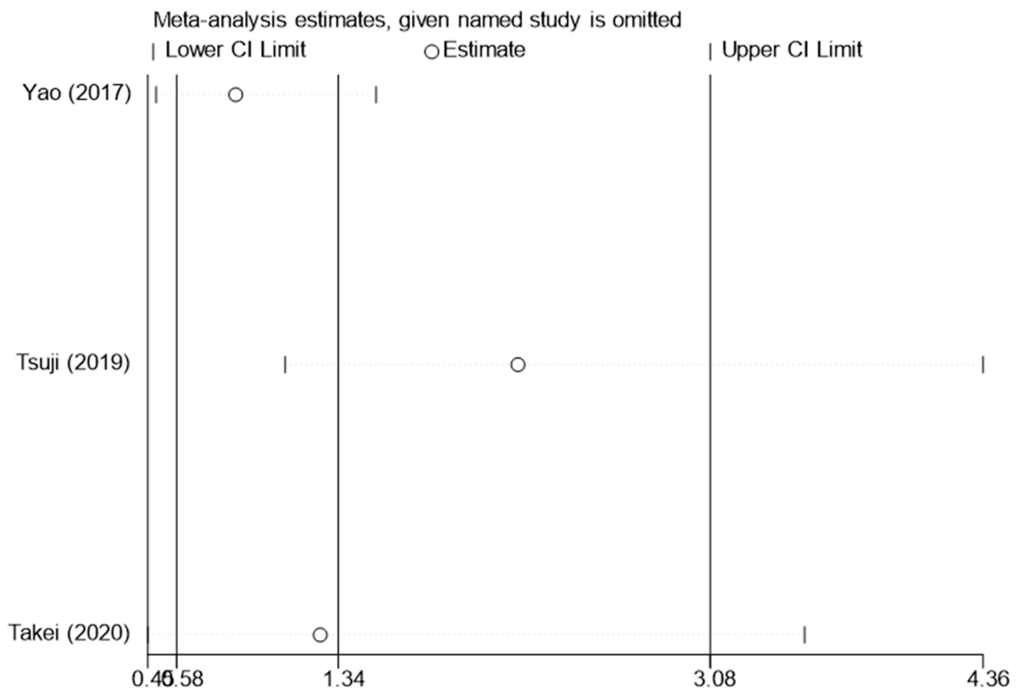

FigureS 74Sensitivity Analysis of the Impact of Performance Status on PINV

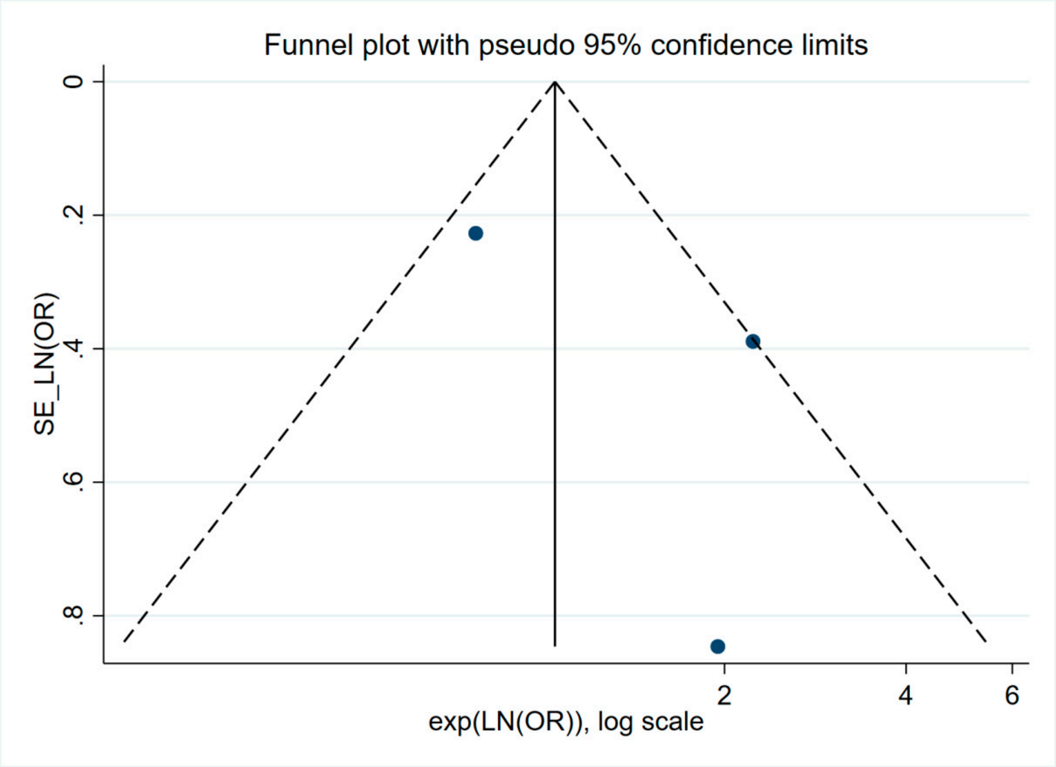

FigureS 75 Funnel Plot of Publication Bias in Studies Examining Performance Status as a Predictor of PINV

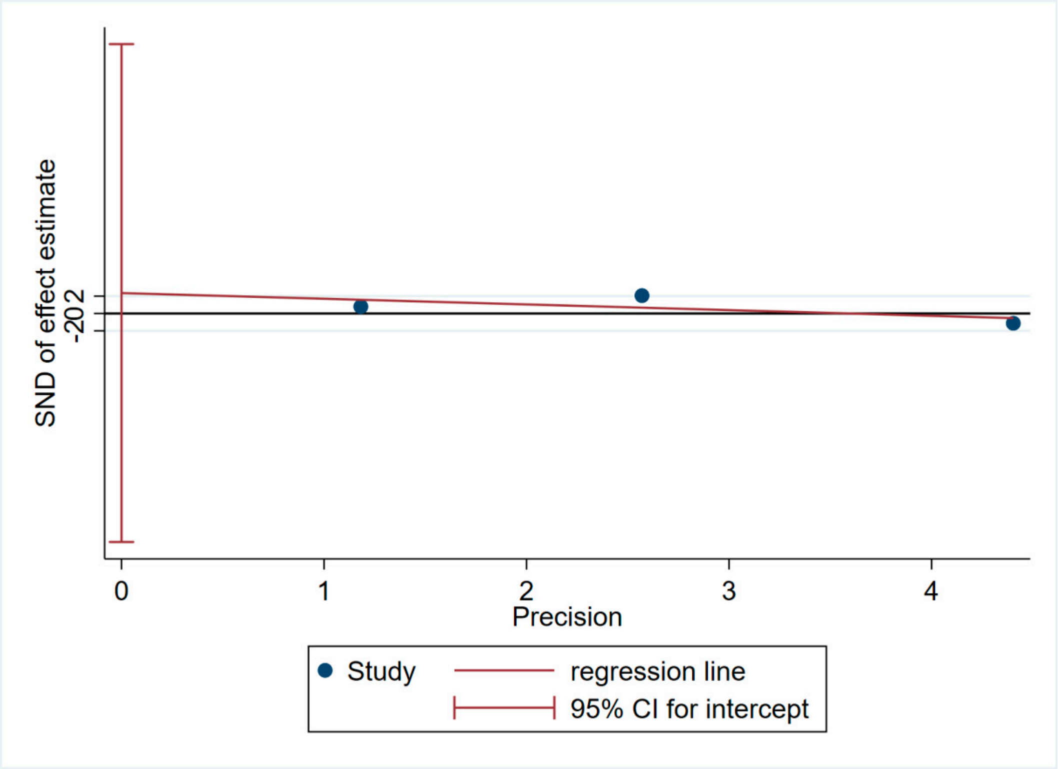

FigureS 76Egger’ s Test of Publication Bias for Studies Assessing Performance Status and PINV

```
Egger's test for small-study effects:
Regress standard normal deviate of intervention
  effect estimate against its standard error

.
Number of studies =  3                                 Root MSE      =   1.708
------------------------------------------------------------------------------
     Std_Eff | Coefficient  Std. err.      t    P>|t|     [95% conf. interval]
-------------+----------------------------------------------------------------
       slope |  -.6547962   .7471944   -0.88   0.542     -10.1488    8.839209
        bias |   2.354261   2.257949    1.04   0.487     -26.3357    31.04422
------------------------------------------------------------------------------

Test of H0: no small-study effects          P = 0.487
```

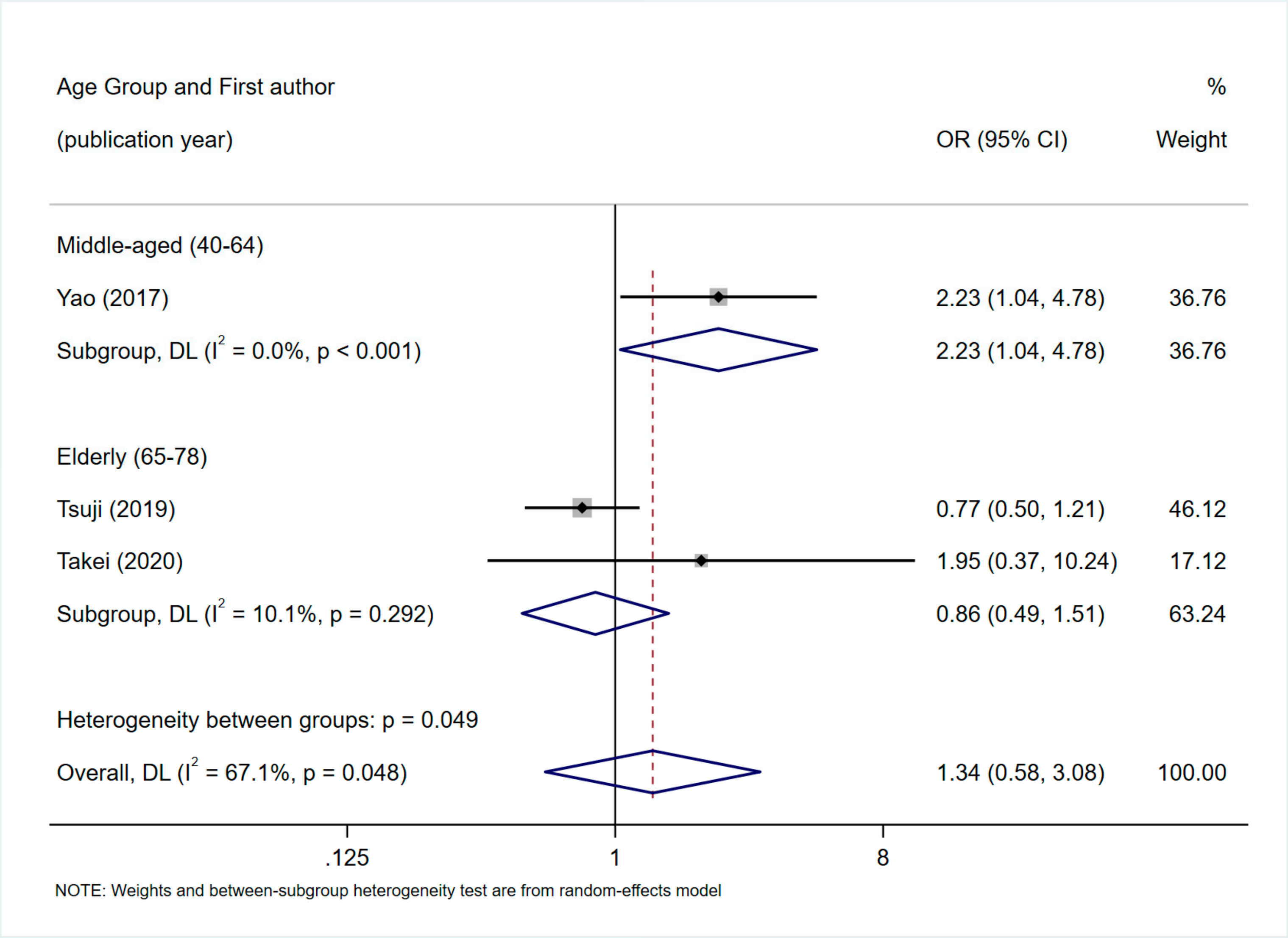
Age Group and First author
(publication year)
OR (95% CI)
%
Weight
Middle-aged (40-64)
Yao (2017)
2.23 (1.04, 4.78)
36.76
Subgroup, DL (I^2 = 0.0%, p < 0.001)
2.23 (1.04, 4.78)
36.76
Elderly (65-78)
Tsuji (2019)
0.77 (0.50, 1.21)
46.12
Takei (2020)
1.95 (0.37, 10.24)
17.12
Subgroup, DL (I^2 = 10.1%, p = 0.292)
0.86 (0.49, 1.51)
63.24
Heterogeneity between groups: p = 0.049
Overall, DL (I^2 = 67.1%, p = 0.048)
1.34 (0.58, 3.08)
100.00
.125
1
8
NOTE: Weights and between-subgroup heterogeneity test are from random-effects model

FigureS 77Forest Plot of Subgroup Analysis by Age Group for the Association Between Performance status and PINV

Age Group as a Source of Heterogeneity:

To explore potential sources of heterogeneity, we conducted a subgroup analysis stratified by age group, as illustrated in the forest plot. The effect estimates varied notably between middle-aged and elderly patients:

Middle-aged group (40–64 years): OR = 2.23 (95% CI: 1.04–4.78), contributing 36.76% of the total weight.

Elderly group (65–78 years): OR = 0.86 (95% CI: 0.49–1.51), contributing 63.24% of the total weight.

The test for subgroup differences showed statistical significance ($p = 0.049$), suggesting that age group may be a meaningful source of heterogeneity in the association between performance status and PINV. Specifically, poorer performance status appeared to confer a significantly higher risk of PINV among middle-aged patients, whereas no such association was observed in the elderly. This discrepancy may reflect age-related differences in functional reserve, treatment tolerance, symptom reporting, or clinician thresholds for supportive care interventions.

Courses of chemotherapy

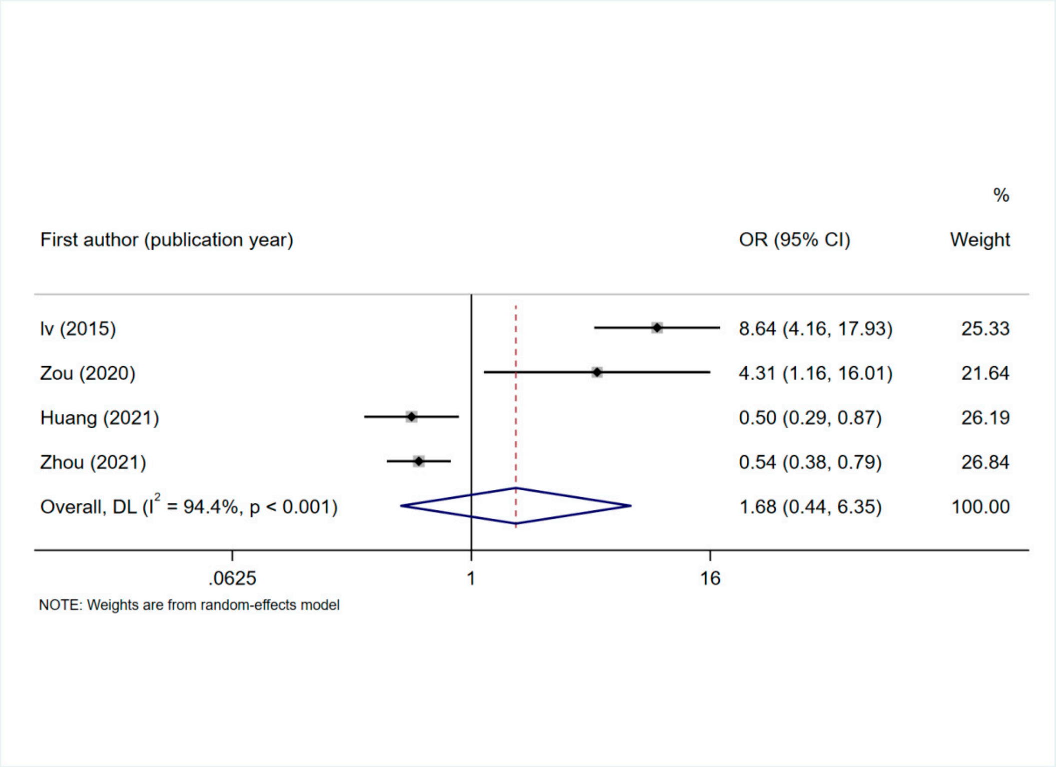

FigureS 78Forest Plot of the Association Between Courses of Chemotherapy and PINV

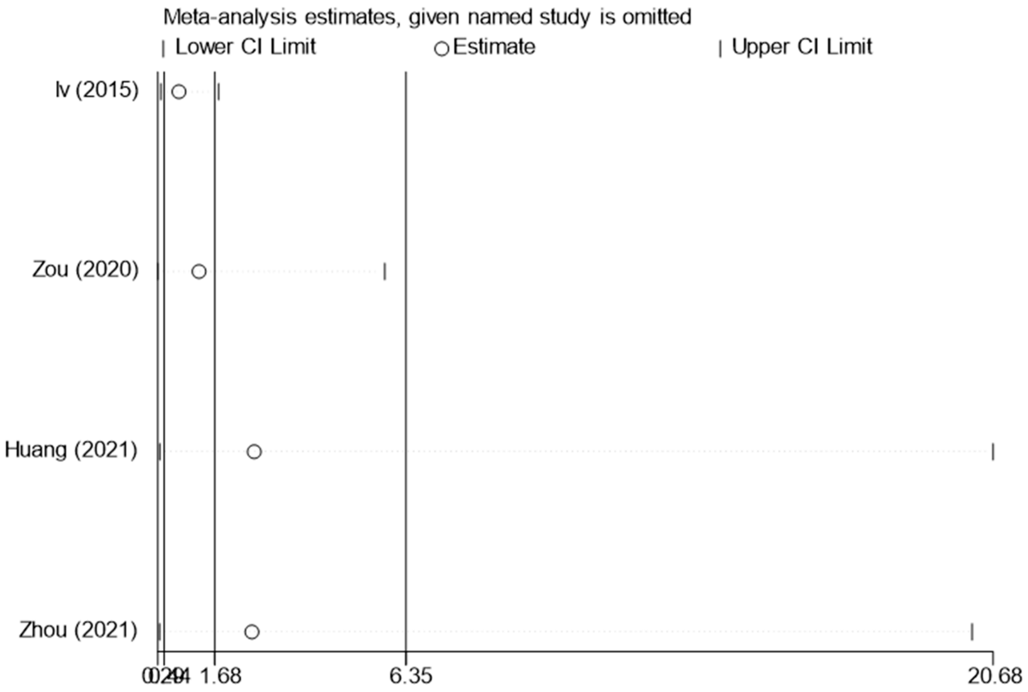

FigureS 79Sensitivity Analysis of the Impact of Courses of Chemotherapy on PINV

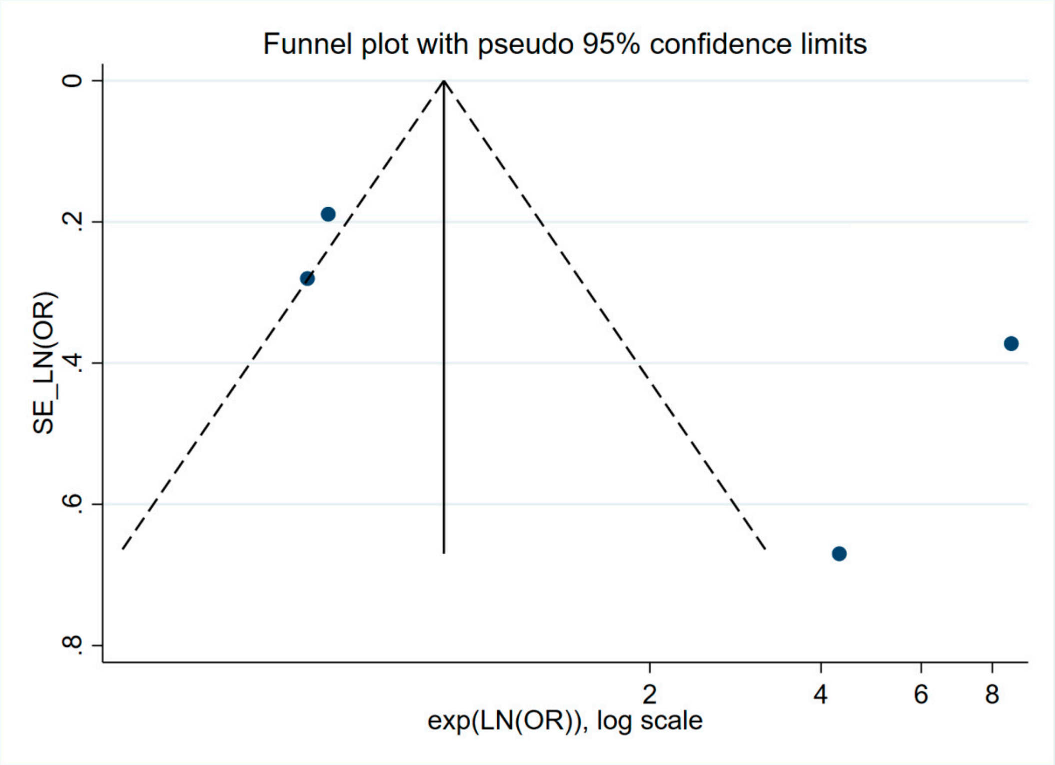

FigureS 80 Funnel Plot of Publication Bias in Studies Examining the Association Between Courses of Chemotherapy and PINV

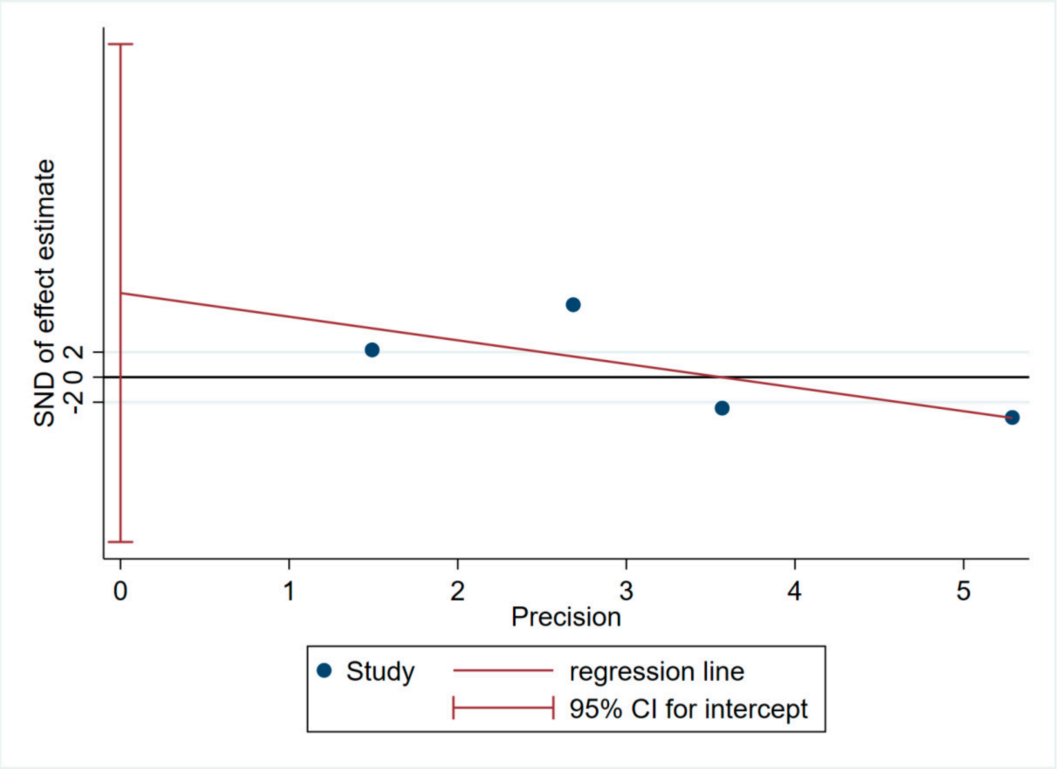

FigureS 81Egger’ s Test of Publication Bias for Studies Assessing Courses of Chemotherapy and PINV

[illegible]

```
Regress standard normal deviate of intervention
  effect estimate against its standard error

.
Number of studies =  4                              Root MSE      =   3.615
------------------------------------------------------------------------------
     Std_Eff | Coefficient  Std. err.      t    P>|t|     [95% conf. interval]
-------------+----------------------------------------------------------------
       slope |  -1.887839    1.30577    -1.45   0.285    -7.506113    3.730436
        bias |   6.720772   4.623095     1.45   0.283     -13.1708    26.61235
------------------------------------------------------------------------------

Test of H0: no small-study effects          P = 0.283
```

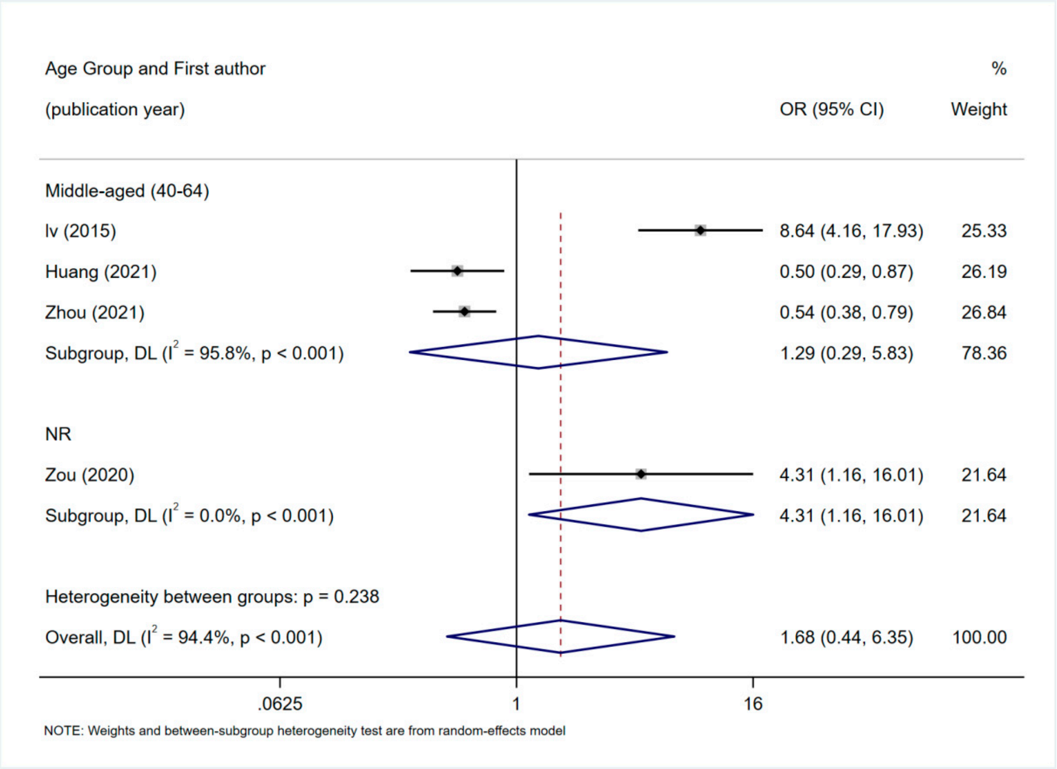

FigureS 82Forest Plot of Subgroup Analysis by Age Group for the Association Between Courses of Chemotherapy and PINV

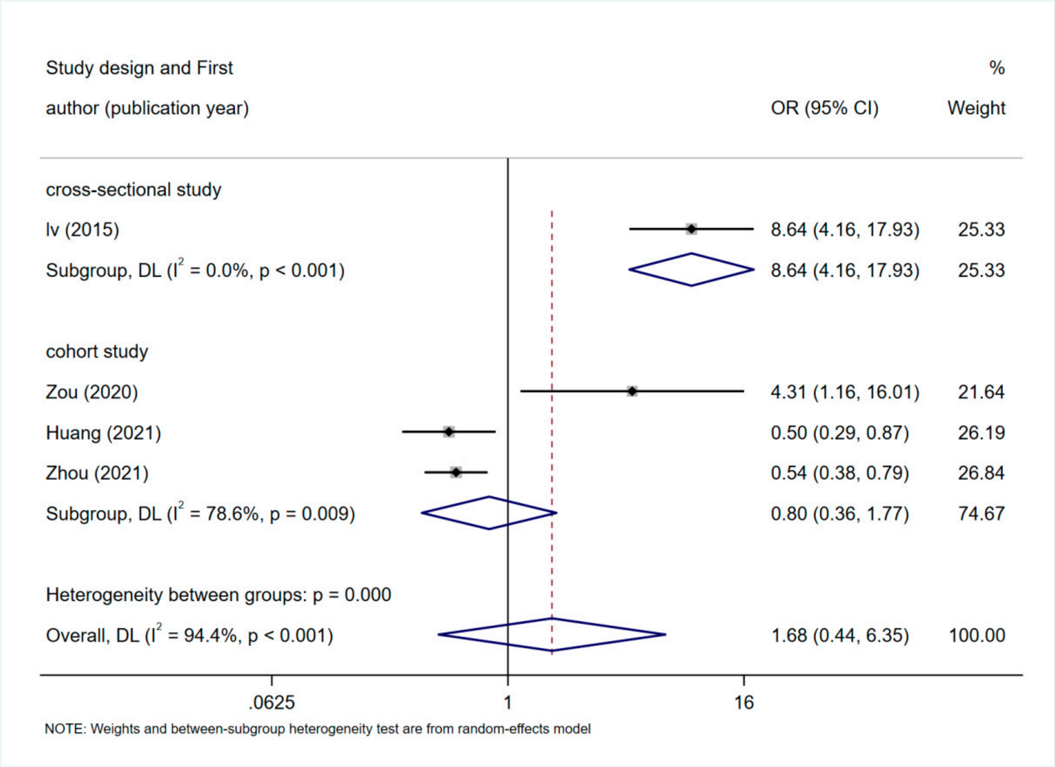

FigureS 83Forest Plot of Subgroup Analysis by Study Design for the Association Between Courses of Chemotherapy and PINV

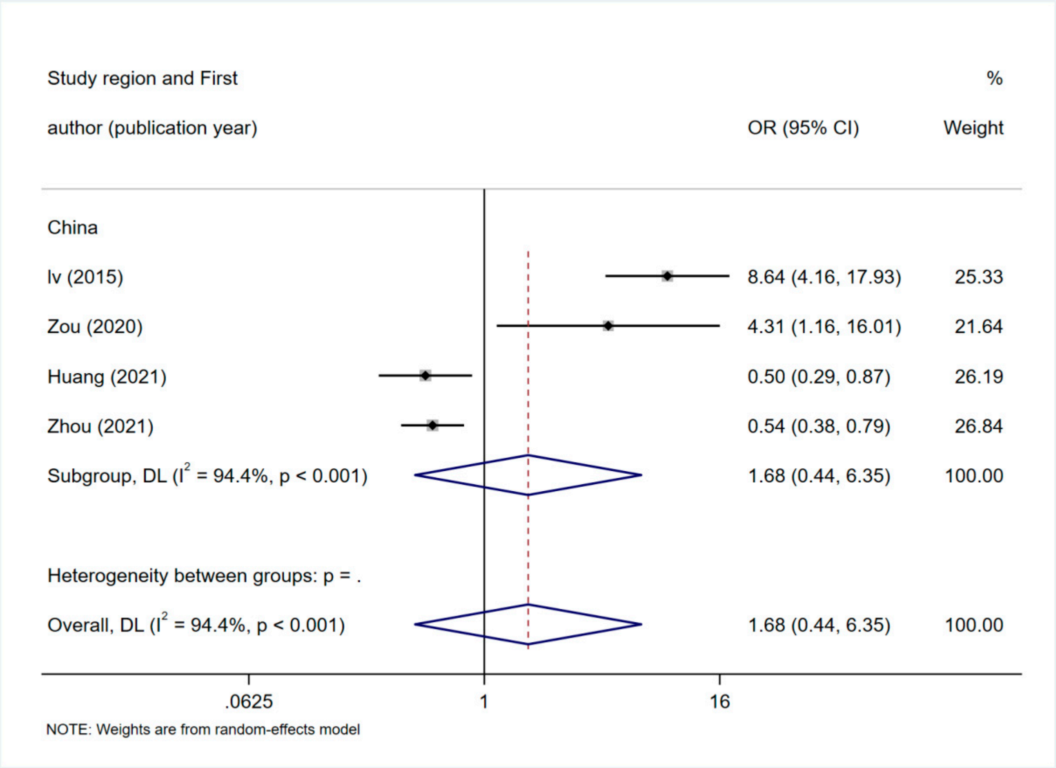

FigureS 84Forest Plot of Subgroup Analysis by Geographical Region for the Association Between Courses of Chemotherapy and PINV

Sources of Heterogeneity：

Despite extensive efforts, including sensitivity analysis, meta-regression, and subgroup analysis, the source of heterogeneity remained unexplained, potentially due to unmeasured confounders or random variation.

Number of chemotherapy sessions

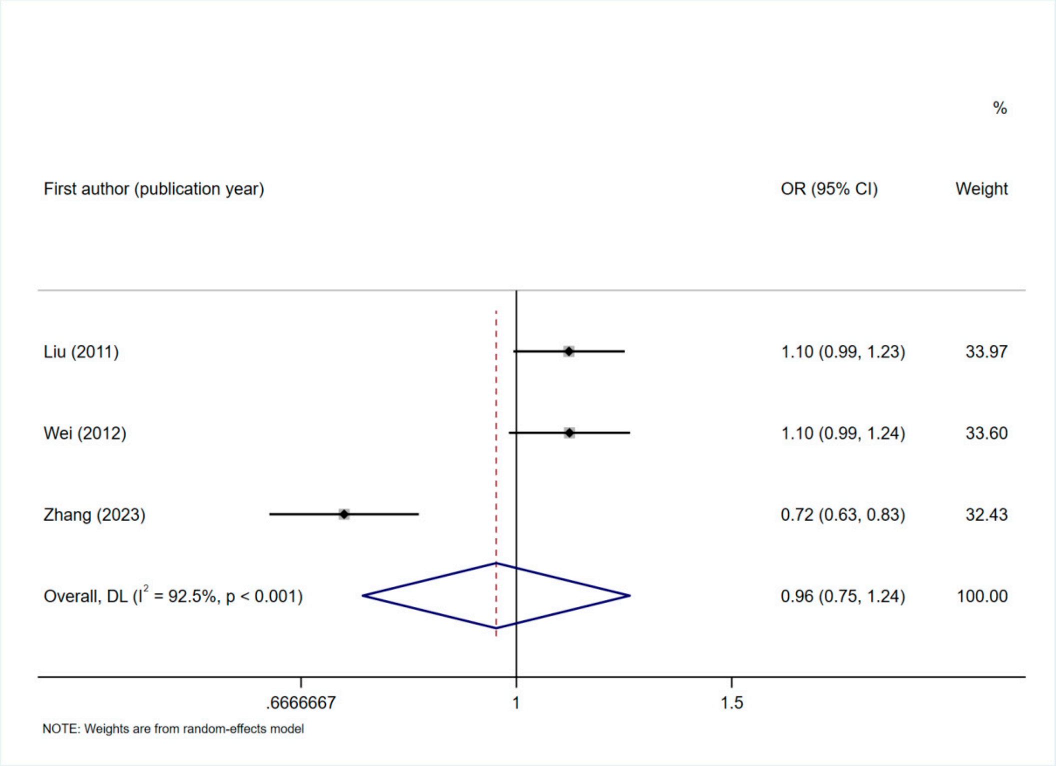

FigureS 85Forest Plot of the Association Between Number of Chemotherapy Sessions and PINV

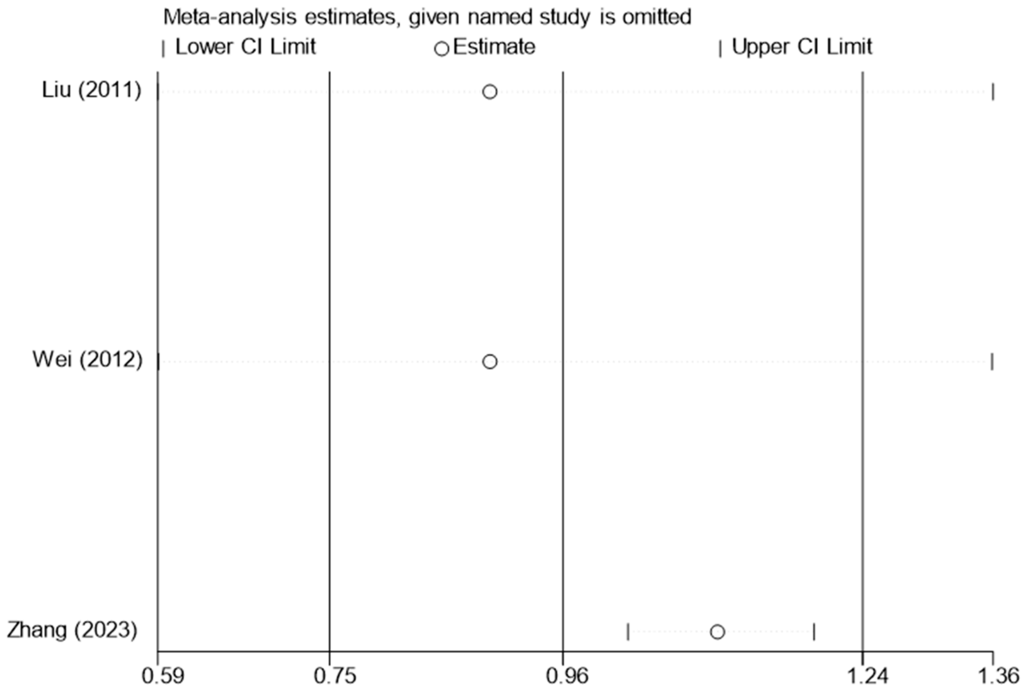

FigureS 86Sensitivity Analysis of the Impact of Number of Chemotherapy Sessions on PINV

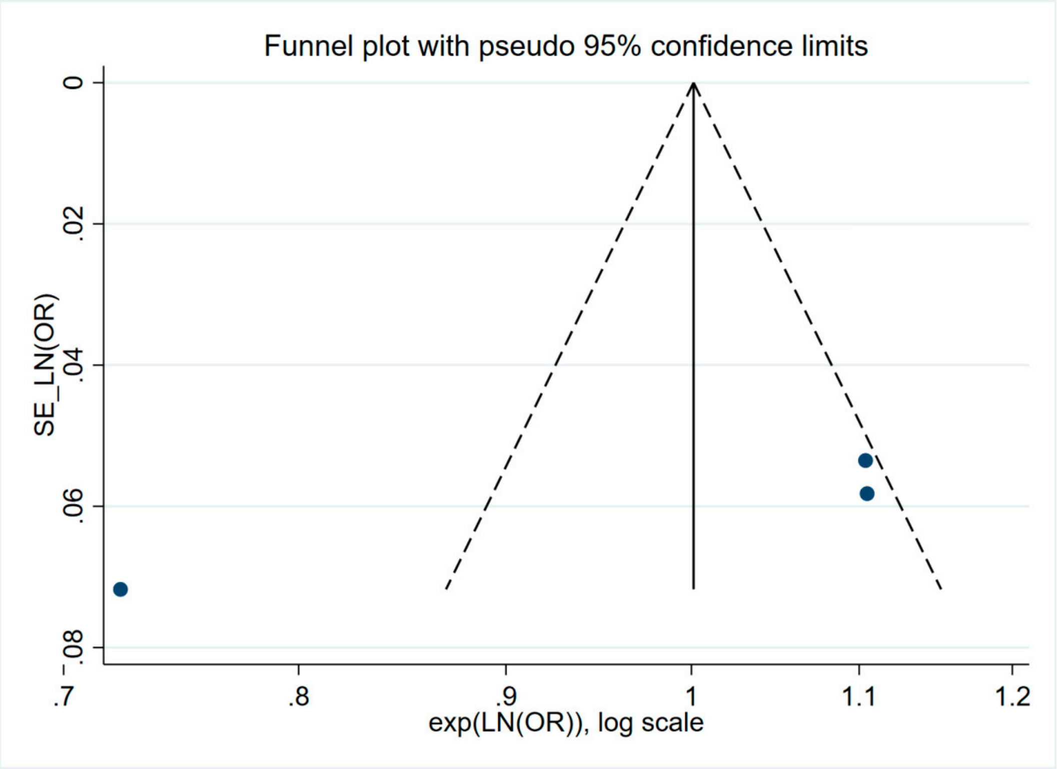

FigureS 87Funnel Plot of Publication Bias in Studies Examining the Association Between Number of Chemotherapy Sessions and PINV

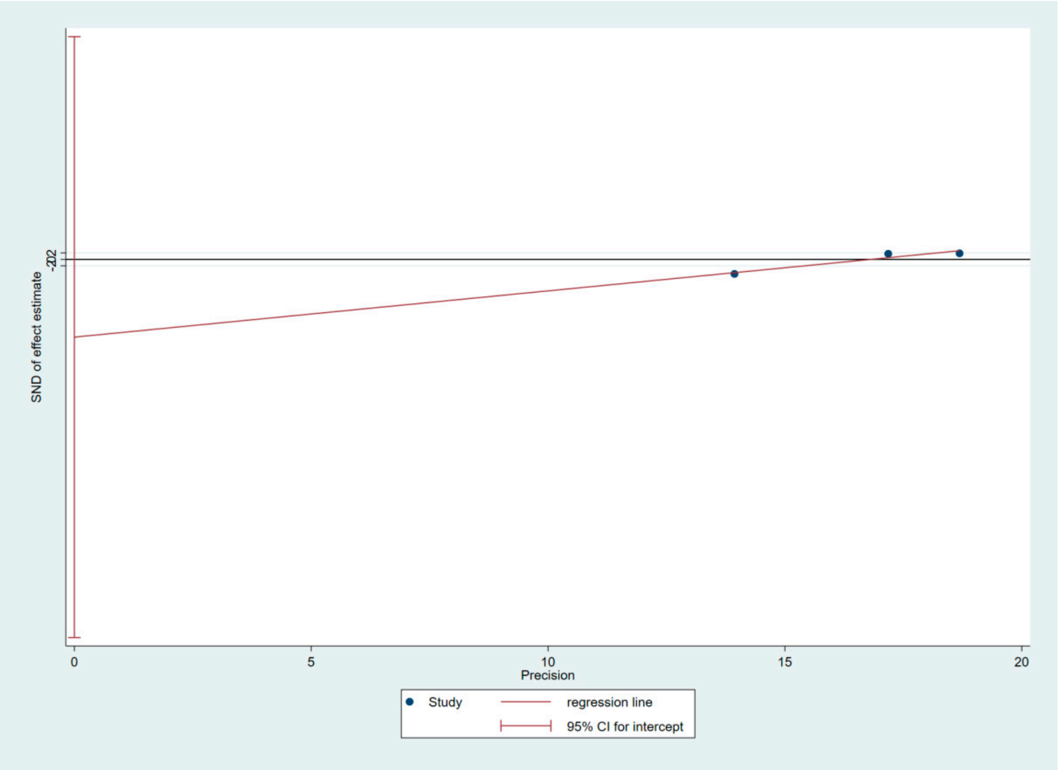
SND of effect estimate
-202
0
5
10
15
20
Precision
Study
regression line
95% CI for intercept

Regress standard normal deviate of intervention
effect estimate against its standard error

.
Number of studies = 3   Root MSE = 1.505

Std_Eff	Coefficient	Std. err.	t	P>|t|	[95% conf. interval]	
slope	1.433109	.4383844	3.27	0.189	-4.137093	7.003311
bias	-24.10967	7.329373	-3.29	0.188	-117.2382	69.01884

Test of H0: no small-study effects   P = 0.188

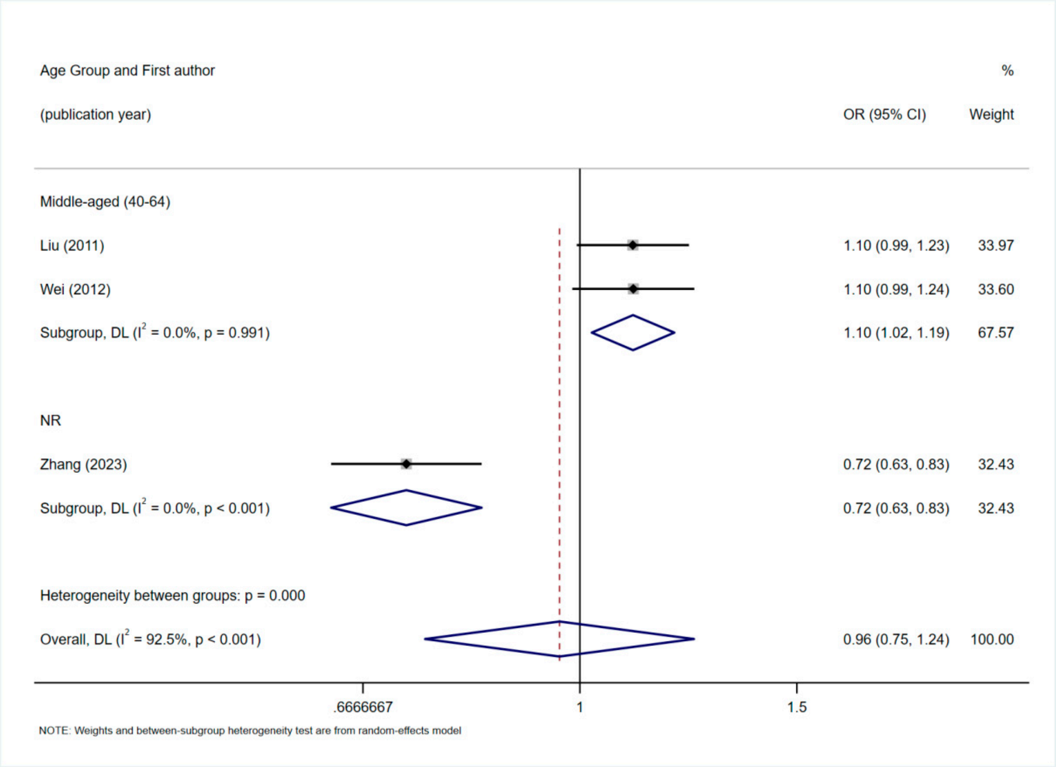

FigureS 88 Forest Plot of Subgroup Analysis by Age Group for the Association Between Number of Chemotherapy Sessions and PINV

Sources of Heterogeneity：

Age Group as a Source of Heterogeneity

A subgroup analysis by age group was conducted to explore heterogeneity in the association between the number of chemotherapy sessions and PINV. Among middle-aged patients (40–64 years), the pooled OR was 1.10 (95% CI: 1.02–1.19), while in the NR group (Zhang 2023), the OR was 0.72 (95% CI: 0.63–0.83). No within-

group heterogeneity was observed ($I^2 = 0.0\%$ for both). However, between-group heterogeneity was significant ($p = 0.000$), suggesting age group as a potential source of variation.

History of vomiting during chemotherapy

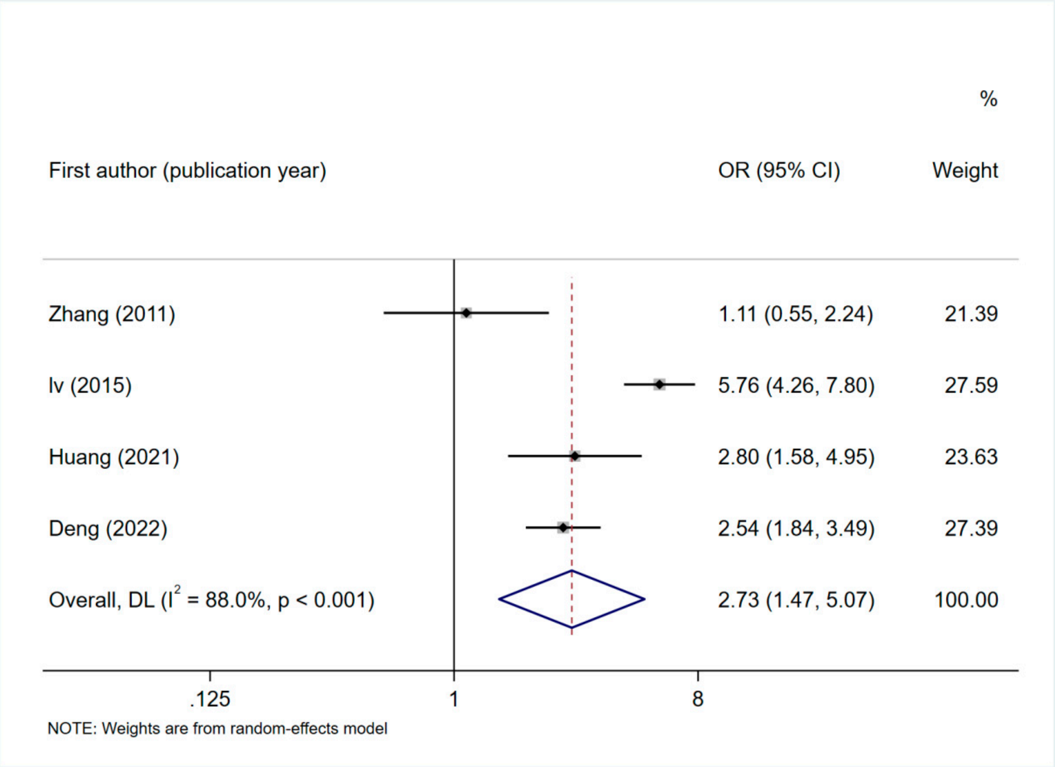

FigureS 89Forest Plot of the Association Between History of vomiting and PINV

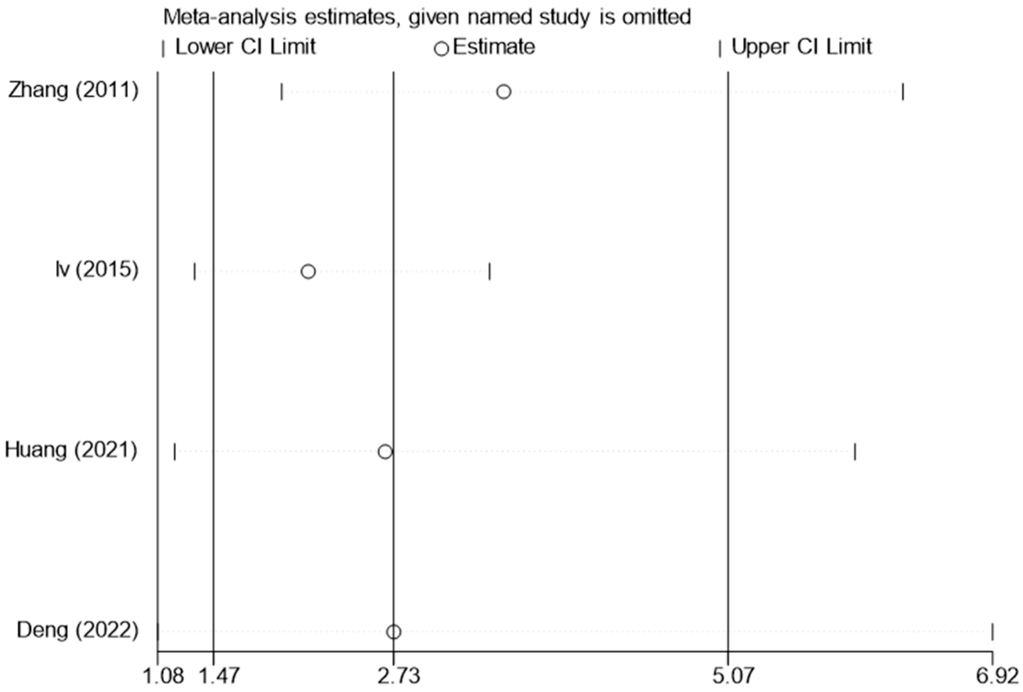

FigureS 90Sensitivity Analysis of the Impact of History of vomiting on PINV

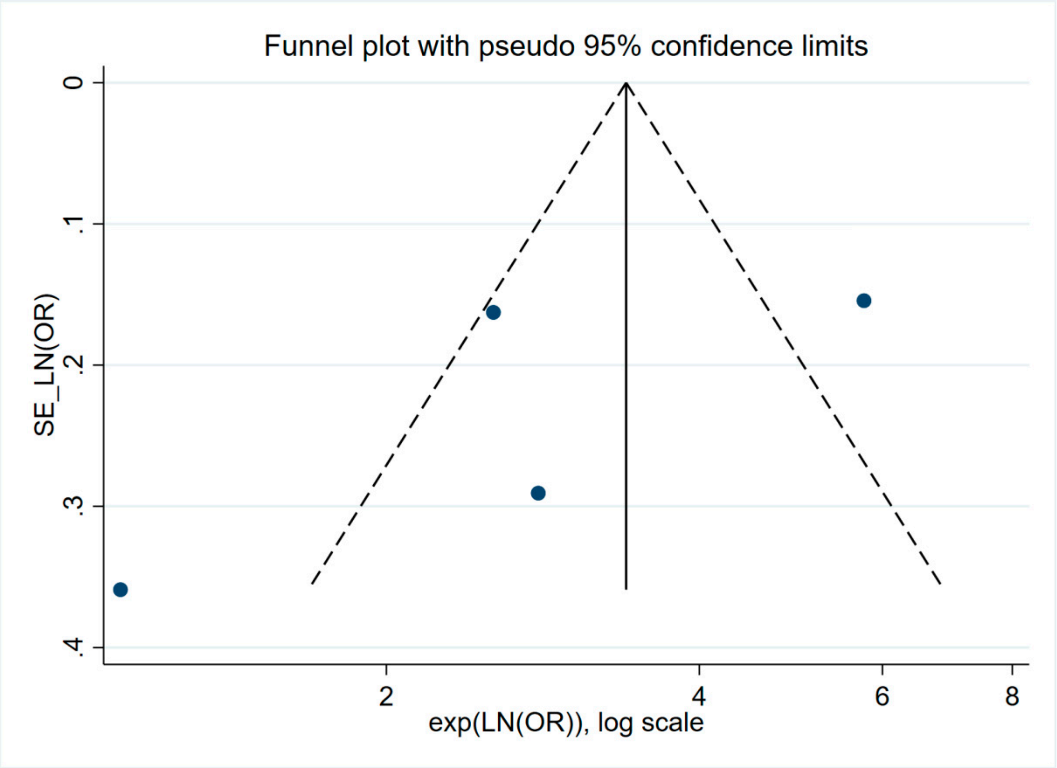

FigureS 91Funnel Plot of Publication Bias in Studies Examining the Association Between History of vomiting and PINV

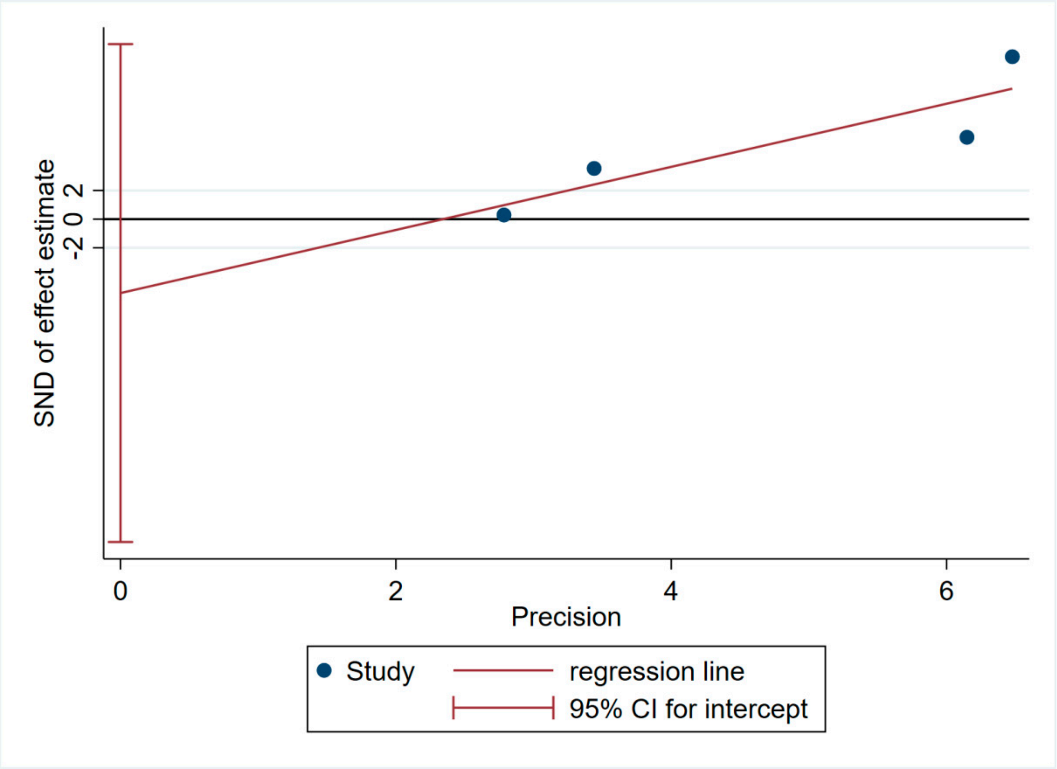

FigureS 92Egger’ s Test of Publication Bias for Studies Assessing History of vomiting and PINV

```
ger's test for small-study effects:
gress standard normal deviate of intervention
effect estimate against its standard error

mber of studies =  4                                Root MSE      =   2.628
------------------------------------------------------------------------------
     Std_Eff | Coefficient  Std. err.      t    P>|t|     [95% conf. interval]
-------------+----------------------------------------------------------------
       slope |   2.203591   .8107531     2.72   0.113    -1.284798    5.691979
        bias |  -5.161482   4.040397    -1.28   0.330    -22.54591    12.22294
------------------------------------------------------------------------------

st of H0: no small-study effects          P = 0.330
```

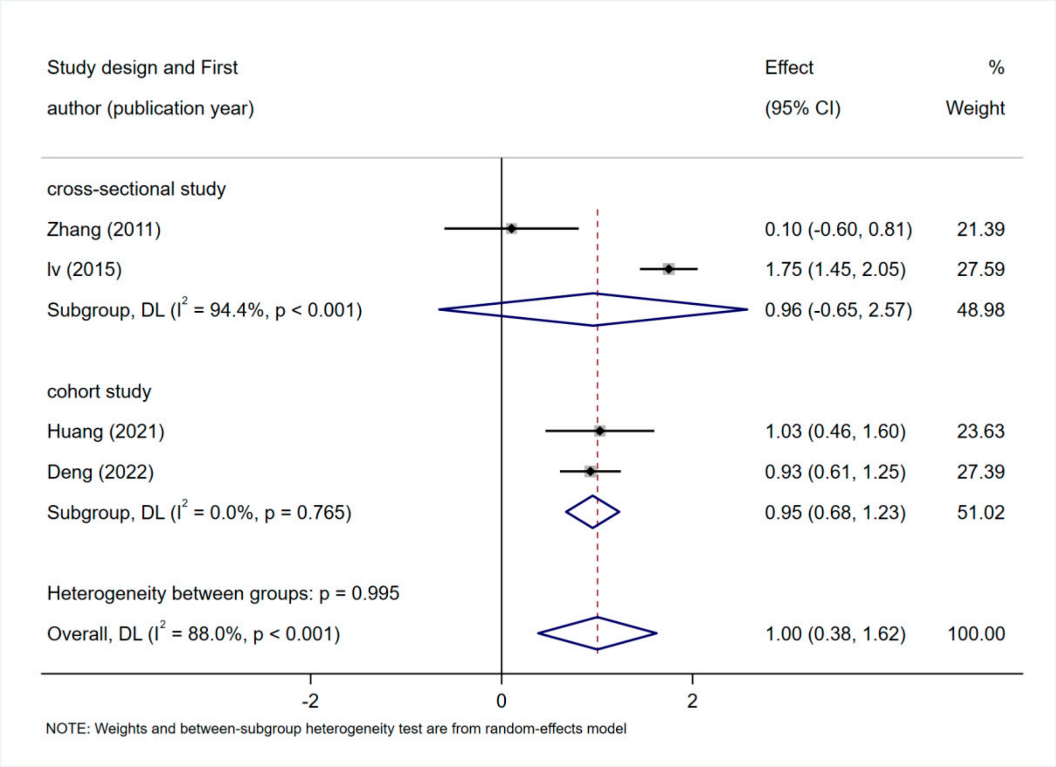

FigureS 93Forest Plot of Subgroup Analysis by Study design for the Association Between History of vomiting and PINV

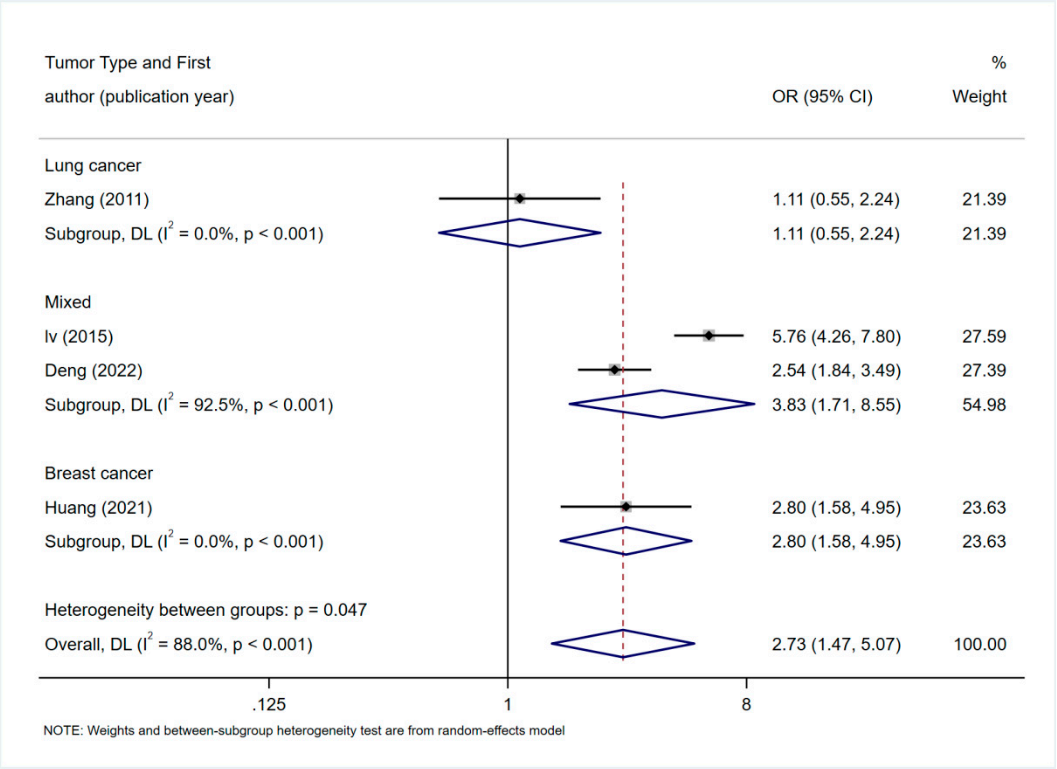

FigureS 94Forest Plot of Subgroup Analysis by Tumor Type for the Association Between History of vomiting and PINV

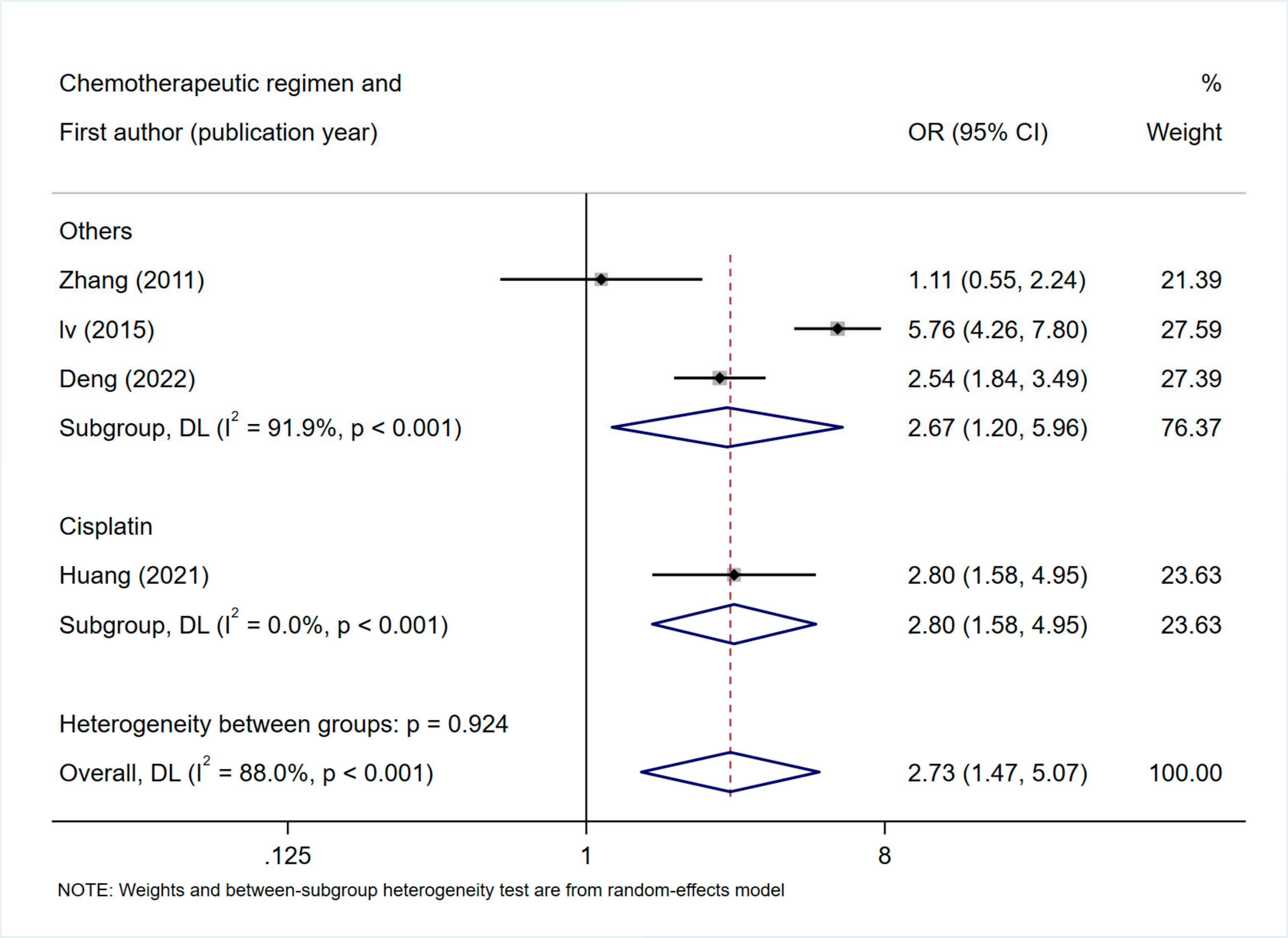
Chemotherapeutic regimen and
First author (publication year)
OR (95% CI)
% Weight
Others
Zhang (2011)
1.11 (0.55, 2.24)
21.39
lv (2015)
5.76 (4.26, 7.80)
27.59
Deng (2022)
2.54 (1.84, 3.49)
27.39
Subgroup, DL (I^2 = 91.9%, p < 0.001)
2.67 (1.20, 5.96)
76.37
Cisplatin
Huang (2021)
2.80 (1.58, 4.95)
23.63
Subgroup, DL (I^2 = 0.0%, p < 0.001)
2.80 (1.58, 4.95)
23.63
Heterogeneity between groups: p = 0.924
Overall, DL (I^2 = 88.0%, p < 0.001)
2.73 (1.47, 5.07)
100.00
.125
1
8
NOTE: Weights and between-subgroup heterogeneity test are from random-effects model

FigureS 95Forest Plot of Subgroup Analysis by Chemotherapeutic regimen for the Association Between History of vomiting and PINV

Sources of Heterogeneity：

Despite extensive efforts, including sensitivity analysis, meta-regression, and subgroup analysis, the source of heterogeneity remained unexplained, potentially due to unmeasured confounders or random variation.

Chemotherapy regimen

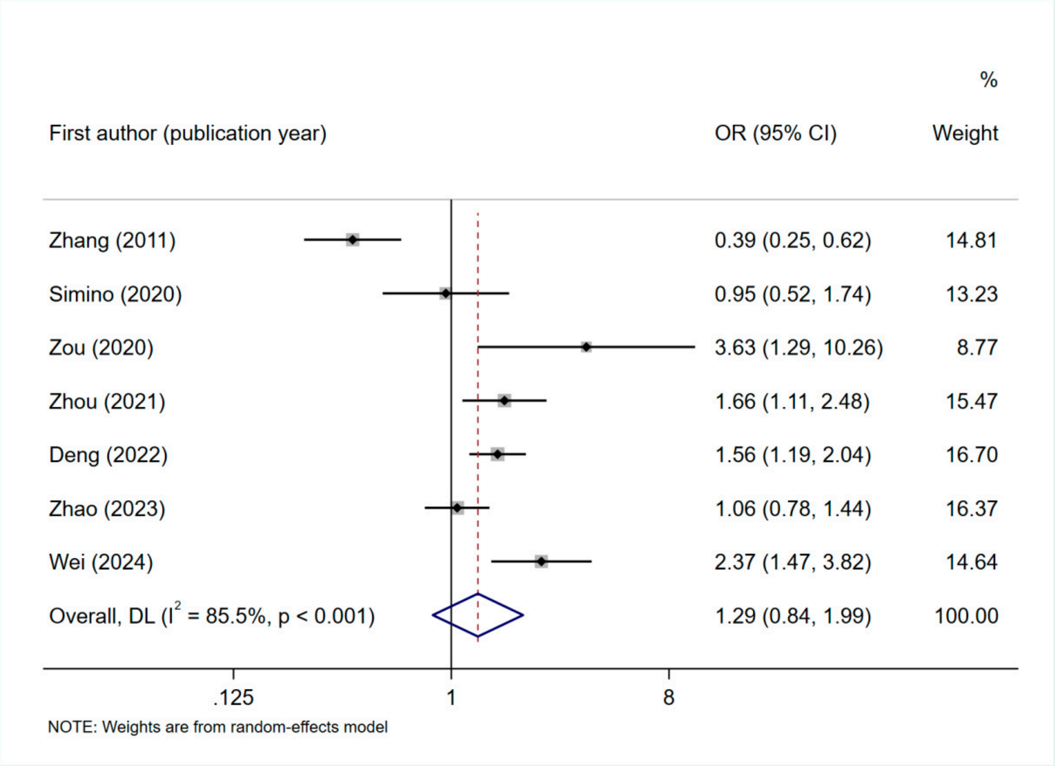

FigureS 96Forest Plot of the Association Between Chemotherapy Regimen and PINV

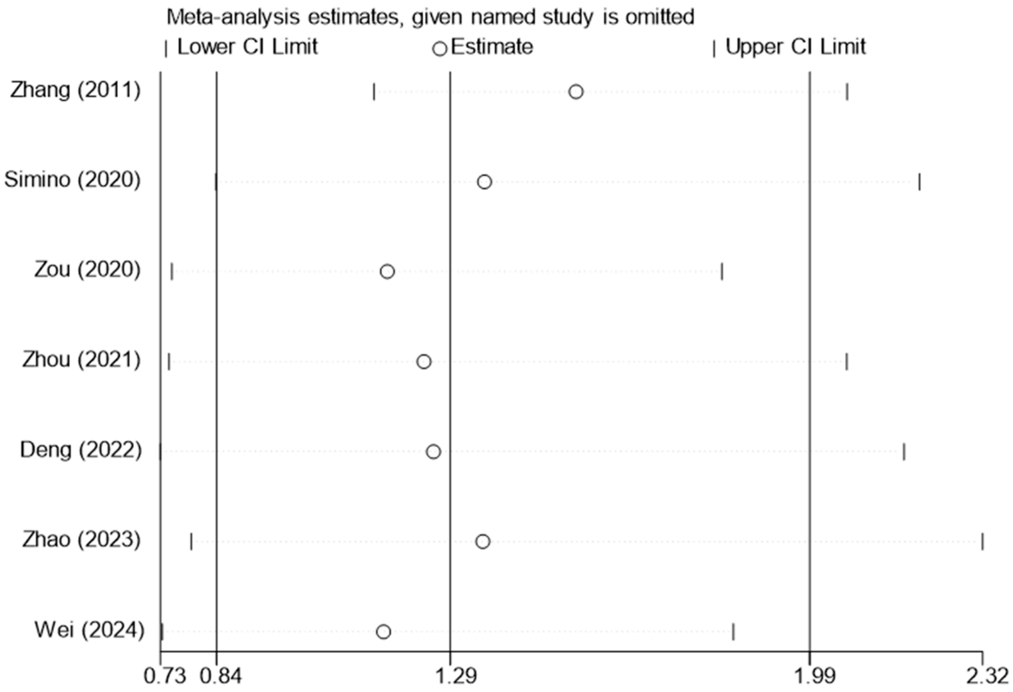

FigureS 97Sensitivity Analysis of the Impact of Chemotherapy Regimen on PINV

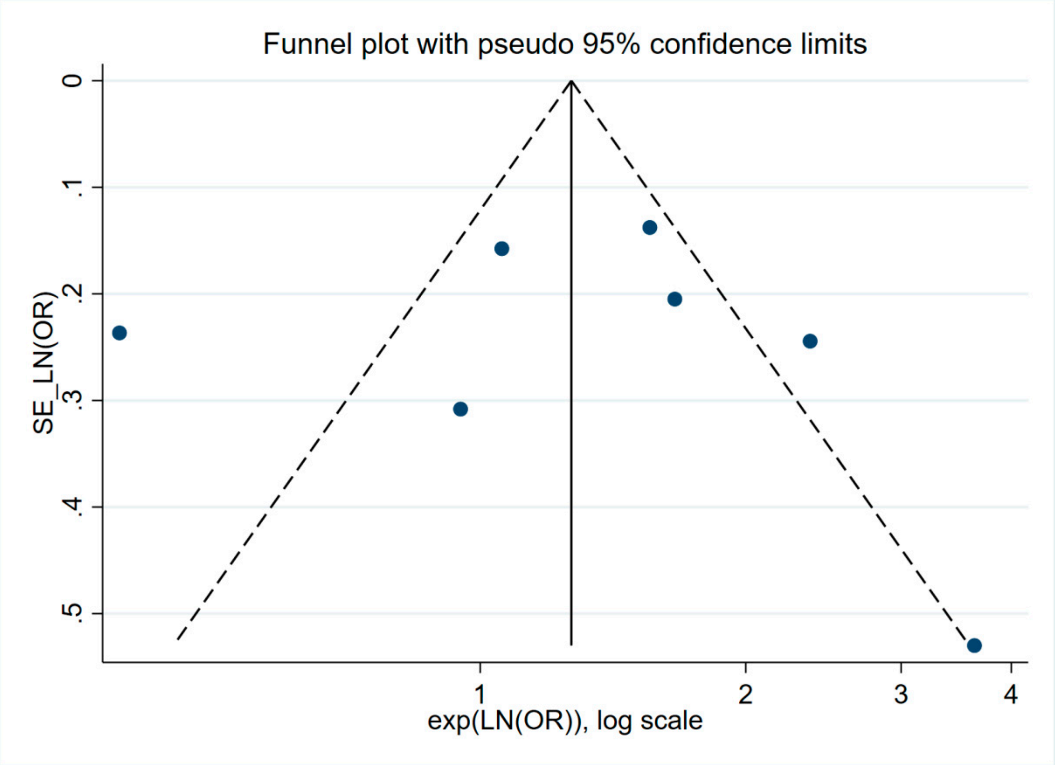

FigureS 98Funnel Plot of Publication Bias in Studies Examining the Association Between Chemotherapy Regimen and PINV

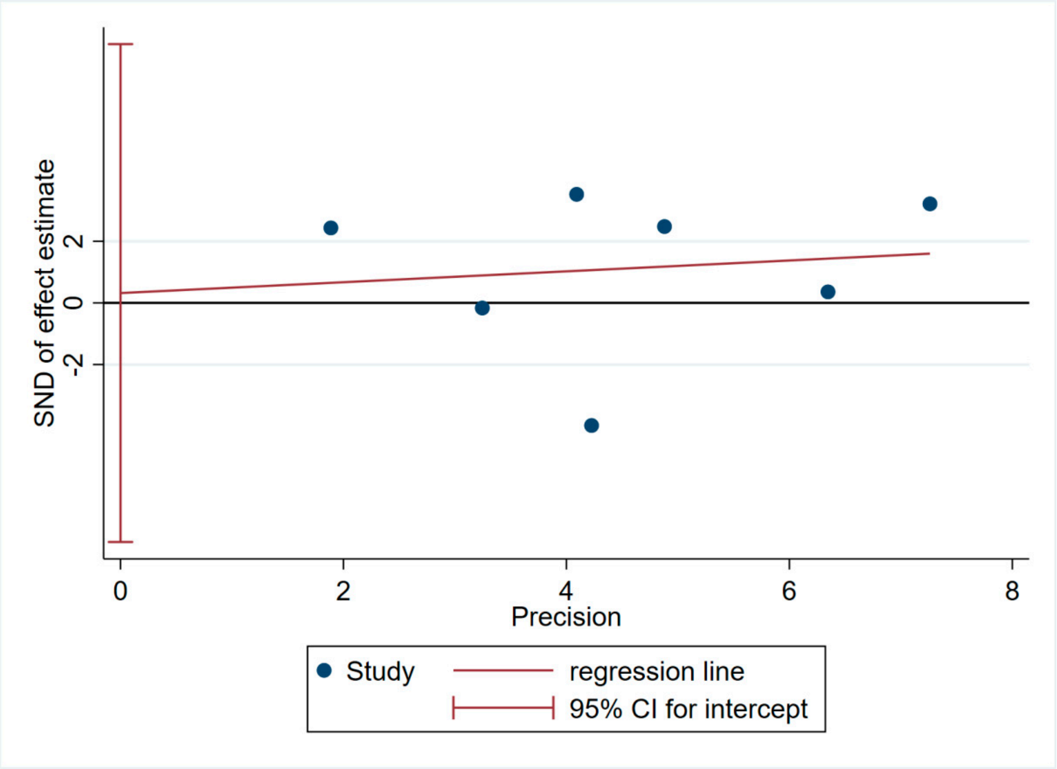

FigureS 99Egger’ s Test of Publication Bias for Studies Assessing Chemotherapy Regimen and PINV

[illegible]

Regress standard normal deviate of intervention
effect estimate against its standard error

.

Number of studies = 7 Root MSE = 2.876

Std_Eff	Coefficient	Std. err.	t	P>|t|	[95% conf.	interval]
slope	.176217	.6464743	0.27	0.796	-1.485598	1.838032
bias	.3195052	3.143566	0.10	0.923	-7.76129	8.4003

Test of H0: no small-study effects P = 0.923

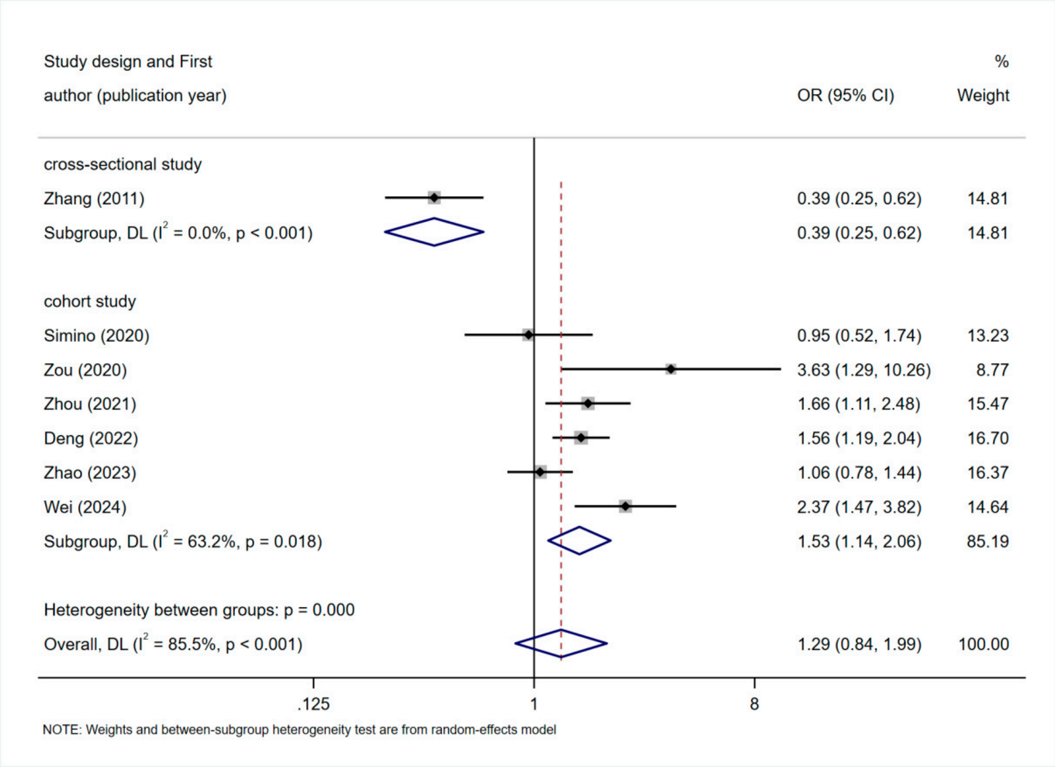

FigureS 100Forest Plot of Subgroup Analysis by study design for the Association Between Chemotherapy Regimen and PINV

Study Design as a Source of Heterogeneity

A subgroup analysis based on study design was conducted to explore sources of heterogeneity.

In the cross-sectional study (Zhang, 2011), the pooled OR was 0.39 (95% CI: 0.25 – 0.62), with no heterogeneity (I^2 = 0.0%, $p < 0.001$), indicating a significant protective association.

In cohort studies (6 studies), the pooled OR was 1.53 (95% CI: 1.14 – 2.06), with moderate heterogeneity (I^2 = 63.2%, $p = 0.018$), showing a significant positive association.

The between-group difference was statistically significant ($p = 0.000$), suggesting that study design may substantially contribute to the observed heterogeneity. The overall heterogeneity across all studies was high (I^2 = 85.5%, $p < 0.001$).

These findings imply that differences in study design could influence the observed effect of chemotherapy regimen on the risk of PINV, potentially due to differences in data collection methods, timing, or control for confounding variables.

pre-chemotherapy nausea

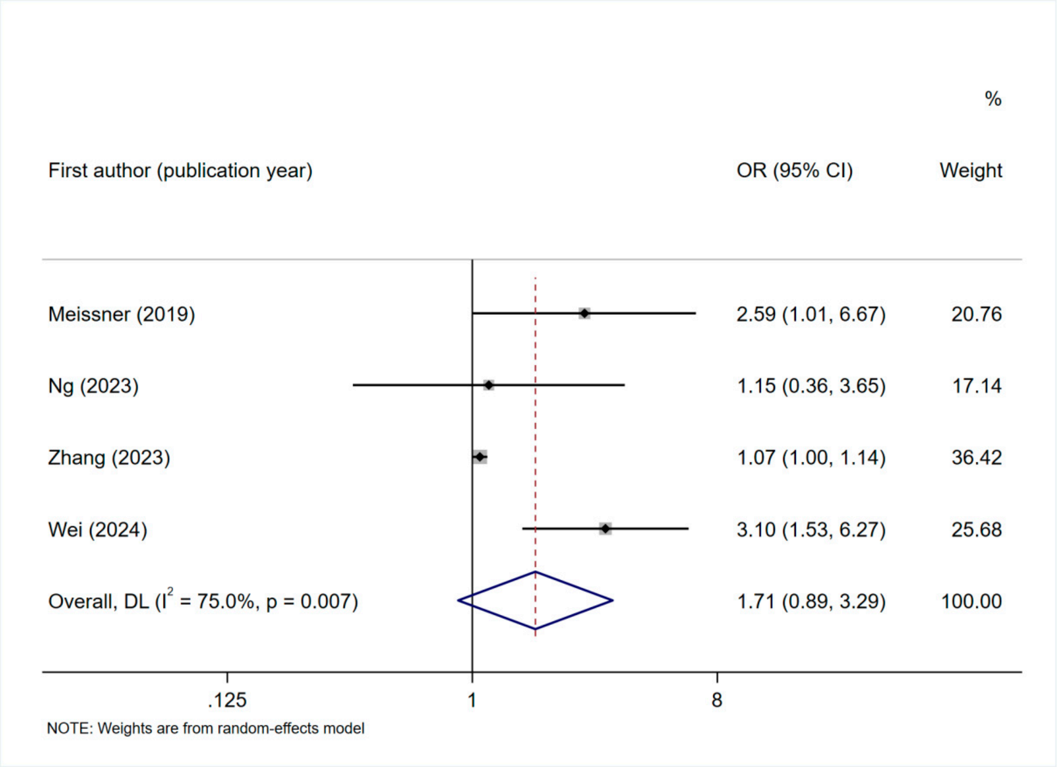

FigureS 101Forest Plot of the Association Between Prechemotherapy Nausea and PINV

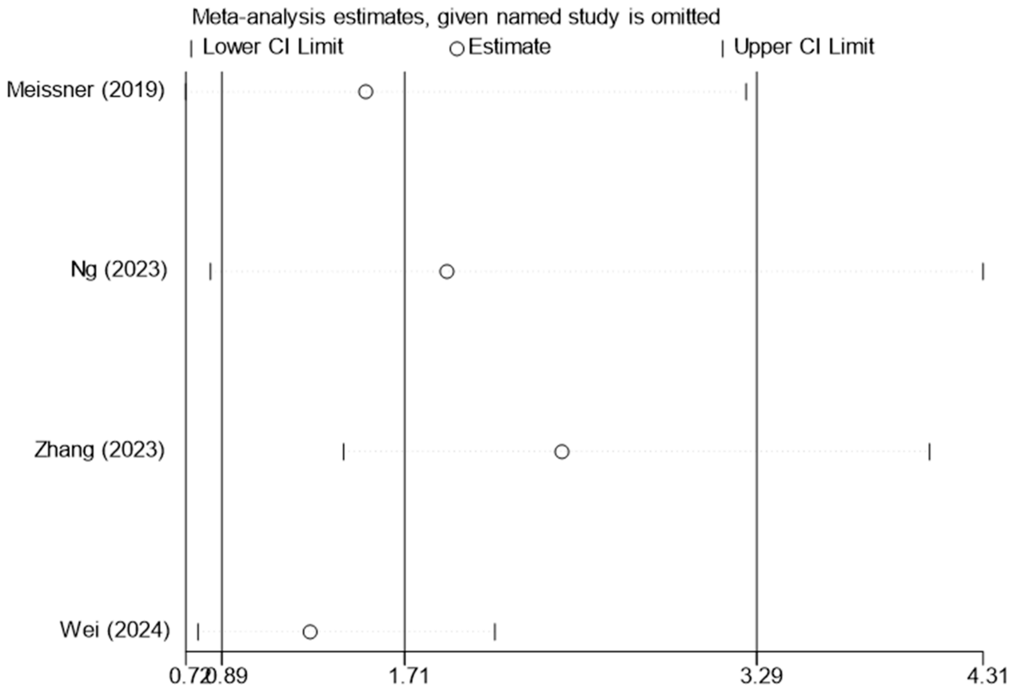

FigureS 102Sensitivity Analysis of the Relationship Between Prechemotherapy Nausea and PINV

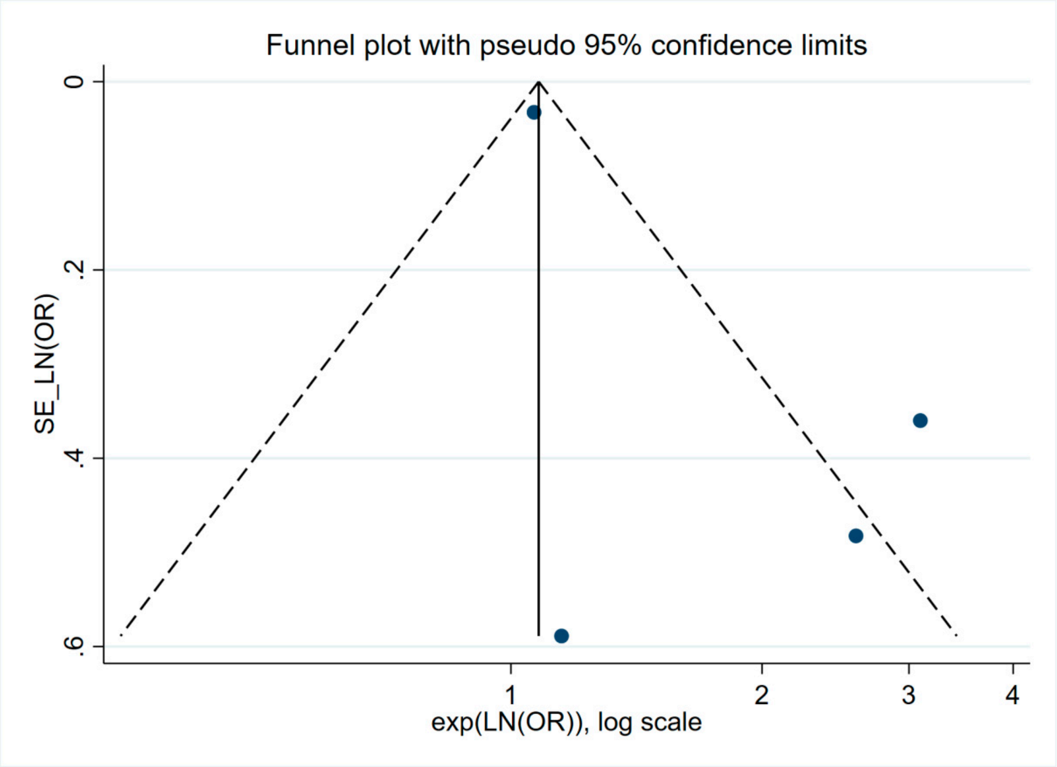

FigureS 103Funnel Plot of Publication Bias in Studies Examining Prechemotherapy Nausea as a Predictor of PINV

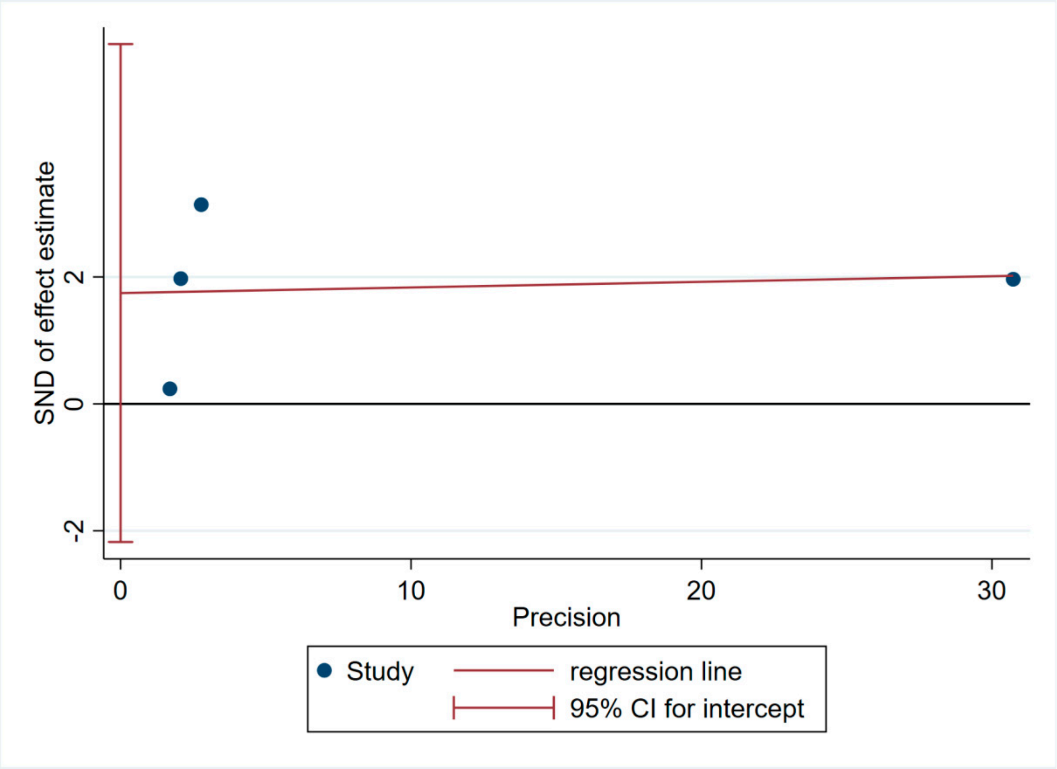

FigureS 104 Egger's Test of Publication Bias for Studies Assessing Prechemotherapy Nausea as a Risk Factor for PINV

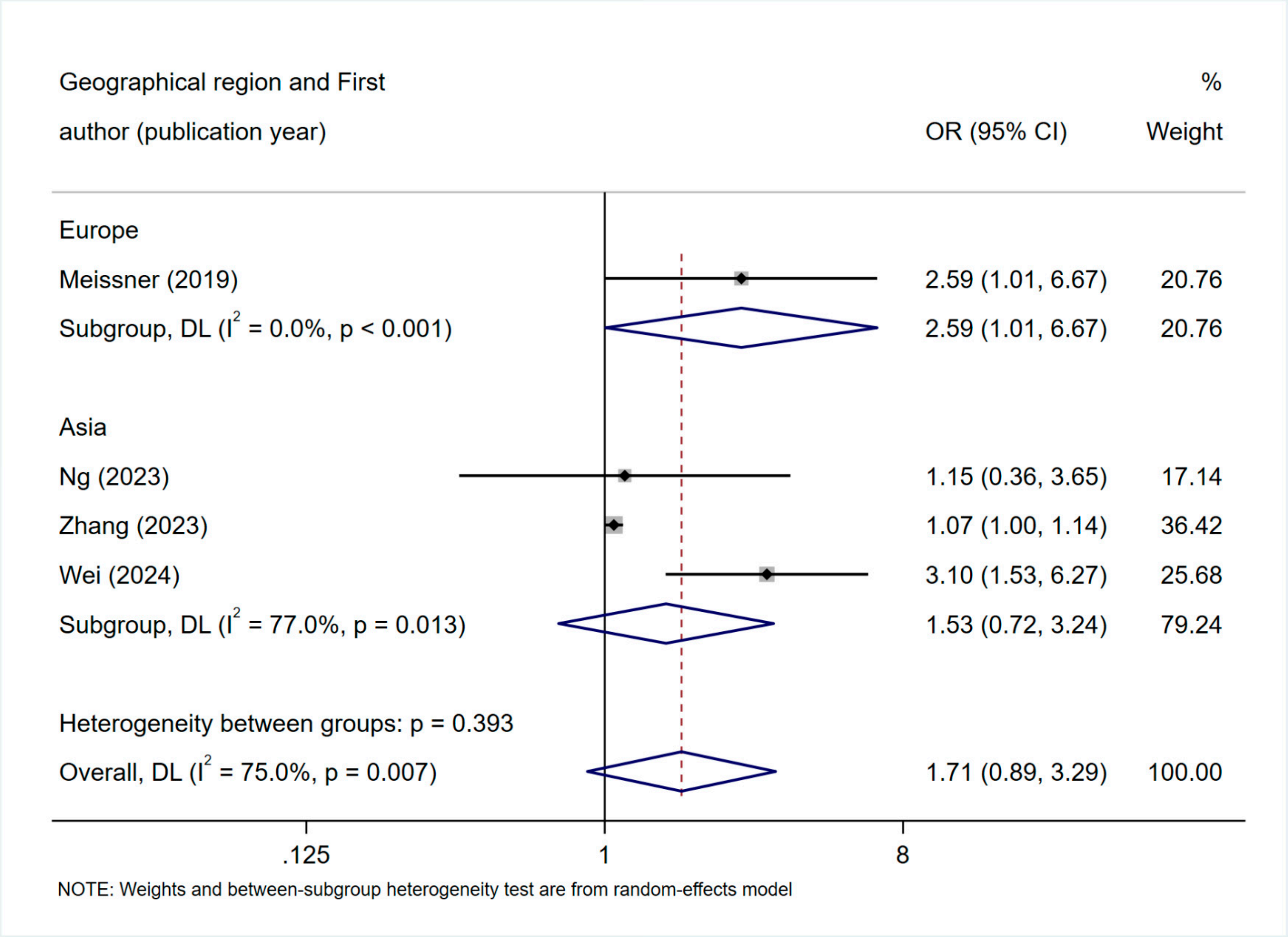
Geographical region and First
author (publication year)
OR (95% CI)
%
Weight
Europe
Meissner (2019)
2.59 (1.01, 6.67)
20.76
Subgroup, DL (I^2 = 0.0%, p < 0.001)
2.59 (1.01, 6.67)
20.76
Asia
Ng (2023)
1.15 (0.36, 3.65)
17.14
Zhang (2023)
1.07 (1.00, 1.14)
36.42
Wei (2024)
3.10 (1.53, 6.27)
25.68
Subgroup, DL (I^2 = 77.0%, p = 0.013)
1.53 (0.72, 3.24)
79.24
Heterogeneity between groups: p = 0.393
Overall, DL (I^2 = 75.0%, p = 0.007)
1.71 (0.89, 3.29)
100.00
.125
1
8
NOTE: Weights and between-subgroup heterogeneity test are from random-effects model

FigureS 105 Forest Plot of Subgroup Analysis by Geographical Region for the Association Between Prechemotherapy Nausea and PINV

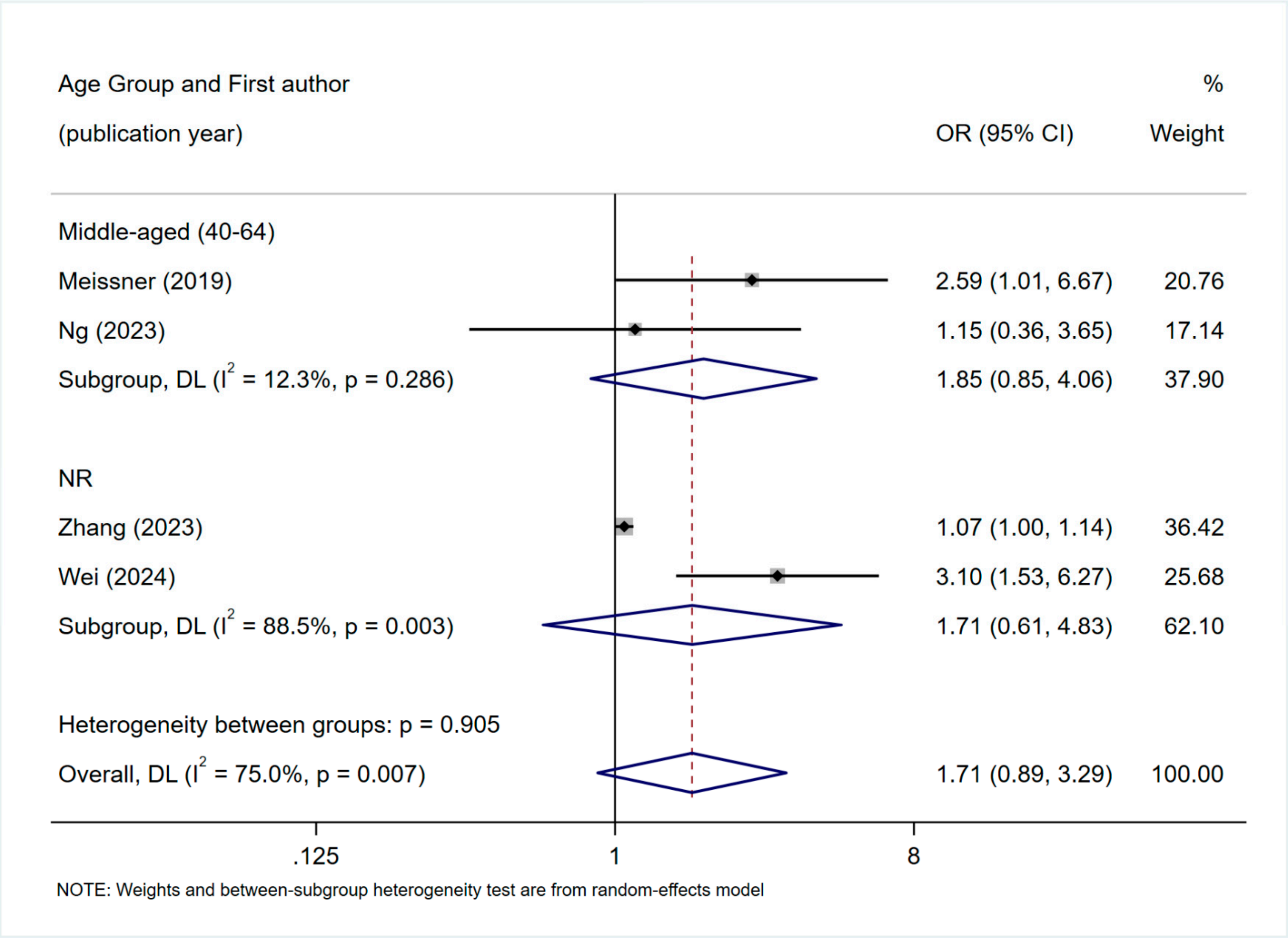
Age Group and First author
(publication year)
OR (95% CI)
% Weight
Middle-aged (40-64)
Meissner (2019) 2.59 (1.01, 6.67) 20.76
Ng (2023) 1.15 (0.36, 3.65) 17.14
Subgroup, DL (I^2 = 12.3%, p = 0.286) 1.85 (0.85, 4.06) 37.90
NR
Zhang (2023) 1.07 (1.00, 1.14) 36.42
Wei (2024) 3.10 (1.53, 6.27) 25.68
Subgroup, DL (I^2 = 88.5%, p = 0.003) 1.71 (0.61, 4.83) 62.10
Heterogeneity between groups: p = 0.905
Overall, DL (I^2 = 75.0%, p = 0.007) 1.71 (0.89, 3.29) 100.00
.125
1
8
NOTE: Weights and between-subgroup heterogeneity test are from random-effects model

FigureS 106 Forest Plot of Subgroup Analysis by Age Group for the Association Between Prechemotherapy Nausea and PINV

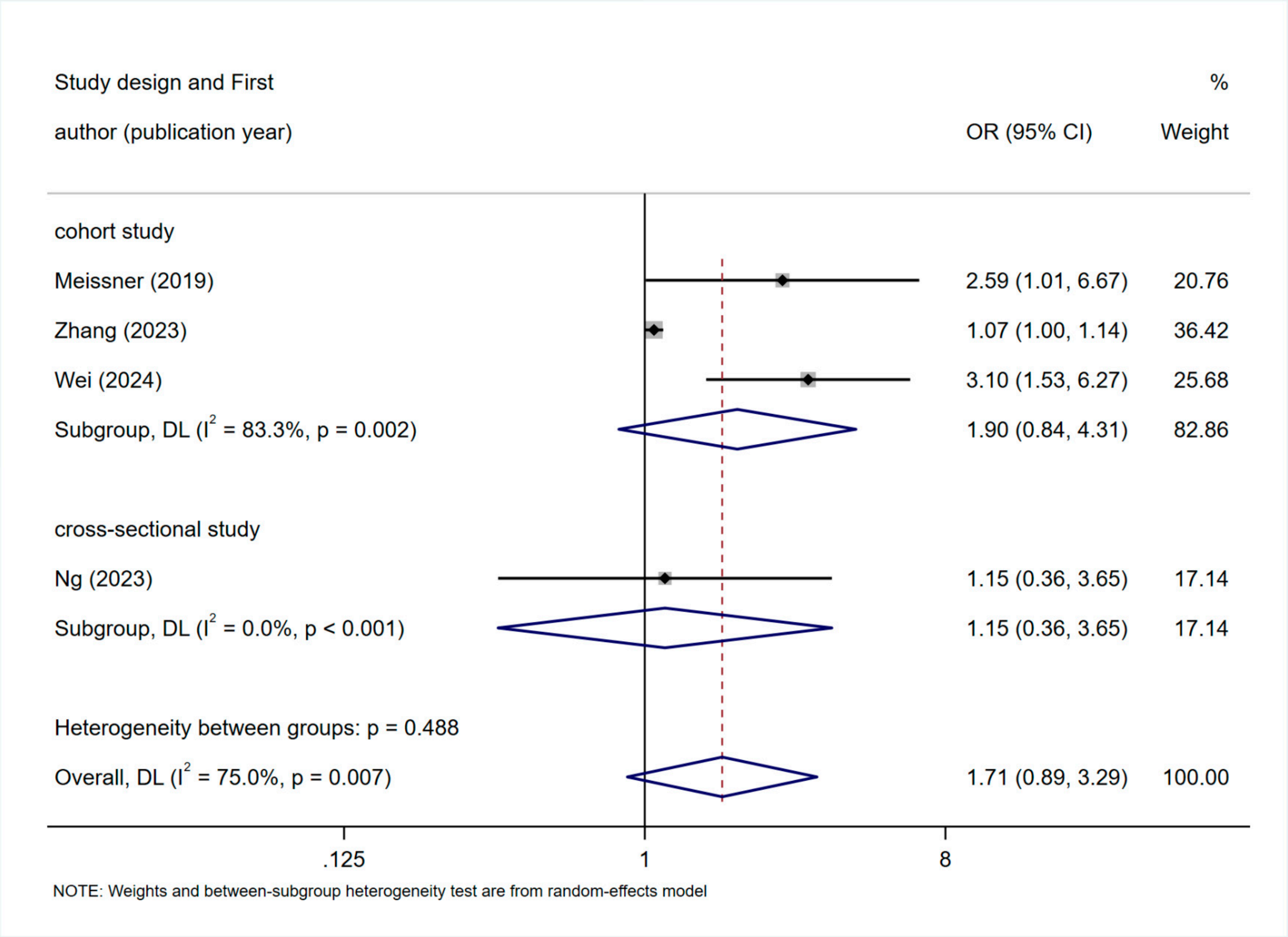
Study design and First
author (publication year)
OR (95% CI)
%
Weight
cohort study
Meissner (2019)
2.59 (1.01, 6.67)
20.76
Zhang (2023)
1.07 (1.00, 1.14)
36.42
Wei (2024)
3.10 (1.53, 6.27)
25.68
Subgroup, DL (I^2 = 83.3%, p = 0.002)
1.90 (0.84, 4.31)
82.86
cross-sectional study
Ng (2023)
1.15 (0.36, 3.65)
17.14
Subgroup, DL (I^2 = 0.0%, p < 0.001)
1.15 (0.36, 3.65)
17.14
Heterogeneity between groups: p = 0.488
Overall, DL (I^2 = 75.0%, p = 0.007)
1.71 (0.89, 3.29)
100.00
.125
1
8
NOTE: Weights and between-subgroup heterogeneity test are from random-effects model

FigureS 107Forest Plot of Subgroup Analysis by Study design for the Association Between Prechemotherapy Nausea and PINV

Sources of Heterogeneity：

Despite extensive efforts, including sensitivity analysis, meta-regression, and subgroup analysis, the source of heterogeneity remained unexplained, potentially due to unmeasured confounders or random variation.

Expectation of nausea

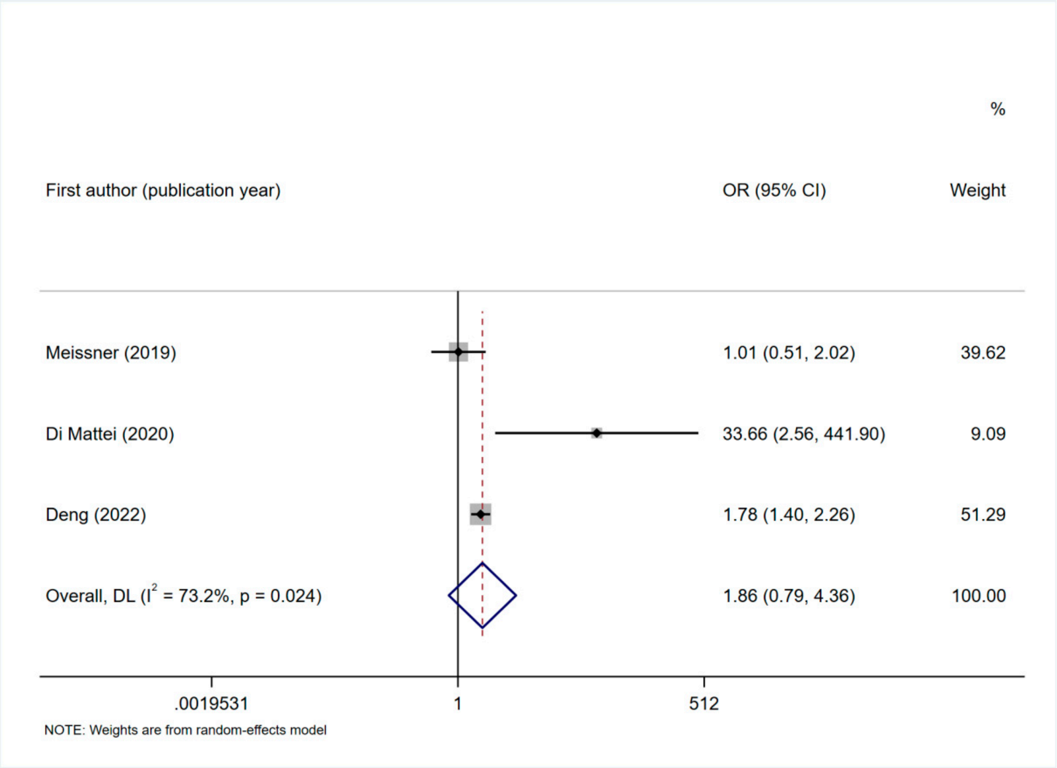

FigureS 108 Forest Plot of the Association Between Expectation of Nausea and PINV

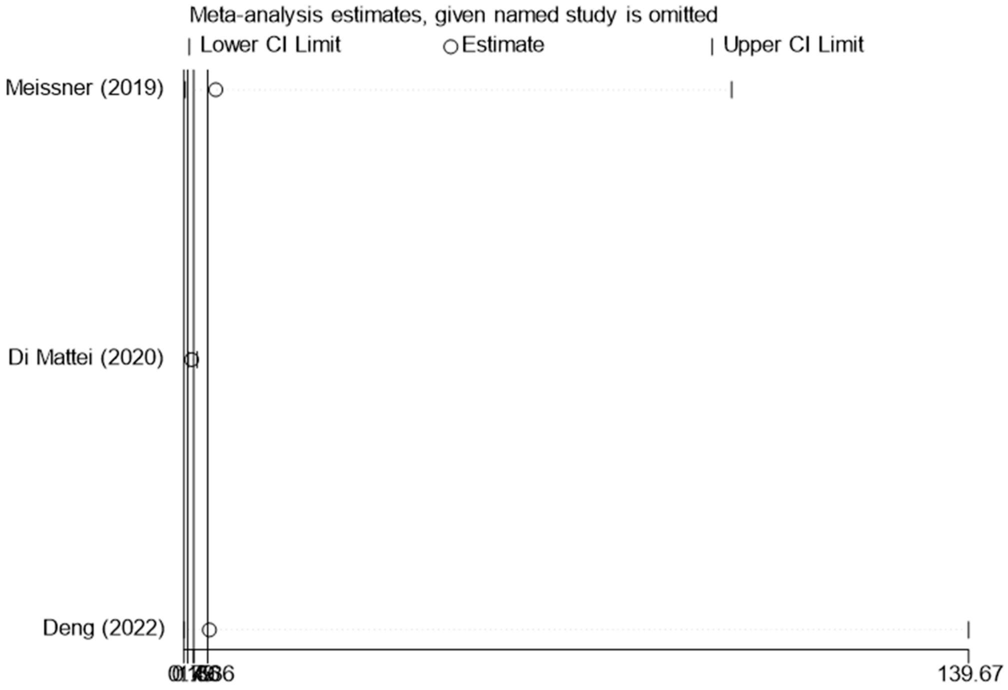

FigureS 109 Sensitivity Analysis of the Relationship Between Expectation of Nausea and PINV

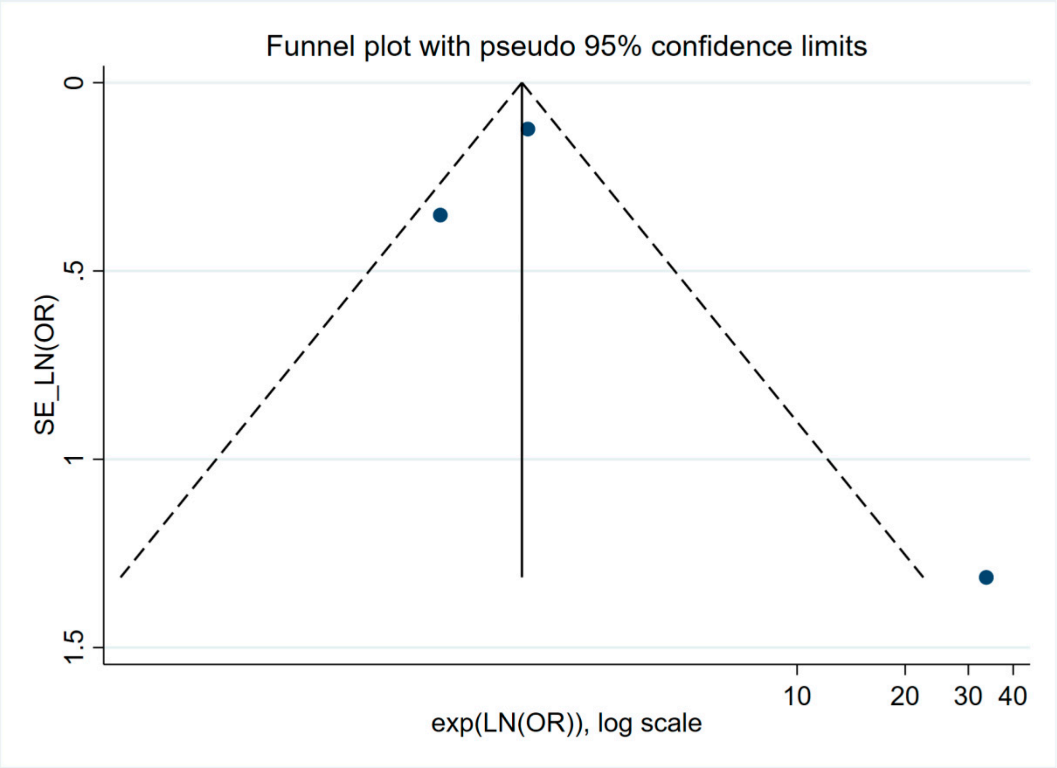

FigureS 110Funnel Plot of Publication Bias in Studies Examining Expectation of Nausea as a Predictor of PINV

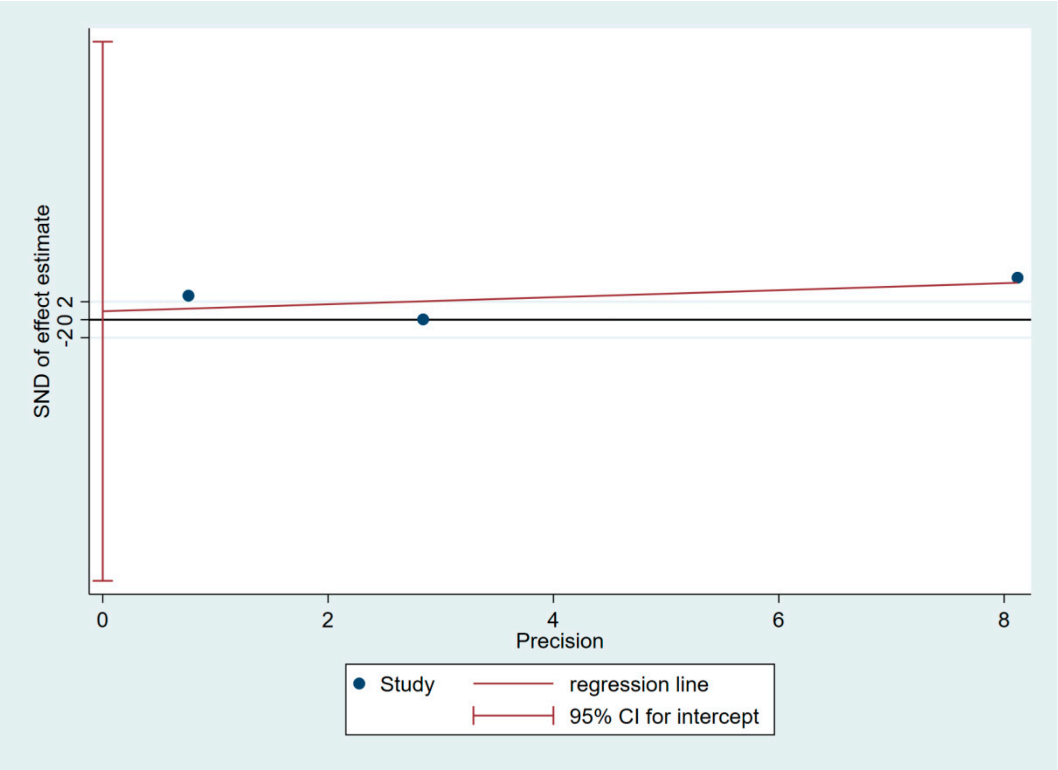

FigureS 111 Egger’ s Test of Publication Bias for Studies Assessing Expectation of Nausea as a Risk Factor for PINV

```
Egger's test for small-study effects:
Regress standard normal deviate of intervention
  effect estimate against its standard error

.
Number of studies =  3                                  Root MSE      =   2.536
------------------------------------------------------------------------------
     Std_Eff | Coefficient  Std. err.      t    P>|t|     [95% conf. interval]
-------------+----------------------------------------------------------------
       slope |   .3896994    .472829     0.82   0.561    -5.618163    6.397562
        bias |   .9399262    2.35765     0.40   0.758    -29.01686    30.89671
------------------------------------------------------------------------------

Test of H0: no small-study effects          P = 0.758
```

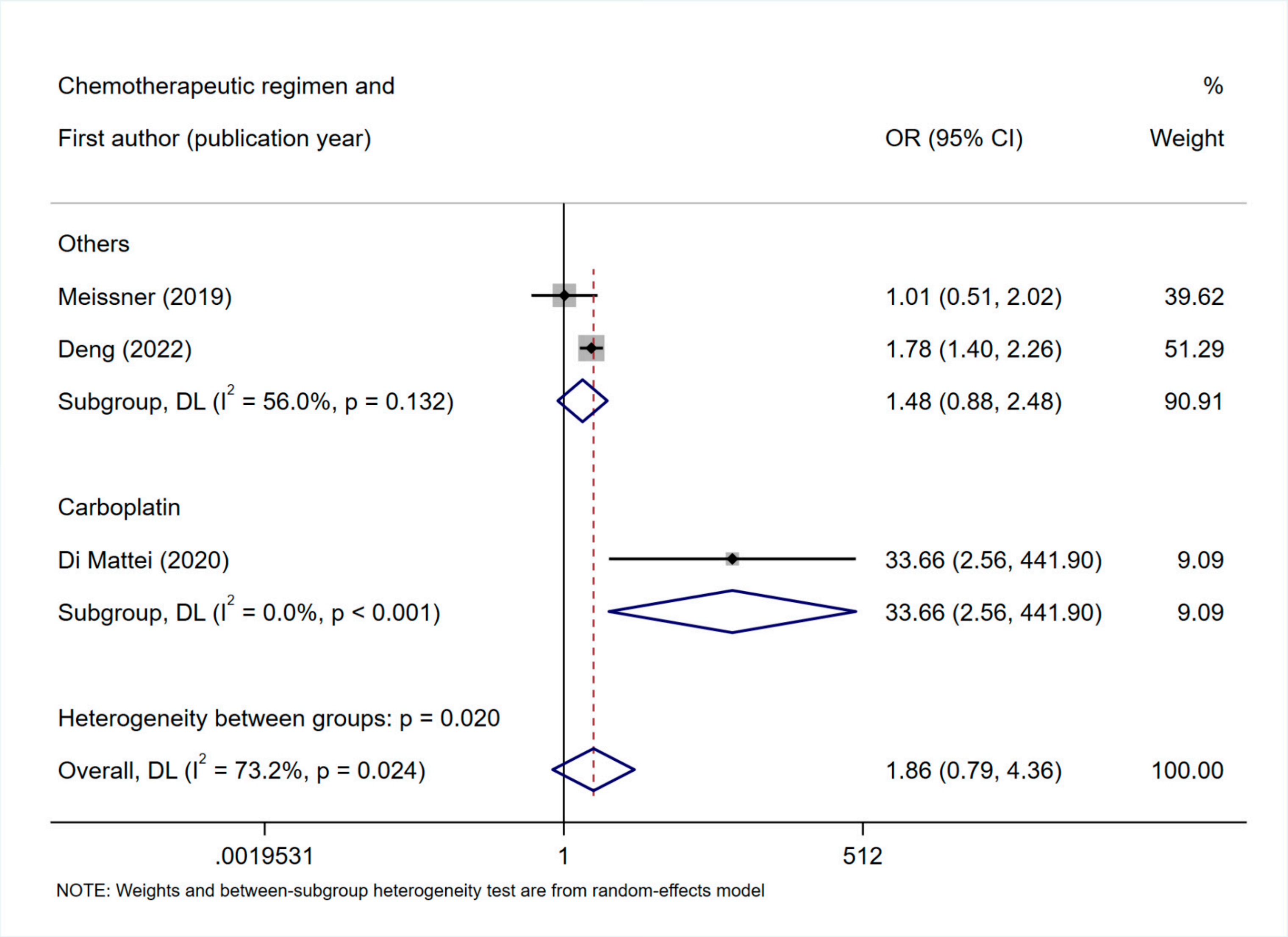
Chemotherapeutic regimen and
First author (publication year)
OR (95% CI)
%
Weight
Others
Meissner (2019)
1.01 (0.51, 2.02)
39.62
Deng (2022)
1.78 (1.40, 2.26)
51.29
Subgroup, DL (I² = 56.0%, p = 0.132)
1.48 (0.88, 2.48)
90.91
Carboplatin
Di Mattei (2020)
33.66 (2.56, 441.90)
9.09
Subgroup, DL (I² = 0.0%, p < 0.001)
33.66 (2.56, 441.90)
9.09
Heterogeneity between groups: p = 0.020
Overall, DL (I² = 73.2%, p = 0.024)
1.86 (0.79, 4.36)
100.00
.0019531
1
512
NOTE: Weights and between-subgroup heterogeneity test are from random-effects model

FigureS 112Forest Plot of Subgroup Analysis by Chemotherapeutic regimen for the Association Between Expectation of Nausea and PINV

Study Region as a Source of Heterogeneity:

Chemotherapeutic Regimen as a Source of Heterogeneity

A subgroup analysis based on chemotherapeutic regimen was conducted to explore heterogeneity in the association between expectation of nausea and PINV.

Others (non-carboplatin regimens):

Two studies (Meissner, 2019; Deng, 2022) were included. The pooled OR was 1.48 (95% CI: 0.88–2.48), with moderate heterogeneity (I^2 = 56.0%, p = 0.132).

Carboplatin-based regimen:

One study (Di Mattei, 2020) reported a significantly elevated risk with an OR of 33.66 (95% CI: 2.56–441.90), with no within-group heterogeneity (I^2 = 0.0%, $p < 0.001$).

The between-group difference was statistically significant (p = 0.020), suggesting that chemotherapeutic regimen may be a source of heterogeneity. The overall heterogeneity was substantial (I^2 = 73.2%, p = 0.024). These findings indicate that the association between expectation of nausea and PINV may differ depending on the type of chemotherapy, particularly with extreme effects observed in carboplatin-treated populations.

Antiemetic regimen

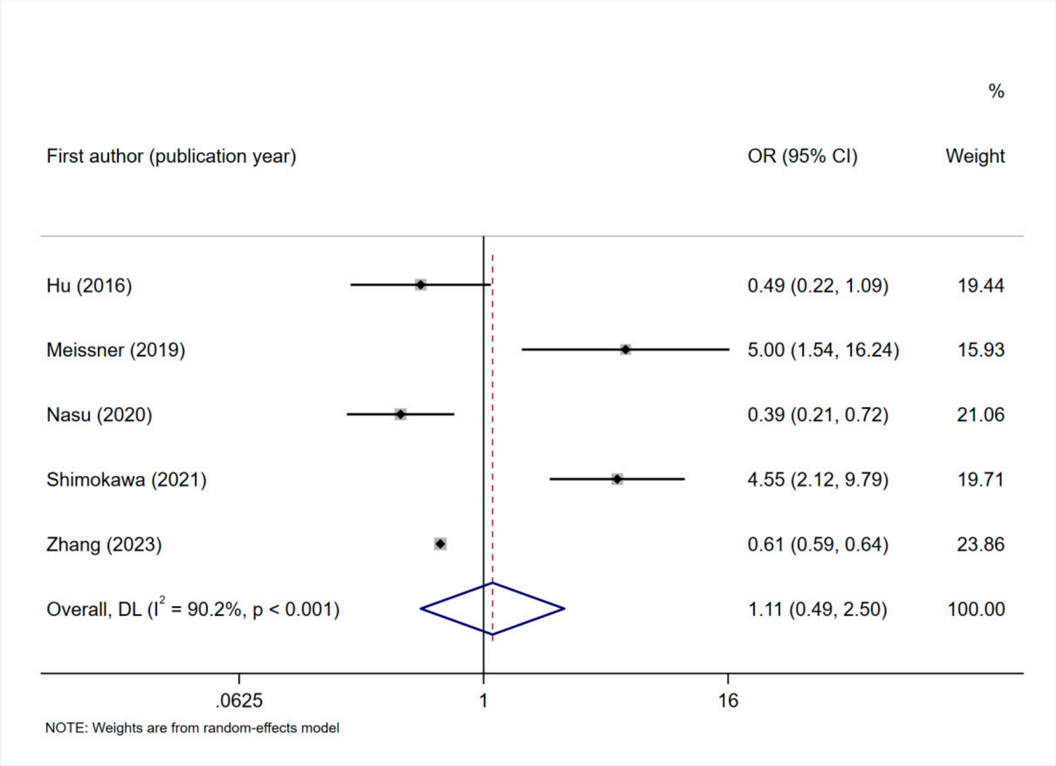

FigureS 113Forest Plot of the Association Between Antiemetic Regimen and PINV

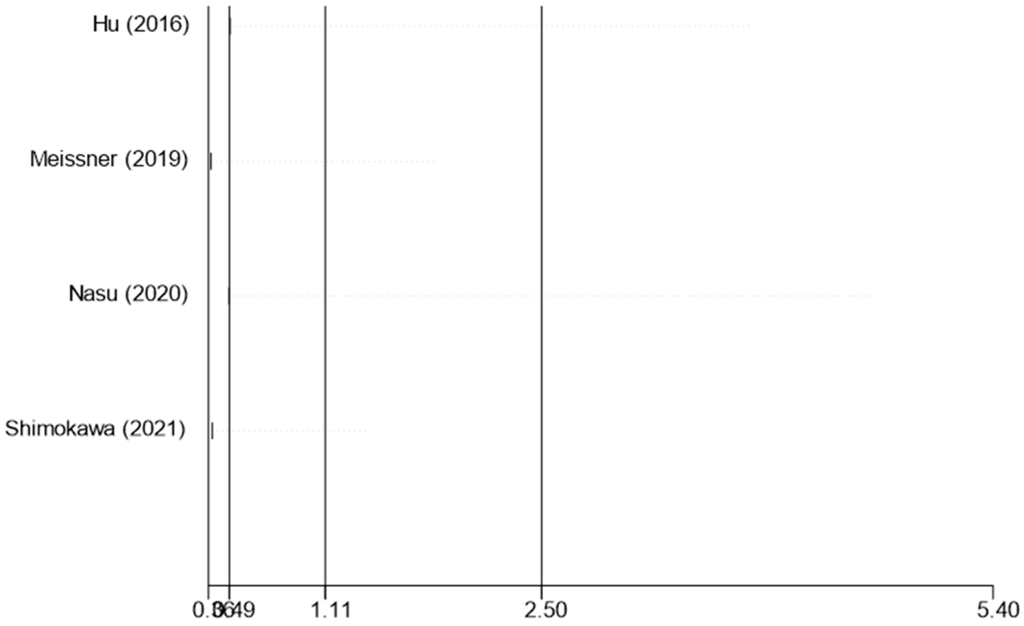

FigureS 114 Sensitivity Analysis of the Impact of Antiemetic Regimen on PINV

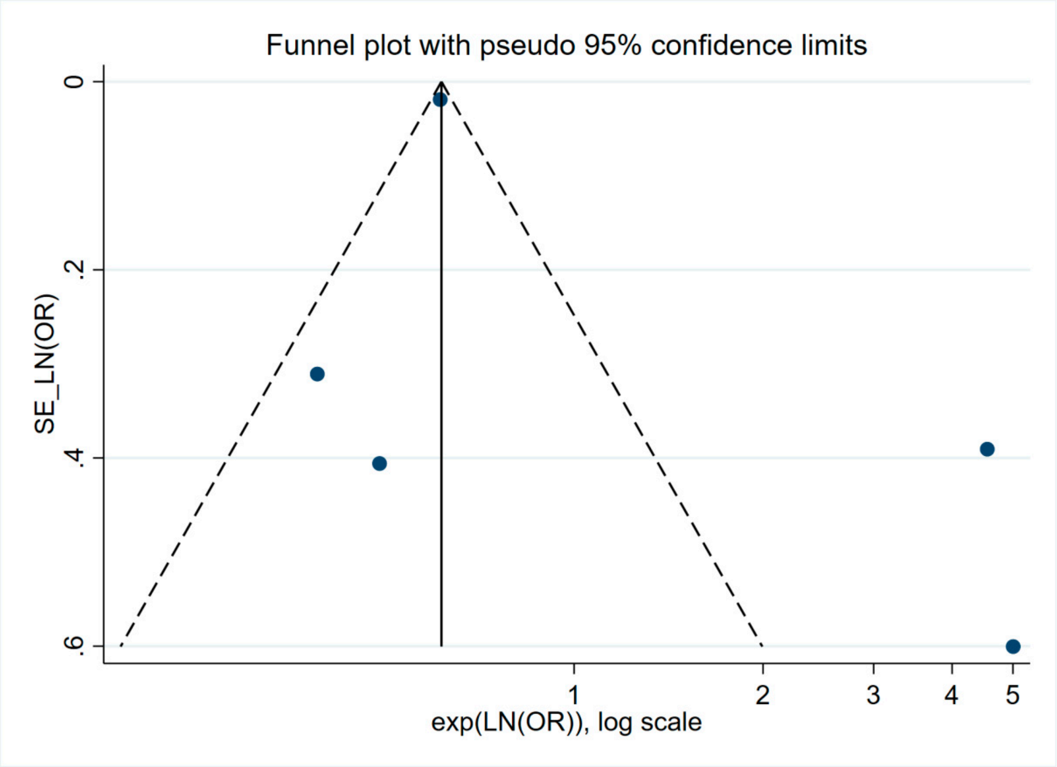

FigureS 115Funnel Plot of Publication Bias in Studies Examining the Effect of Antiemetic Regimens on PINV

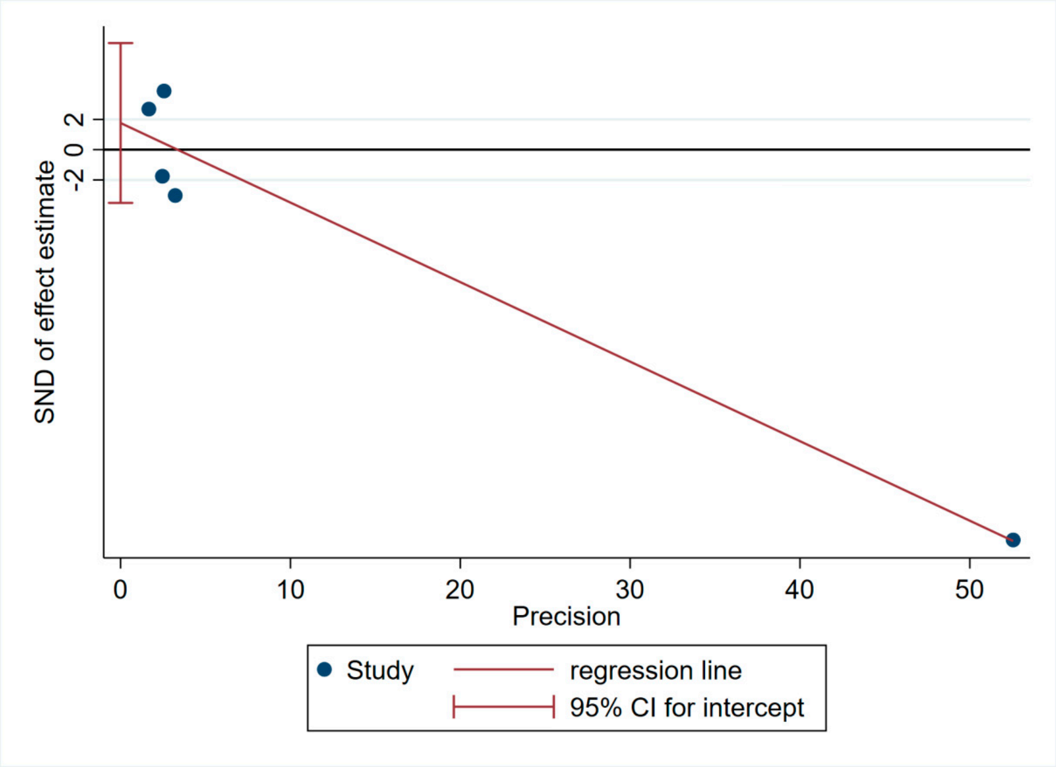

SND of effect estimate
-2
0
2
0
10
20
30
40
50
Precision
Study
regression line
95% CI for intercept

FigureS 116Egger ’ s Test of Publication Bias for Studies Assessing Antiemetic Regimen Effectiveness in PINV

```
Egger's test for small-study effects:
Regress standard normal deviate of intervention
  effect estimate against its standard error

.
Number of studies =  5                             Root MSE      =   3.151

    Std_Eff   Coefficient  Std. err.      t    P>|t|     [95% conf. interval]

      slope    -.5258762    .070319    -7.48   0.005    -.7496625   -.3020898
       bias      1.76305   1.660408     1.06   0.366    -3.521108    7.047209

Test of H0: no small-study effects          P = 0.366
```

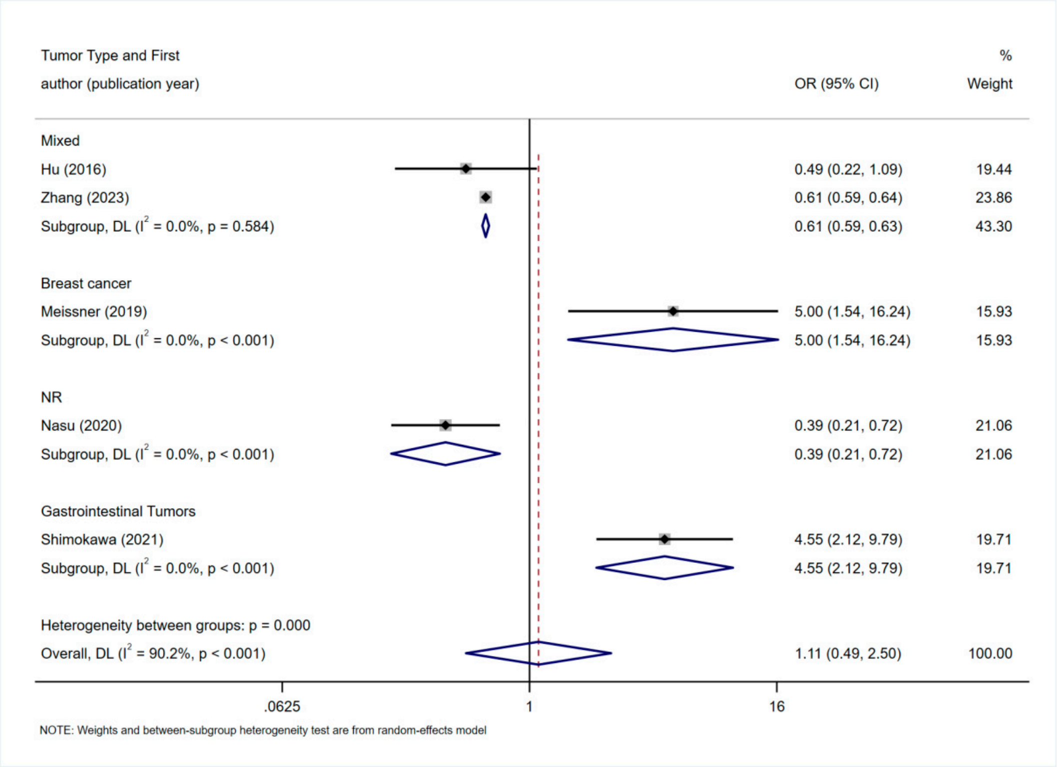

FigureS 117Forest Plot of Subgroup Analysis by Tumor Type for the Association Between Antiemetic Regimen and PINV

Sources of Heterogeneity：

Tumor Type as a Source of Heterogeneity

A subgroup analysis was performed based on tumor type to explore heterogeneity in the association between antiemetic regimen and platinum-based chemotherapy-induced nausea and vomiting (PINV).

Mixed tumors (Hu, 2016; Zhang, 2023):

The pooled OR was 0.61 (95% CI: 0.59–0.63), with no heterogeneity (I^2 = 0.0%, p = 0.584), suggesting a consistent and protective effect of antiemetic regimen.

Breast cancer (Meissner, 2019):

OR = 5.00 (95% CI: 1.54–16.24), indicating a strong positive association.

NR (tumor type not reported, Nasu, 2020):

OR = 0.39 (95% CI: 0.21–0.72), with no heterogeneity.

Gastrointestinal tumors (Shimokawa, 2021):

OR = 4.55 (95% CI: 2.12–9.79), also indicating a strong association.

The between-group heterogeneity was significant (p = 0.000), with high overall heterogeneity across studies (I^2 = 90.2%, $p < 0.001$). These results suggest that tumor type may significantly influence the observed effect of antiemetic regimen on the risk of PINV, potentially due to differing emetogenic risks, treatment strategies, or patient susceptibility across tumor categories.

FigureS 118Bayesian meta-analysis of nausea incidence using MAGEC method.

Study	N	Posterior median (95% Cr
2011-Liu	459	40.80 (36.40, 45.25)
2012-Hilarius	275	67.45 (61.83, 72.65)
2012-Chan	156	50.17 (42.57, 57.74)
2024-Wei	500	47.29 (42.99, 51.64)
2021-Shimokawa	661	37.88 (34.23, 41.60)
2012-Wei	400	40.06 (35.40, 44.86)
2013-Poon	473	68.80 (64.56, 72.80)
2015-lv	345	60.34 (55.18, 65.37)
2016-Hu	881	40.09 (36.89, 43.36)
2017-Baba	192	44.21 (37.46, 51.04)
2017-Yao	112	40.38 (31.86, 49.22)
2018-Yokoi	156	71.81 (64.65, 78.28)
2019-Chai	643	55.17 (51.37, 58.94)
2019-Meissner	121	64.29 (55.92, 72.14)
2020-Di Mattei	81	78.99 (69.93, 86.46)
2020-Nasu	314	43.93 (38.52, 49.35)
2020-Simino	269	57.81 (51.99, 63.49)
2021-Huang	400	53.26 (48.36, 58.02)
2021-Zhou	1000	36.18 (33.27, 39.17)
2022-Deng	2215	28.93 (27.05, 30.82)
2023-Ng	137	43.68 (35.91, 51.81)
2023-Zhang	320	41.33 (36.08, 46.71)
2023-Zhao	720	48.81 (45.18, 52.47)
2024-Ostwal	560	67.75 (63.82, 71.50)
2011-Zhang	831	76.82 (73.87, 79.58)
Overall	12221	52.60 (46.21, 59.00)

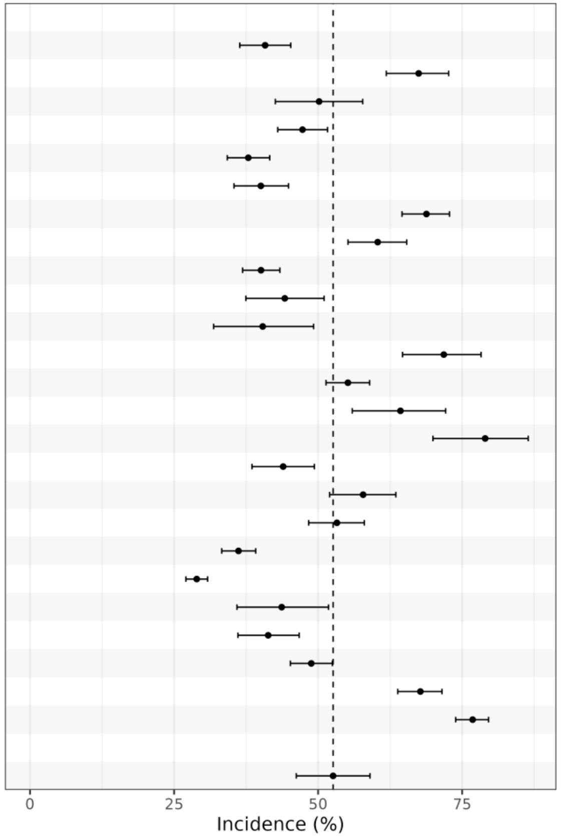

Bayesian meta-analysis of nausea incidence using the MAGEC method.

A Bayesian meta-analysis of nausea incidence was conducted using the MAGEC method to account for potential underreporting due to left-censored adverse event data. The posterior median of the overall nausea incidence was 52.6%, with a 95% credible interval (CI) of 46.2%–59.0%. The estimated between-study standard deviation (SD) was 0.63 (95% CI: 0.47–0.88), indicating moderate heterogeneity. The 95% predictive CI for a future study ranged from 23.4% to 80.5%, suggesting wide variability in real-world settings. A complete-case sensitivity analysis excluding studies with censored AE data yielded the same pooled estimate of 52.6% (95% CI: 46.2%–59.0%), supporting the robustness of the model despite possible reporting bias.

Study	N	Posterior median (95% Cr
2011-Liu	459	40.43 (36.05, 44.98)
2011-Zhang	831	62.14 (58.80, 65.37)
2012-Chan	156	17.45 (12.16, 23.73)
2012-Hilarius	275	23.16 (18.49, 28.31)
2012-Wei	400	39.66 (34.98, 44.55)
2013-Poon	473	52.43 (47.88, 56.83)
2015-lv	345	60.12 (54.97, 65.18)
2016-Hu	881	39.93 (36.74, 43.15)
2017-Baba	192	16.31 (11.66, 21.89)
2017-Yao	112	31.37 (23.47, 40.13)
2018-Yokoi	156	71.73 (64.34, 78.31)
2019-Chai	643	55.02 (51.20, 58.85)
2020-Nasu	314	15.45 (11.83, 19.62)
2020-Simino	269	32.73 (27.39, 38.39)
2021-Huang	400	52.95 (48.08, 57.77)
2021-Zhou	1000	36.00 (33.04, 39.00)
2021-Shimokawa	661	10.46 (8.30, 12.91)
2022-Deng	2215	28.81 (26.96, 30.73)
2023-Ng	137	42.64 (34.71, 50.84)
2023-Zhang	320	29.46 (24.63, 34.50)
2023-Zhao	720	22.20 (19.27, 25.36)
2024-Ostwal	560	23.18 (19.86, 26.75)
2024-Wei	500	47.06 (42.75, 51.42)
Overall	12019	35.26 (27.71, 43.59)

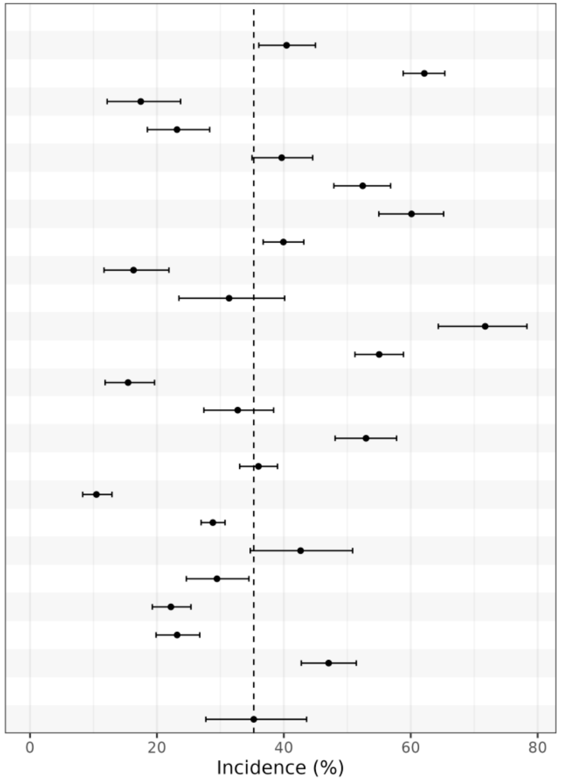

FigureS 119Bayesian meta-analysis of vomiting incidence using MAGEC method.

Bayesian meta-analysis of vomiting incidence using the MAGEC method.

A Bayesian meta-analysis of vomiting incidence was performed using the MAGEC method to adjust for potential underreporting due to left-censored adverse event data. The posterior median of the overall incidence was 35.3% with a 95% credible interval (CI) of 27.7%–43.6%. The estimated between-study standard deviation (SD) was 0.82 (95% CI: 0.62–1.16), indicating moderate-to-high heterogeneity. The 95% predictive CI for a new study ranged from 8.9% to 75.8%, reflecting considerable variability across populations and settings. A supplementary complete-case analysis excluding censored studies produced the same pooled estimate of 35.3% (95% CI: 27.7%–43.6%), supporting the robustness of the findings despite potential bias.
